# Environmental effects of stratospheric ozone depletion, UV radiation, and interactions with climate change: UNEP Environmental Effects Assessment Panel, Update 2020

**DOI:** 10.1007/s43630-020-00001-x

**Published:** 2021-01-20

**Authors:** R. E. Neale, P. W. Barnes, T. M. Robson, P. J. Neale, C. E. Williamson, R. G. Zepp, S. R. Wilson, S. Madronich, A. L. Andrady, A. M. Heikkilä, G. H. Bernhard, A. F. Bais, P. J. Aucamp, A. T. Banaszak, J. F. Bornman, L. S. Bruckman, S. N. Byrne, B. Foereid, D.-P. Häder, L. M. Hollestein, W.-C. Hou, S. Hylander, M. A. K. Jansen, A. R. Klekociuk, J. B. Liley, J. Longstreth, R. M. Lucas, J. Martinez-Abaigar, K. McNeill, C. M. Olsen, K. K. Pandey, L. E. Rhodes, S. A. Robinson, K. C. Rose, T. Schikowski, K. R. Solomon, B. Sulzberger, J. E. Ukpebor, Q.-W. Wang, S.-Å. Wängberg, C. C. White, S. Yazar, A. R. Young, P. J. Young, L. Zhu, M. Zhu

**Affiliations:** 1 Population Health Department, QIMR Berghofer Medical Research Institute, Brisbane, Australia; 2 Biological Sciences and Environmental Program, Loyola University New Orleans, New Orleans, LA USA; 3 Organismal and Evolutionary Biology (OEB), Viikki Plant Sciences Centre (ViPS), University of Helsinki, Helsinki, Finland; 4 Smithsonian Environmental Research Center, Maryland, USA; 5 Department of Biology, Miami University, Oxford, OH USA; 6 ORD/CEMM, US Environmental Protection Agency, Athens, GA USA; 7 School of Earth, Atmospheric and Life Sciences, University of Wollongong, Wollongong, Australia; 8 Atmospheric Chemistry Observations and Modeling Laboratory, National Center for Atmospheric Research, Boulder, CO USA; 9 Chemical and Biomolecular Engineering, North Carolina State University, Raleigh, NC USA; 10 Finnish Meteorological Institute, Helsinki, Finland; 11 Biospherical Instruments Inc, San Diego, CA USA; 12 Department of Physics, Laboratory of Atmospheric Physics, Aristotle University, Thessaloniki, Greece; 13 Ptersa Environmental Consultants, Pretoria, South Africa; 14 Unidad Académica de Sistemas Arrecifales, Universidad Nacional Autónoma de México, Puerto Morelos, México; 15 Food Futures Institute, Murdoch University, Perth, Australia; 16 Department of Materials Science and Engineering, Case Western Reserve University, Cleveland, OH USA; 17 The University of Sydney, School of Medical Sciences, Discipline of Applied Medical Science, Sydney, Australia; 18 Environment and Natural Resources, Norwegian Institute of Bioeconomy Research, Ås, Norway; 19 Department of Biology, Friedrich-Alexander University, Möhrendorf, Germany; 20 Department of Dermatology, Erasmus MC Cancer Institute, Rotterdam, The Netherlands; 21 Department of Environmental Engineering, National Cheng Kung University, Tainan, Taiwan, Republic of China; 22 Centre for Ecology and Evolution in Microbial model Systems–EEMiS, Linnaeus University, Kalmar, Sweden; 23 School of BEES, Environmental Research Institute, University College Cork, Cork, Ireland; 24 Antarctic Climate Program, Australian Antarctic Division, Kingston, Australia; 25 National Institute of Water and Atmospheric Research, Lauder, New Zealand; 26 The Institute for Global Risk Research, LLC, Bethesda, MD USA; 27 National Centre of Epidemiology and Population Health, Australian National University, Canberra, Australia; 28 Faculty of Science and Technology, University of La Rioja, Logroño, Spain; 29 ETH-Zurich, Zurich, Switzerland; 30 Cancer Control Group, QIMR Berghofer Medical Research Institute, Brisbane, Australia; 31 Department of Wood Properties and Uses, Institute of Wood Science and Technology, Bangalore, India; 32 Photobiology Unit, Dermatology Research Centre, School of Biological Sciences, Faculty of Biology Medicine and Health, University of Manchester, Manchester, UK; 33 Securing Antarctica’s Environmental Future, Global Challenges Program and School of Earth, Atmospheric and Life Sciences, University of Wollongong, Wollongong, Australia; 34 Department of Biological Sciences, Rensselaer Polytechnic Institute, Troy, NY USA; 35 IUF-Leibniz Institute of Environmental Medicine, Dusseldorf, Germany; 36 Centre for Toxicology, School of Environmental Sciences, University of Guelph, Guelph, Canada; 37 Academic Guest Eawag: Swiss Federal Institute of Aquatic Science and Technology, Duebendorf, Switzerland; 38 Chemistry Department, Faculty of Physical Sciences, University of Benin, Benin City, Nigeria; 39 Institute of Applied Ecology, Chinese Academy of Sciences (CAS), Shenyang, China; 40 Department of Marine Sciences, University of Gothenburg, Gothenburg, Sweden; 41 Bee America, 5409 Mohican Rd, Bethesda, MD USA; 42 Garvan Institute of Medical Research, Sydney, Australia; 43 St John’s Institute of Dermatology, King’s College London, London, UK; 44 Lancaster Environment Centre, Lancaster University, Lancaster, UK; 45 Center for Advanced Low-Dimension Materials, Donghua University, Shanghai, China; 46 State Key Laboratory for Modification of Chemical Fibers and Polymer Materials, Donghua University, Shanghai, China

## Abstract

This assessment by the Environmental Effects Assessment Panel (EEAP) of the United Nations Environment Programme (UNEP) provides the latest scientific update since our most recent comprehensive assessment (Photochemical and Photobiological Sciences, 2019, 18, 595–828). The interactive effects between the stratospheric ozone layer, solar ultraviolet (UV) radiation, and climate change are presented within the framework of the Montreal Protocol and the United Nations Sustainable Development Goals. We address how these global environmental changes affect the atmosphere and air quality; human health; terrestrial and aquatic ecosystems; biogeochemical cycles; and materials used in outdoor construction, solar energy technologies, and fabrics. In many cases, there is a growing influence from changes in seasonality and extreme events due to climate change. Additionally, we assess the transmission and environmental effects of the severe acute respiratory syndrome coronavirus 2 (SARS-CoV-2), which is responsible for the COVID-19 pandemic, in the context of linkages with solar UV radiation and the Montreal Protocol.

**Electronic supplementary material:**

The online version of this article (10.1007/s43630-020-00001-x) contains supplementary material, which is available to authorized users.

## Introduction

**Fig. 1:**
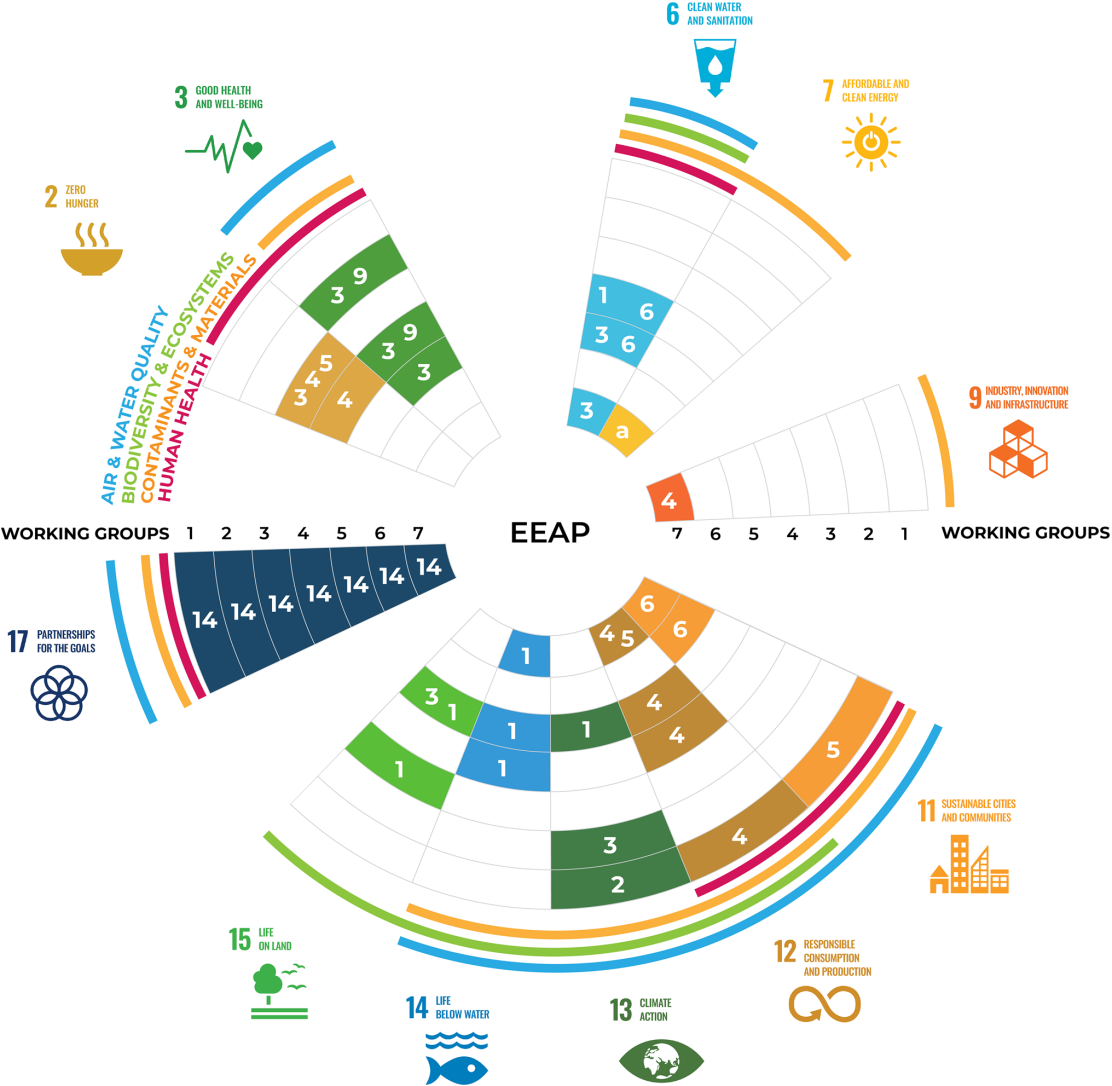
The Sustainable Development Goals (SDG) relevant to this assessment are shown outside the circle with specific targets (numbers in white) linked to EEAP Working Groups (1–7, numbers in black) in concentric circles. These SDG targets include: 2.3 increase productivity of small-scale food producers; 2.4 ensure sustainable food production systems; 2.5 maintain genetic diversity of agricultural plants and animals; 3.3 end epidemics of communicable diseases; 3.9 reduce deaths caused by air; soil and water contamination; 6.1 achieve access to safe drinking water; 6.3 reduce water pollution; 6.6 protect water-related ecosystems; 7.A enhance international cooperation around clean energy; 9.4 upgrade industries to be sustainable; 11.5 reduce deaths caused by disasters; 11.6 reduce the environmental impact of cities; 12.4 achieve environmentally sound management of chemicals and wastes; 12.5 reduce waste generation; 13.1 Strengthen resilience to climate-related hazards and disasters; 13.2 integrate climate change measures into policy; strategy and planning; 13.3 improve education on climate-change mitigation; 14.1 reduce marine pollution; 14.3  minimise impacts of ocean acidification; 15.1 ensure the conservation of terrestrial ecosystems; 15.3 combat desertification; and 17.14 enhance policy coherence for sustainable development. Topics covered by the EEAP Working Groups 1–7 are: (1) Stratospheric ozone, UV radiation and climate interactions; (2) human health; (3) terrestrial ecosystems and biodiversity; (4) aquatic ecosystems; (5) biogeochemistry in a changing environment; (6) air quality; and (7) material damage (figure created by Emma Leslie, Global Challenges Program, Univ. of Wollongong, Australia)

The contribution of the Montreal Protocol to several of the United Nations Sustainable Development Goals (SDGs) is addressed in this EEAP 2020 Update Assessment. The SDGs and their targets are provided for a number of sustainability themes, including climate change, air and water quality, biodiversity and ecosystems, contaminants and materials, and human health (Fig. 1). Due to the Montreal Protocol, large increases in UV-B (280–315 nm) radiation have been avoided and global warming reduced through regulation of the ozone depleting substances, most of which are also potent greenhouse gases. The resulting changes in stratospheric ozone, ultraviolet (UV) radiation and climate are evaluated regarding the effects on humans and the environment. Some of the potential consequences are assessed of recent unexpected events, such as the COVID-19 pandemic (Sect. 9), and unprecedented increases in UV radiation over the Arctic in 2020 due to stratospheric ozone depletion (Sect. 2).

## Stratospheric ozone, UV radiation, and climate interactions

This section provides an update since our last assessments [1, 2] on recent findings of the interactions between stratospheric ozone, solar ultraviolet (UV) radiation and climate, and discusses the impact of the Montreal Protocol on these processes. In addition to protecting life on Earth from harmful UV radiation, the Montreal Protocol is also highly effective in mitigating global warming because most of the ozone-depleting substances (ODSs) regulated by the Montreal Protocol are also potent greenhouse gases (GHGs). According to new quantitative estimates, the implementation of the Montreal Protocol has prevented between a quarter and a third of the global mean increases in air temperature, depending on the time period considered. The stratospheric ozone hole over Antarctica continues to recover, with 2019 being one of the years with the lowest spring-time UV index measured at the South Pole since the start of measurements in 1991. In the Arctic, springtime episodes of stratospheric ozone depletion, identified first in the early 2010s, continue to occur. The last episode in the spring of 2020 led to the largest ozone loss measured to date and resulted in UV indices that were twice as high as typical at several Arctic locations. Additional topics discussed include: trends in measured surface UV radiation from ground-based and satellite data that are driven by changes in total ozone, aerosols and clouds; effects of Antarctic ozone depletion on the climate in the Southern Hemisphere and linkages to Australian wildfires; and projections of surface UV-B radiation for the second half of the twenty-first century. This section contributes to SDG 11.5, 12.4, 13.2, and 17.14 by providing further evidence that the Montreal Protocol has protected the climate and the stratospheric ozone layer.

### Additional evidence has accumulated that the Montreal Protocol is reducing global warming

Most ODSs controlled by the Montreal Protocol and its amendments are also potent GHGs with global warming potentials (GWPs) that are substantially larger than those of carbon dioxide (CO_2_) and methane (CH_4_). Over the second half of the twentieth century, ODSs were the second-most important GHG with approximately one third of the radiative forcing of CO_2_ [3]. The climate effects of ODSs were anticipated during the establishment of the Montreal Protocol [4], and their impact on climate has been continuously assessed since the ratification of the Montreal Protocol [5–7].

Building on earlier work [6, 8, 9], Goyal et al. [10] recently re-evaluated the amount of global warming that has been avoided due to the Montreal Protocol. This new study is based on a coupled atmosphere–ocean–land–sea–ice model and took into account the effect of the Montreal Protocol on emissions of ODSs that have contributed the most to stratospheric chlorine concentrations, namely the chlorofluorocarbons (CFCs) CFC-11 and CFC-12, as well as the CFC substitutes HCFC-22, HFC-125 and HFC-134a. Increases in GHG concentrations (including the concentrations of these ODSs) were described in this model by the Representative Concentration Pathway^1^ (RCP) [11] that leads to the strongest warming at Earth’s surface (RCP 8.5). The study concluded that, as of 2019, implementation of the Montreal Protocol has prevented warming ranging between 0.5 °C and 1.0 °C over large mid-latitude regions of land, particularly parts of Africa, North America and Eurasia. As much as 1.1 °C warming has been avoided over parts of the Arctic. In addition to quantifying benefits from the Montreal Protocol that have already been realised, Goyal et al. [10] also assessed the Montreal Protocol’s effect on the future climate for the RCP 8.5 scenario. Projected temperature increases that are likely to be averted by 2050 are on the order of 1.5 °C to 2 °C over most extrapolar land areas, and between 3 °C and 4 °C over the Arctic. Averaged over the globe (including the oceans), about 1 °C warming would be avoided by 2050, which corresponds to about 25% mitigation of global warming expected from all GHGs. The Montreal Protocol is contributing to SDG 13.2 by reducing global warming.

These benefits of the Montreal Protocol were corroborated by Polvani et al. [3] using calculations with a different climate model. When all known forcings (GHGs, ODSs) were taken into account, the simulated global-mean air temperature at the surface increased by 0.59 °C between 1955 and 2005. When the concentrations of ODSs and atmospheric ozone were fixed at 1955 levels, the resulting temperature change was only 0.39 °C. Hence, ODSs were responsible for about one-third of global warming over this period. Similar calculations for the Arctic (60–90° N) resulted in temperature changes of 1.59 °C and 0.82 °C for the two scenarios, respectively, suggesting that ODSs contributed about one-half to the warming at the Arctic surface. Changes in Arctic temperatures have a direct effect on sea ice loss. Polvani et al*.* [3] concluded that ODSs contributed one-half of the forced Arctic sea-ice loss in the latter half of the twentieth century (Sect. 5.5).

Like previous assessments [6, 8, 9], the studies by Goyal et al. [10] and Polvani et al. [3] took into account that ozone is also a GHG. Depletion of stratospheric ozone resulting from ODSs has therefore led to a small radiative cooling at the surface. However, both studies concluded that the magnitude of surface warming by ODSs far outweighs the cooling effect from ozone depletion. In a more recent multi-model study, Morgenstern et al. [12] estimated a larger cooling effect from ozone depletion than modelled by Goyal et al. [10] and Polvani et al*.* [3]. While these new calculations are consistent within error bars with previously published values [5], the larger cooling effect determined by Morgenstern et al*.* [12] means that the effect from phasing out ODSs would be smaller than summarised above. The effect of ODSs on climate is an area of active research and it is expected that refinements to climate chemistry models, e.g., by including compounding factors such as tropospheric ozone pollution, will further reduce uncertainties in estimating the effect of the Montreal Protocol on surface temperature.

In summary, these studies provide further evidence that the Montreal Protocol is not just vital for the recovery of the ozone layer, but also for the reduction of global warming. The Montreal Protocol has thus been the most successful international treaty to date to mitigate anthropogenic climate change resulting from the increase of GHGs.

The recently reported unexpected slowdown in the decline of the atmospheric concentration of CFC-11 after 2012 [13], which is partially caused by new emissions from eastern China [14], not only has the potential to delay the recovery of the ozone layer [15] but could also have a negative effect on future climate because the GWP of CFC-11 is 4660 times that of CO_2_ [5, 16]. So far, these unexpected CFC-11 emissions have not been large enough to significantly delay the closing of the ozone hole [17]. It is also unlikely that these emissions will have an important effect on global temperatures. However, the cumulative warming effect of ODSs, including carbon tetrachloride, CFCs-11, 12, 113, 113a, 114, and 115, Halons, HCFCs, HFCs, and N_2_O, is still significant [17]. Tightening the regulations concerning these substances by amending the Montreal Protocol could partially offset the effect of future CO_2_ emissions and reduce global warming.

### The stratospheric ozone hole over Antarctica continues to recover

Changes in the depth and extent of the Antarctic ozone hole have recently been analysed based on trends in four representative metrics describing the severity of Antarctic ozone depletion [18]. The four metrics are: the maximum 15-day averaged ozone hole area, the minimum 15-day averaged total ozone column (TOC), the integrated ozone deficit, and the duration of the ozone hole. After adjusting for the effect of stratospheric temperature on ozone depletion, trends in all four metrics over the 2001–2017 period are statistically significant (95% confidence level (CL)) and point in the direction of increasing ozone. These results are supported by Kramarova et al*.* [19] who calculated significant positive trends in TOC over a slightly longer period (1999–2019) for two metrics: the mean TOC for September averaged over the 60°–90° S latitude range (trend of 22.3 Dobson Units (DU) per decade, 94% CL) and the Antarctic minimum TOC (trend of 17.9 DU per decade, > 99% CL). These results confirm the conclusion from last assessments [7] that the Antarctic ozone hole is now in the process of recovery.

### The 2019 Antarctic ozone hole was the smallest on record and led to unusually small UV indices measured in Antarctica

Signs of the recovery of the stratospheric ozone layer over Antarctica (Sect. 2.2) are consistent with the decrease in the concentration of ODSs regulated by the Montreal Protocol. Assuming continued adherence to the Montreal Protocol, concentrations of ODSs are projected to decline further, eventually resulting in the disappearance of the annually recurring ozone hole in the second half of the twenty-first century [7]. Until that time, large year-to-year variations in the various ozone hole metrics are expected because of the sensitivity of chemical ozone destruction to temperatures in the lower stratosphere in the presence of ODSs as explained below.

Record-high temperatures in the stratospheric polar vortex over Antarctica during September and October 2019 led to the smallest Antarctic ozone hole recorded since the early 1980s [19]. Averaged over the polar cap (60°–90° S), the average TOC in that period was the highest over the last 40 years, and the minimum TOC for September 2019 was the highest observed since 1988. For the months of September, October, and November, the polar cap average TOC was higher by 29%, 28%, and 26%, respectively, compared to the mean of the 2008–2018 period [20].

The weak ozone hole was caused by abnormally strong planetary wave activity originating in the subtropical Pacific Ocean east of Australia and over the eastern South Pacific [19, 21, 22]. These waves weakened the stratospheric polar vortex, which led to a warming of the polar stratosphere, starting in mid-August [23]. The resulting above-normal temperature in the lower stratosphere reduced the occurrence of polar stratospheric clouds (PSCs), which provide surfaces for heterogeneous chemical reactions involving chlorine that destroy ozone catalytically. The volume of PSCs dropped to almost zero by mid-September and the chemical processes leading to ozone depletion were therefore suppressed far earlier than usual.

**Fig. 2:**
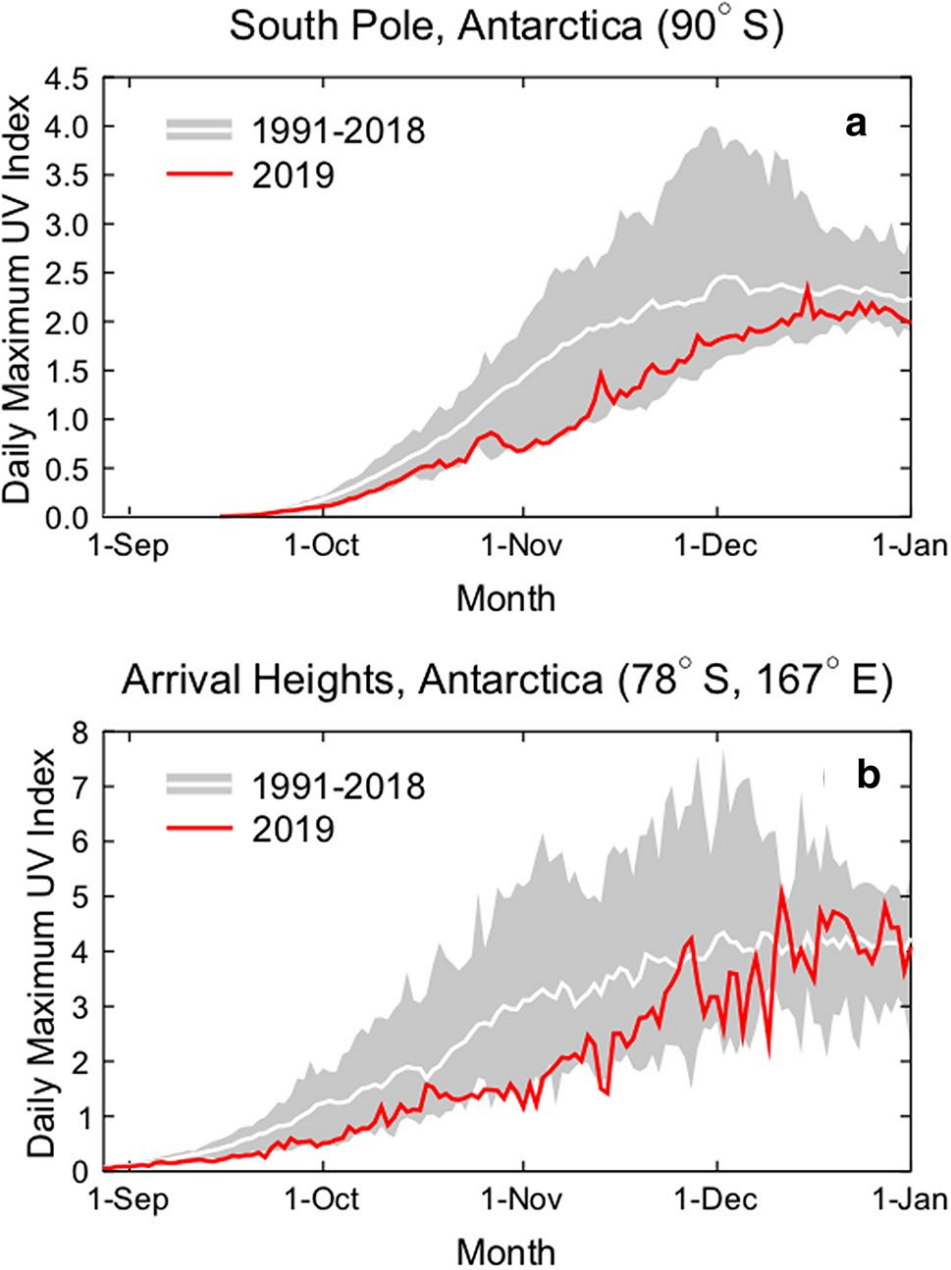
Daily maximum UV indices measured at the South Pole (**a**) and Arrival Heights (**b**) in 2019 (red line) compared with the average (white line) and the range (grey shading) of daily maximum observations of the years 1991 to 2018. The UV indices were calculated from spectra measured by SUV-100 spectroradiometers. Up to 2009, the instruments were part of the NSF UV monitoring network [25] and they are now a node in the NOAA Antarctic UV Monitoring Network (https://www.esrl.noaa.gov/gmd/grad/antuv/). Consistent data processing methods were applied for all years [26, 27]

The record-high TOC observed over Antarctica during the spring of 2019 led to unusually low UV indices (a commonly used measure to quantify the portion of the UV spectrum that causes sunburn) measured at the South Pole (90° S) and Arrival Heights (78° S), a research station overlooking McMurdo Sound. Figure 2 shows the measured UV index at the two sites for 1 September through 31 December 2019 compared with the mean and range calculated from measurements of the years 1991–2018. Between October and mid-November 2019, the UV index at the South Pole was at the minimum of the historical (1991–2018) range, and remained close to this minimum between mid-November and January. At Arrival Heights, the UV index in 2019 was close to the minimum between September and mid-November, and stayed below the long-term mean until mid-December, with the exception of two short periods. Similarly, reduced UV indices compared to the observational record, which began in 2007 [24], were observed from September to December at the Australian Antarctic research stations of Casey (66° S), Mawson (68° S) and Davis (69° S). Data from each of these sites confirm that UV-B radiation in Antarctica is mainly controlled by TOC, in contrast to sites at lower latitudes where the effects of clouds and aerosols are dominant (Sect. 2.7).

### New evidence confirms a strong link between Antarctic stratospheric ozone depletion and the climate of the Southern Hemisphere

Stratospheric ozone depletion over Antarctica has led to changes in the summertime tropospheric circulation in the Southern Hemisphere since the latter decades of the twentieth century. These changes have included the poleward shift in the belt of winds at mid-latitudes, a trend towards lower surface pressure over Antarctica compared with mid-latitudes (i.e., a more positive phase of the Southern Annular Mode or SAM^2^) and concomitant changes in temperature and precipitation patterns [28–36]. Recent work has further highlighted these links, and has shown evidence of changes due to stratospheric ozone recovery.

Banerjee et al*.* [37] used meteorological reanalyses and simulations with a single climate model to show that circulation changes induced by stratospheric ozone loss in the 1980s and 1990s halted or partly reversed around the end of the twentieth century. Furthermore, they find that these changes are primarily the result of the reversal in the severity of Antarctic ozone depletion due to actions prompted by the Montreal Protocol to decrease emissions of ODSs. Hence, the Montreal Protocol has contributed to SDG 11.5 by returning circulation patterns to their natural state.

Using observations and single-model climate simulations, Damiani et al*.* [38] found that the link between Antarctic ozone loss in spring and changes in annual precipitation has strengthened in the last few decades. While they point out that the amount of Antarctic ozone loss in any given year relates to the strength of downward coupling from the stratosphere to the surface, they find that the increase in stratospheric ozone depletion led to a tendency towards drier conditions in Chile and more precipitation in parts of Australia, and to a lesser extent in South America. Hence, springtime ozone anomalies in Antarctica can be used as a predictor of subsequent summer precipitation for South America and Australia. A similar link between ozone anomalies and precipitation has also been established for Antarctica [34].

### The early break-up of the stratospheric polar vortex over Antarctica in 2019 has likely exacerbated the Australian wildfires of 2019/2020, but the contribution of ozone depletion is still unknown

According to a recent study, weakening and warming of the stratospheric polar vortex over Antarctica substantially increased the likelihood of hot and dry weather extremes across subtropical eastern Australia from austral spring to early summer [39]. The unusual warming of the Antarctic stratosphere in September 2019 (Sect. 2.3) may have exacerbated the extremely dry conditions observed during the summer of 2019/20 in the Southern Hemisphere [23, 40–44], leading to devastating wildfires in Australia [45]. Specifically, the stratospheric warming event influenced the troposphere from mid-October 2019 by forcing the SAM from a positive phase to a negative phase, which enhanced anomalously hot and dry conditions in eastern Australia [44]. We emphasise that the stratospheric warming event, the change in the phase of the SAM, and the small ozone hole in the spring of 2019 are all attributable to unusually strong planetary-scale wave activity in 2019. There have been no specific studies for this event addressing the question of whether the presence of ODSs in the atmosphere contributed to the properties of these waves or influenced the coupling between the stratosphere and troposphere. Hence, the role of the Montreal Protocol in this extreme weather event is still undetermined.

The fires that occurred in eastern Australia in December 2019 and January in 2020 affected over 10 million hectares and caused unprecedented atmospheric effects throughout the Southern Hemisphere [46–49]. Superheated air from the fires produced large-scale pyrocumulonimbus clouds, forcing smoke into the lower stratosphere, from where it rose to heights up to 35 km [49]. The rising plumes carried ozone-poor tropospheric air into the stratosphere, which produced localised depletion of ozone, with the TOC reduced by up to 100 DU or in excess of one third of the typical TOC [46, 49]. The rising air also locally increased the water vapour mixing ratio in the lower stratosphere at southern mid-latitudes [49] where it might be expected to deplete ozone through enhanced heterogeneous reactions [50]. The smoke also increased aerosol absorption in isolated areas [48, 49]. It can be anticipated that the plume-induced ozone dilution and depletion increased UV radiation at the surface while smoke and aerosols resulting from the fires led to decreased UV radiation. However, the combined effects of these two opposing mechanisms on UV radiation in the affected areas are not yet known, as measurements of UV radiation from these areas have not yet been reported.

Using future projections of the 6th Coupled Model Intercomparison Project (CMIP6) for a range of emissions scenarios, Bracegirdle et al*.* [51] have updated earlier assessments (e.g., [52]) of changes in surface temperature, precipitation and the zonal wind speed over Antarctica and the Southern Ocean. In the first half of the twenty-first century, stratospheric ozone recovery will shift the westerly jet equatorward, but this shift will be partially cancelled depending on the GHG scenario (defined by Shared Socio-economic Pathways (SSP^3^) [53]) that eventuates: for low emissions scenarios (SSP1-2.6 and SSP2-4.5) there is no significant change, but for high emissions scenarios (e.g., SSP5-8.5), there is a tendency for further poleward progression and strengthening of the jet. During the second half of the twenty-first century, ozone forcing is expected to weaken while forcing by GHG will strengthen, resulting in a general southward shift of the westerly jet for all scenarios. Temperature and precipitation patterns over the Southern Ocean will regionally respond to the jet location, but for the interior of Antarctica, changes will be dominated by increases in GHG irrespective of the location of the jet.

### Unprecedented increases in Arctic solar ultraviolet radiation were caused by exceptionally large stratospheric ozone depletion in 2020

Total ozone columns averaged over the northern polar cap (63°–90° N) were exceptionally low in late winter and early spring (February–April) of 2020 [54]. The average TOC in 2020 for this 3-month period was 340 DU, which is 100 DU below the mean of measurements between 1979 and 2019 and the lowest value since the start of satellite measurements in 1979. The low TOCs in 2020 were partially caused by a strong and long-lived polar vortex, which provided ideal conditions for chemical ozone destruction to take place. Temperatures low enough to form PSCs within the vortex developed early in the season, and on average enclosed about a third of the vortex volume [54–58]. These conditions are unique in the ~ 40 years of measurements, making 2020 the year with the largest Arctic ozone loss on record. The occurrence of a small ozone hole with little ozone depletion over Antarctica (Sect. 2.3) and large ozone depletion over the Arctic within a 6-month period is a coincidence and cannot be attributed to a common cause.

The low TOC led to record-breaking anomalies in solar UV-B radiation over the Arctic measured by ground-based instruments at ten Arctic and subarctic locations and observed by the Ozone Monitoring Instrument (OMI) on NASA’s Aura satellite [59]. UV radiation anomalies were particularly large between early March and mid-April 2020. The UV indices measured in 2020 exceeded the historical (2005–2019) mean by up to 100%. At several locations in northern Canada and Scandinavia, historical means were surpassed by more than six standard deviations. However, absolute anomalies remained below 3 UV index units because the enhancements occurred during a period when the solar elevation at these Arctic locations is small.

### New studies reported trends in solar UV radiation for many mid-latitude regions, confirming that changes in solar UV radiation during the last 20 years have generally been small and mostly caused by changes in cloud cover

Trends in UV radiation were calculated from UV measurements for several European stations (Reading (51° N), Uccle (51° N), Thessaloniki (41° N), and Sodankylä (67° N)) [60]; Northern Eurasia (a region north of 40° N extending from Scandinavia to Siberia) [61]; and a site near the equator (Quito, Ecuador, 2,850 m above sea level) [62]. These new studies generally confirmed the conclusions from our last update assessment [1], i.e., changes in UV radiation during the last 20 years have been small (generally less than *ca*. 4% per decade) because the Montreal Protocol has prevented large decreases in TOC. For most locations outside the polar regions, long-term changes in UV-B radiation are mainly governed by variations in clouds, aerosols, and surface reflectivity, while changes in TOC are less important.

Results from these ground-based measurements are corroborated by satellite data. Using observations from OMI, Herman et al*.* [63] showed that noon-time erythemal irradiance at 191 cities located between 60° N and 60° S has not significantly (95% CL) changed over the period 2005–2018. However, when data are averaged over 15° latitude bands, there are strong correlations between erythemal UV radiation and short- and long-term changes in clouds and absorbing aerosols, as well as inverse correlations with TOC. The largest changes in erythemal irradiance between 60° N and 60° S are caused by changes in cloud and aerosol transmission, and range between − 4 and 4% per decade.

In contrast to the situation in the tropics and the mid-latitudes, long-term changes of UV radiation in Antarctica continue to be dominated by changes in TOC. A new study [27] updated an earlier [64] analysis of trends in the UV index at three Antarctic sites: South Pole (90° S), Arrival Heights (78° S), and Palmer Station (65° S). At the South Pole (a site representative of the Antarctic Polar Plateau), significant (95% CL) decadal trends of − 3.9% and − 3.1% over the period 1996–2018 were calculated for January and February, respectively, which can mostly be explained by concomitant positive trends in TOC. At Arrival Heights, a significant (90% CL) trend in the UV index of − 3.3% per decade was calculated for summer and was attributed to a significant upward trend in TOC of 1.5% per decade for January plus the effect of changes in fast ice (i.e., sea ice fixed in place by land, grounded icebergs, or ice shelves) covering the sea adjacent to the instrument site. Thus, this study provides evidence that the UV index in Antarctica is starting to decrease during summer months. However, statistically significant reductions for spring (October and November), when the ozone hole leads to large UV index variability, were not detected. Trends for spring were also not apparent even when data from 2019—the year when an unusually small ozone hole was observed (Sect. 2.3)—were included.

### UV-B radiation at low and mid-latitudes is projected to increase in the second half of the twenty-first century due to less cloud cover resulting from increasing greenhouse gases

Simulations with a chemistry climate model (EMAC) for the period 1960–2100 were used to derive trends in DNA-damaging radiation at four mid-latitude locations and one tropical high-altitude site [65]. DNA-damaging irradiance averaged over the five locations is projected to increase by 1.3% per decade between 2050 and 2100. To isolate the effect of GHGs on climate, one simulation assumed increasing GHGs according to RCP 6.0 and the second adopted constant GHGs at 1960 levels. No trend in total ozone was detected by the model after 2050, and the trend in DNA-damaging irradiance was attributed to a statistically significant (95% CL) decrease in cloud cover of − 1.4 per decade resulting from increasing GHGs. The study suggests that changes in UV-B irradiance at low- and mid-latitudes during the second half of the twenty-first century will be dominated by factors other than changes in stratospheric ozone [65]. However, these projections depend on the accurate description of clouds by climate models. Uncertainties in the modelling of clouds propagate to the projected changes in solar UV-B radiation.

### Measurements of UV radiation from space with a new instrument have been validated and will continue data records started by legacy instruments

Most estimates of the surface UV radiation from space have historically been based on NASA’s total ozone mapping spectrometers (TOMS), which are available up to 2006, and OMI (for data, see [66] and https://acdisc.gesdisc.eosdis.nasa.gov/data/). OMI measurements on the Aura satellite started in 2004 [67] and will likely be discontinued in a few years [1]. The TROPOspheric Monitoring Instrument (TROPOMI) on the European Space Agency’s Sentinel-5 Precursor satellite will provide space-based estimates of UV radiation for the future. TROPOMI observations of UV radiation have recently been compared with ground-based measurements at 25 sites [68]. For snow-free surface conditions, the median relative difference between measurements of the UV index by TROPOMI and these ground stations agreed within ± 10% and ± 5% at 18 and 10 sites, respectively. These differences are comparable to those reported for OMI [69, 70]. Larger differences were observed at locations with challenging conditions, such as mountainous areas or sites in the Arctic and Antarctic with variable snow cover. TROPOMI and OMI data agree with ground-based measurements within a similar range. A comprehensive comparison between OMI and TROPOMI surface UV products is planned [67] to ensure that there is no step-change in the time series of UV radiation measurements when transitioning from OMI to TROPOMI.

## Human health

By mitigating increases in UV-B radiation reaching Earth’s surface, the Montreal Protocol has influenced human health, both directly and indirectly. Models estimate that a large number of skin cancers and cataracts have been avoided. Concern about ozone, and the international focus on methods to mitigate its loss, are also likely to have contributed to increased investment in sunscreen technology and promotion of strategies to reduce the harmful effects of over-exposure to the sun (SDG 13.3). However, it is important to note that sun exposure also has benefits for human health, and to date the effects of the Montreal Protocol on these are unclear. Below we describe new information about trends in skin cancer and the effects of exposure to UV radiation on human health; these should be considered in future modelling evaluations of the effects of the Montreal Protocol on human health.

### A new model has estimated the number of skin cancers and cataracts avoided due to implementation of the Montreal Protocol

Estimates generated by the United States Environmental Protection Agency using the updated Atmospheric and Health Effects Framework (AHEF) model indicate that full implementation of the Montreal Protocol is expected to prevent 432 million cases of keratinocyte cancer and 11 million cases of melanoma in the United States for people born in the years 1890–2100 [71]; it is also expected to prevent 2.3 million deaths from skin cancer (predominantly melanoma, but also cutaneous squamous cell carcinoma (SCC)), and 63 million cases of cataract. These numbers are greater than the previous 2015 estimates (328 million keratinocyte cancer cases; 10 million melanoma cases; 1.8 million skin cancer deaths; 33 million cataract cases) due to updated physico-chemical parameters, improved UV irradiance calculations, and updated population data.

The AHEF health effects model estimates the change in skin cancer incidence that would occur as a result of a relative change in the dose of UV radiation under different scenarios of emission of ozone-depleting substances, while assuming that sun-protection behaviours remain constant. The largest effects on incidence of skin cancer are estimated to occur in people born between 1960 and 1980 since these birth cohorts experienced the full period of stratospheric ozone depletion, and will also have received the largest cumulative lifetime dose of UV radiation. The model estimates that cohorts born in 2040 or later will not experience any excess incidence of skin cancer caused by the effects of ozone depletion.

### New melanoma incidence data show that temporal trends differ by country, age, sex and anatomic site

In the United States, the incidence of cutaneous malignant melanoma increased in adults of all ethnicities aged 40 years or older between 2001 and 2015 at an average annual percent change (AAPC) of 1.8% [72]. In non-Hispanic whites, the increase was more marked between 2001 and 2005 (AAPC 3.9%) than in the most recent time period, suggesting some plateauing of incidence rates (2005–2015, AAPC 1.7%) [73]. Significant declines in incidence in adolescents and young adults were observed for the period 2001 to 2015 (AAPC ranging from − 3.6% to − 5.4%) [72]. Compared with adults born around 1956, those born around 1991 had a 15% lower risk of melanoma [incidence rate ratio (IRR) 0.85, 95% CI 0.77–0.94] [73]. These patterns may be attributable to public health programs and/or broader societal changes, such as increasing indoor recreational activities facilitated by the advent of small personal computing devices.

An analysis of National Cancer Registry data from South Africa from 2005 to 2013 found an overall age-standardised incidence of melanoma (world standard population) of 2.7 per 100,000 people per year, but there was a marked difference between white (23.2 per 100,000) and black (0.5 per 100,000) populations [74]. In China, the incidence of melanoma increased from 1990 to 2017, with the greatest increase from 2006 to 2017 (AAPC 6.1%, 95% CI 5.6–6.6%) [75].

**Fig. 3:**
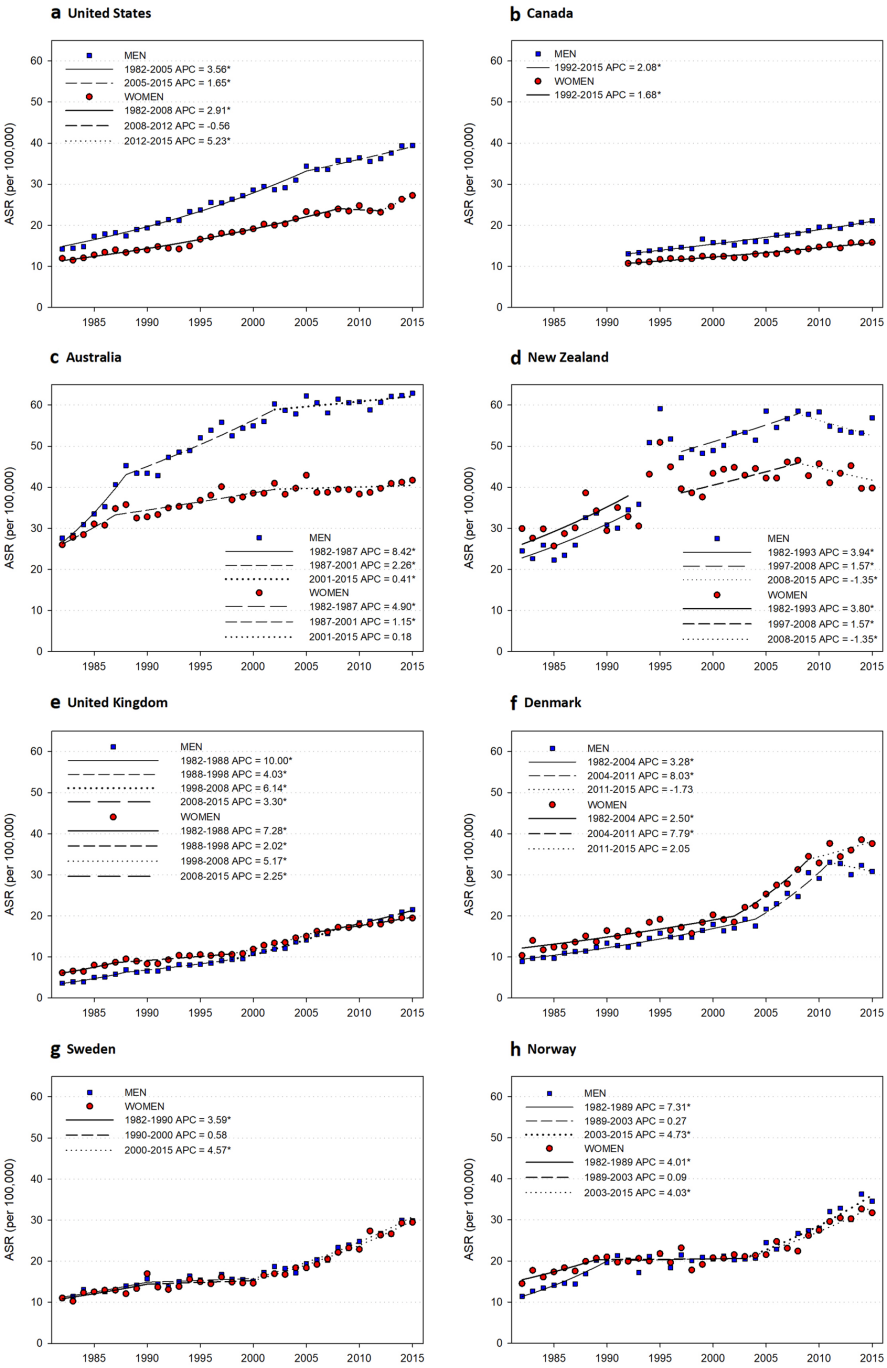
Age-standardised melanoma incidence from 1982 through 2015 and annual percentage change in eight populations: **a** United States whites; **b** Canada; **c** Australia; **d** New Zealand; **e** United Kingdom; **f** Denmark; **g** Sweden; and **f** Norway. *APC* annual percentage change; *ASR* age-standardised rate (US 2000). *The APC is significantly different from zero at *α* = 0.05. Reproduced with permission from [76]

Data on the incidence of melanoma according to sex and body site for the period 1982–2015 for Australia, New Zealand, Canada, the United States, the United Kingdom, Norway, Sweden, and Denmark show that in the most recent decade (2005 to 2015) incidence increased for all countries except New Zealand and Denmark (Fig. 3) [76]. Total melanoma incidence was higher in men than women in ‘new world’ populations (those more recently populated by people of European descent; United States whites, Canada, Australia, and New Zealand) but not in ‘old world’ countries (United Kingdom, Norway, Sweden, and Denmark) [76]. The male to female incidence rate ratio varied with age. In all populations cutaneous melanomas were more common in women than in men in those aged < 45 years, mostly due to higher rates of melanoma on the lower limb in women. The opposite was true for people aged ≥ 70 years due to the higher incidence on the head and neck in men. These sex- and site-specific patterns most likely indicate combinations of sex differences in the anatomic distribution of naevi [77] and patterns of sun exposure. These differences may need to be considered in models to predict future melanoma risk.

### Trends in melanoma mortality differ by sex in most countries

**Fig. 4:**
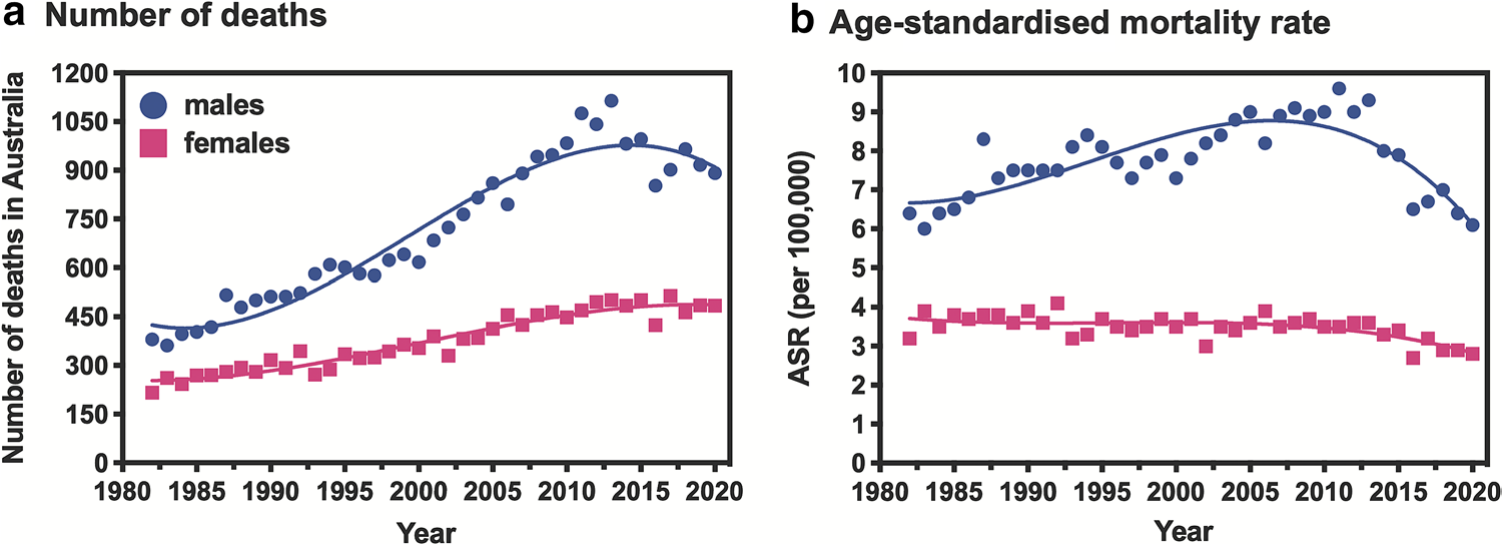
Trends in melanoma mortality in Australia from 1982 to 2018 with projections to 2020. **a** number of deaths; **b** age-standardised mortality rate. Data from the Australian Institute of Health and Welfare National Mortality Database [80]. Figure produced by S. Byrne

Trends in mortality from melanoma are driven by trends in incidence and screening practices, and by improved treatments. New information on melanoma mortality was reported for 31 countries, using data from the WHO Mortality Database, covering the time period from 1985 to 2015. An overall increase in mortality was reported for men in all countries, in contrast with stable or declining rates in women [78]. For the most recent time period (2013–2015), the median mortality rate was 2.6 deaths per 100,000 for men and 1.55 per 100,000 for women; the highest mortality rates were recorded for Australia and Norway for men, and Norway and Slovenia for women. The increase in most countries reflected increasing mortality rates in people aged 50 years or older; mortality rates were generally stable or declining in younger age groups. A separate report for Spain over the period 1982–2016 showed a similar trend, with mortality rates stabilising in men and women younger than 64 years from the mid-90s, while rates continued to rise in older age groups [79]. An analysis of data from the Australian Institute of Health and Welfare [80] suggests that in Australia the mortality rates in men and women are declining, although this appears much more marked in men (Fig. 4).

Improved treatments for metastatic melanoma over the past decade are partly responsible for the declining mortality. Data from the United States (restricted to whites) have shown that the introduction of new systemic therapies was associated with a significant reduction in the melanoma mortality rate over the period 2013–2016 (AAPC − 6.2, 95% CI − 8.7 to − 3.7) [81].

### In some European nations the incidence of keratinocyte cancers and pre-malignant cancers is continuing to increase, with differences according to sex and the anatomic site of the tumour

A whole-of-population study in Iceland, based on registry data, reported that from 1981 to 2017 the incidence of basal cell carcinoma (BCC) increased 2.3-fold in men and 3.7-fold in women [82]. As a consequence of the greater increase in women, the incidence in women was 1.4 times higher than in men in the final 5 years of the study period (age-standardised incidence rates 83.1 and 59.9 tumours per 100,000, respectively), whereas at the beginning of the period, it was marginally lower in women (22.2/100,000) than in men (25.7/100,000). The lifetime risk increased from 3.2 to 10.1% in women and from 2.8% to 7.3% in men. The increase in incidence was highest for BCC on the trunk and legs; between 1981 and 1990 72% of BCCs were located on the head and neck in both men and women, whereas in 2009–2017 period this percentage had decreased to 57% for men and 49% for women. Given the observed incidence patterns, and the very low ambient UV radiation in Iceland, it is most likely that these changes are due to increased use of tanning beds and/or more frequent travel to sunny locations.

In the Netherlands, a study of SCC in situ found a marked increase in incidence between 1989 and 2017 that was more pronounced in women than in men [83]. In men, there was a sixfold increase in the age-standardised rate (European standard population) from 11.1 cases to 67.8 cases per 100,000 person years; in women there was a 7.7-fold increase from 9.3 to 71.7 per 100,000 person years. In men, the increase was greatest for lesions on the scalp and neck, whereas in women it was greatest for lesions on the trunk. The differences in behaviour that underlie these patterns emphasise the importance of considering sex differences when modelling the potential effects of the Montreal Protocol on human health.

### Skin cancer treatment is costly, and primary prevention is cost-effective

The introduction of new and costly systemic treatments for melanoma, combined with increased incidence of all skin cancer types, has resulted in a markedly increased economic burden of skin cancer in the Netherlands [84]. In 2017, skin cancer was the fourth most costly cancer to manage (€465 million). The total cost was 1.7 times higher than in 2007 (€278 million), and over this decade the cost of drugs increased from €0.7 million to €121 million because of the advent of costly immunotherapy used to treat advanced melanoma. By 2030 the costs are projected to reach €1.35 billion.

In Queensland (Australia), an area with very high ambient UV radiation [85], modelling showed that compared with annual clinical skin examinations (early detection), and no intervention, daily sunscreen use (prevention) resulted in fewer cases of melanoma and keratinocyte cancer, significantly lower costs associated with diagnosis and treatment, and small differences in life years saved (0.9%) and quality-adjusted life years gained (0.10%). While these findings may not generalise to settings with lower ambient UV radiation, they emphasise the value of increasing primary prevention behaviours in populations with high incidence of skin cancer.

### Commonly used drugs remain a cause of photodermatoses, and may increase skin cancer risk

Approximately 5% of patients with photodermatosis who were referred to a photobiology unit had photosensitivity resulting from oral medication. While UV-A is the main contributing waveband, detailed in vivo assessment using monochromatic testing with wavelengths between 300 and 600 nm shows that exposure to UV-B radiation also contributes in a proportion of cases (14.5%) [86]. Many common medications cause acute photosensitivity, including thiazide diuretics, quinine, antibiotics, antidepressants, and anti-epileptics. New photosensitising drugs are emerging, including proton-pump inhibitors and statins [86]. An analysis of drugs dispensed between 2010 and 2017 in Germany and Austria found that of > 632 million (Germany) and > 113 million (Austria) drugs dispensed, almost 50% had photosensitising potential [87].

The risk that photosensitising drugs could induce skin cancer may represent a significant public health issue. The commonly prescribed diuretic, hydrochlorothiazide, has emerged as a culprit in recent studies, leading the European Medicines Agency Pharmacovigilance Risk Assessment Committee to recommend that the product information be updated to include advice about the increased risk of keratinocyte cancer with hydrochlorothiazide use in Europe [88].

### Eye disorders related to sun exposure continue to impose a considerable burden of disease

Exposing the eyes to UV radiation increases the risk of cataract, which remains the leading cause of vision loss world-wide. In 2015, cataract accounted for 35% of total blindness, with projections suggesting that this remains the case in 2020 [89–94]. Globally, the age-standardised DALY rate increased by 10% from 1990 to 2016 (to 88.3/100,000), although there was no increase in the last decade of this period [95].

In a population-based study of 9735 adults aged over 40 years in India, 33% were found to have cataract, with little difference between men and women. There was a strong association with lifetime effective sun exposure; the odds of cataract for those in the highest quintile was 9.4 times that in the lowest quintile (95% CI 7.9–11.2) [96].

### New studies suggest a limited role for vitamin D in health, apart from musculoskeletal conditions

Production of vitamin D is the best known benefit of exposing the skin to UV-B radiation. Vitamin D plays a key role in maintaining musculoskeletal health. There is controversy about the optimal concentration of the metabolite measured to assess vitamin D status (25 hydroxy vitamin D [25(OH)D]); it is generally accepted that 25(OH)D < 25–30 nmol/L increases the risk of musculoskeletal disorders, but many organisations advise maintaining levels of at least 50 nmol/L to minimise the risk of falls and fractures. Observational studies suggest that there may be links with many other health outcomes and, if this is the case, a higher 25(OH)D concentration may be warranted. However, the observed associations may not be causal, and randomised controlled trials have mostly failed to identify benefits of vitamin D supplementation. Mendelian randomisation studies, in which genetically predicted, rather than measured, 25(OH)D concentration is used to test a link with health outcomes can overcome some of the biases inherent in observational studies. Several recently published Mendelian randomisation studies have failed to identify links between vitamin D and cardiovascular/metabolic diseases, depression, non-vertebral fracture, all-cause mortality [97, 98], or cognitive and psychiatric traits [99].

These Mendelian randomisation analyses do not account for non-linear effects so cannot exclude a possible effect of very low 25(OH)D concentration, but they suggest a limited benefit of treatment to increase 25(OH)D concentrations in people who are not markedly deficient. These findings are important as they underpin models aiming to identify the amount of UV-B radiation needed to avoid vitamin D deficiency.

### Vitamin D deficiency appears to be widespread on the African continent and in people of South-Asian origin living in the United Kingdom

A meta-analysis of vitamin D deficiency on the African continent found that 18.5% and 34.2% of study participants had a serum 25(OH)D concentration of < 30 nmol/L and < 50 nmol/L, respectively [100]. However, the heterogeneity between studies was very high and could not be explained by age group, geographical region, residence in a rural *vs* urban region, vitamin D assay, or risk of bias. While this study suggests that there is a moderately high prevalence of vitamin D deficiency in Africa, population-based studies using consistent methods (including vitamin D assays) are needed to enable inter-country comparisons and inform sun exposure and nutrition policies.

An analysis of blood samples from 6433 adults (aged 40–69 years) of South-Asian descent from the UK Biobank cohort (samples collected between 2006 and 2010) found a very high prevalence of vitamin D deficiency; 92% had 25(OH)D concentration < 50 nmol/L, 55% were < 25 nmol/L and 20% had very severe deficiency (25(OH)D < 15 nmol/L). When an additional 843 participants with undetectable 25(OH)D concentration were included the prevalence of severe vitamin D deficiency rose to 29%. While these data are now at least a decade old, and new recommendations that residents of the United Kingdom should routinely take a 400 international unit supplement may have reduced the prevalence of vitamin D deficiency, they highlight the high risk borne by dark-skinned people living at high latitudes [101]. One of the main consequences of vitamin D deficiency in children is rickets, and over 80% of children with rickets in the United Kingdom are of South Asian or black ethnicity [102].

### UV radiation-induced modulation of the immune system has both beneficial and adverse effects

**Fig. 5:**
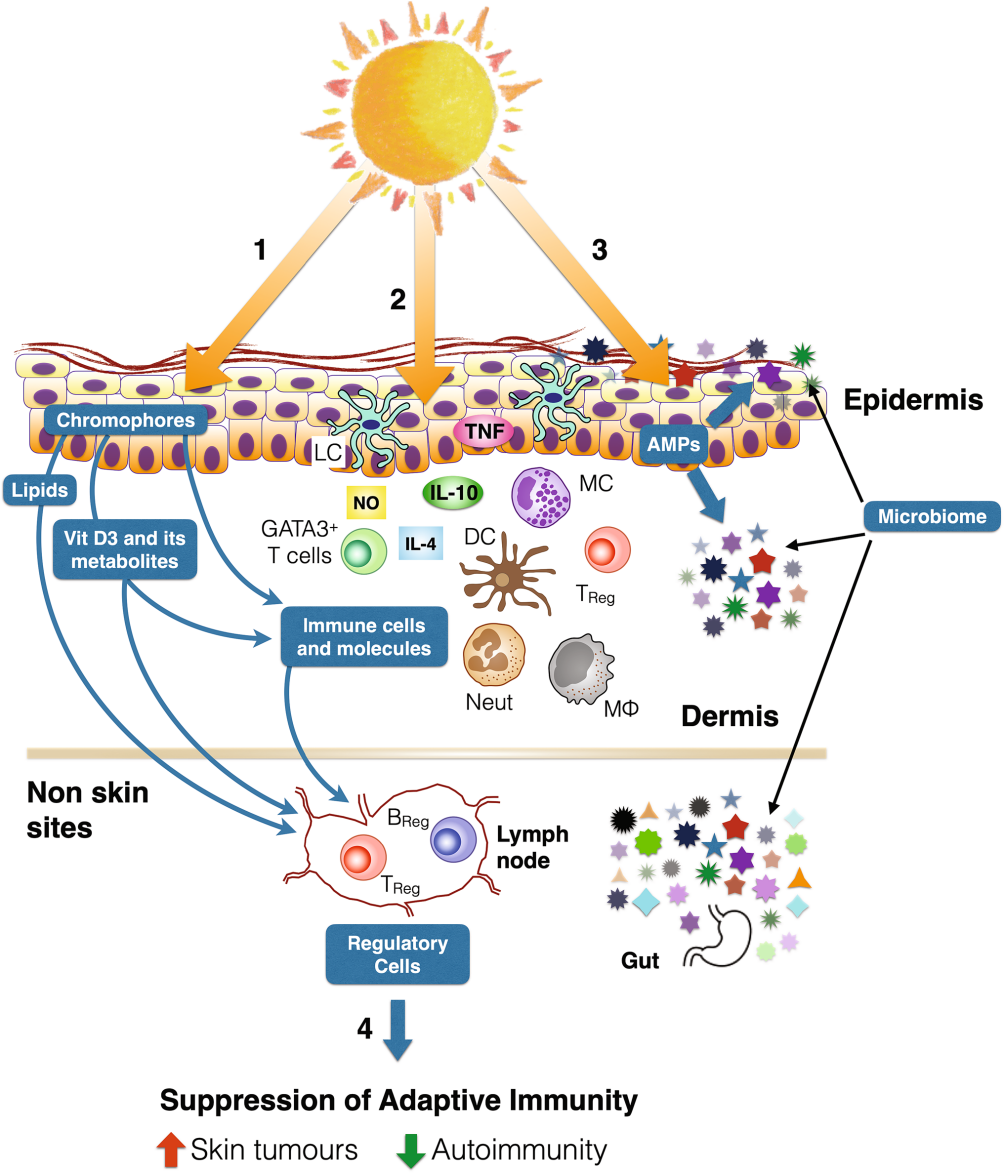
Exposing the skin to UV radiation affects the local and systemic immune system. (**1**) UV is absorbed by skin chromophores including DNA, urocanic acid, and tryptophan metabolites. UV radiation-induced changes in cutaneous lipids can also affect the local and systemic immune systems. (**2**) Epidermal keratinocytes and Langerhans cells (LC), as well as dermal dendritic cells (DC) and mast cells (MC), respond to nitric oxide (NO) and UV radiation by releasing immune-modulatory cytokines including tumour necrosis factor (TNF) and interleukin 10 (IL-10). These events lead to the recruitment from blood of innate immune cells including IL-4-producing neutrophils (neut) and macrophages (MΦ). This recruitment and activation of the innate immune system is reinforced by (**3**) local production of vitamin D_3_ and its metabolites which, together with UV radiation, can induce the production of antimicrobial peptides (AMPs). These events are likely to influence the cutaneous microbiome which may increase skin irritation and rashes, and also alter immune responses to pathogens. Exposure to UV radiation also increases the diversity of the gut microbiome which has potential benefits for health. (**4**) In response to high and prolonged doses of UV radiation, regulatory T and B cells (T_Reg_ and B_Reg_, respectively) are activated in lymph nodes that drain from local skin. The subsequent suppression of adaptive immune responses increases the risk of skin cancer but may explain why exposure to UV radiation may reduce the risk of autoimmune disease such as multiple sclerosis. Figure designed by S. Byrne

Exposing the skin to UV radiation affects the local and systemic immune system through both vitamin D and non-vitamin D pathways. Depending on the wavelength, dose, and frequency of exposure to UV radiation, adaptive (i.e., acquired) immune responses are often suppressed [103, 104]. Exposure to UV radiation induces alterations to cutaneous cells, molecules [105], the transcriptome [106], and commensal bacteria [107], leading to the downstream activation of regulatory T and B cells. These exert their suppressive effects for sustained periods of time, both locally at the site of exposure to UV radiation and distantly at un-irradiated sites (Fig. 5) [108]. Other immune cells, including recently described GATA3+ T cells [109], may also be involved. Suppression of the cutaneous immune response is an important contributor to the pathogenesis of skin cancer [104]. This is particularly the case when the amount of UV radiation received is high (i.e., sunburning) and/or prolonged (i.e., chronic). The distant effects of UV radiation on the immune response may drive melanoma development at non-skin sites such as the eye [110].

In contrast to suppression of adaptive immunity, innate immune responses, particularly at the site of exposure to UV radiation, are activated (Fig. 5). While this activation may lead to protection from localised infection, it is also likely to affect the cutaneous microbiome (the community of micro-organisms present in and on the skin), albeit with a high degree of variability between, and even within (different skin locations) people [111]. In general, the changes to the cutaneous microbiome induced by exposure to UV radiation appear to increase skin irritation and rashes, and also alter immune responses to pathogens. New research highlights implications of this immune modulation induced by exposure to UV radiation for some diseases as described below.

Herpes zoster (shingles) is a painful blistering skin condition that can be followed by disabling neuralgia. It results from reactivation of latent varicella zoster virus (VZV). In 205,756 participants from three prospective United States cohort studies followed for 16–24 years, over 24,000 incidents of herpes zoster were reported [112]. Higher estimated exposure to UV radiation was associated with a 14% higher risk for herpes zoster in men (adjusted hazard ratio 1.14; 95% CI 1.02–1.2), but there was no association in women. This is the first longitudinal study reporting increased risk of VZV reactivation related to exposure to UV radiation. These findings require verifying, but may be explained by suppression of skin immunity [113].

There has been additional evidence that exposure to UV radiation reduces the risk of some autoimmune diseases. In the United States Radiologic Technicians Study, there was a linear association between lower winter (but not summer) lifetime average ambient UV radiation dose at the location of residence and increased risk of developing multiple sclerosis (MS; adjusted HR = 2.00, 95% CI 1.21–3.30 for lowest *vs* highest of four categories) [114]. A recent meta-analysis confirms a latitude gradient in the prevalence of MS which may be growing stronger; this provides further evidence of the importance of environmental factors in disease pathogenesis [115]. In children, higher sun exposure was associated with a lower risk of developing inflammatory bowel disease in an Australian study (adjusted OR 0.94, *P* = 0.002 for each additional 10 min outdoors) [116].

### Exposure to UV radiation has potential effects on cancer and metabolic disease

The gut microbiome is emerging as a key player in multiple health outcomes, including some cancers, and metabolic, autoimmune, and psychiatric disorders [117]. Exposing the skin to UV radiation can increase the diversity of the gut microbiome, with the potential for a positive impact on disease [118]. It is not yet clear whether this is through vitamin D-dependent or independent pathways [119], but a systematic review of in vivo studies found suggestive links between vitamin D and the microbiome in both mouse and human studies [120].

In a systematic review and meta-analysis, greater time spent outdoors was associated with reduced risk of breast cancer (≥ 1 h/day in the sun during summer months over a lifetime or usual adulthood compared with < 1 h/day: pooled relative risk = 0.84, 95% CI 0.77–0.91) [121], with some evidence of a stronger effect for exposure during adolescence.

In a large study of patients on long-term haemodialysis (*n* = 342,457) in the United States, the ambient UV irradiance at the location of the dialysis clinic was inversely associated with systolic blood pressure [122]. Both ambient UV-A and UV-B irradiance were associated with a beneficial effect, but the effect of UV-B radiation was greater. These data are consistent with an earlier clinical trial of UV-A irradiation [123], but the lack of individual-level data on residential location or time outdoors reduces the level of confidence that greater exposure to UV radiation is associated with lower systolic blood pressure.

### Guidance relating to sun exposure may need to be reconsidered

The World Health Organization and many other government and non-government organisations advise that sun protection is not required when the UV index is below 3. This advice has been challenged previously based on UV radiation data from New Zealand [124] and is supported by a new study from Germany [125]. Using data from nine ground-based monitoring stations from 2007 to 2016, Lehmann and colleagues found that when the UV index is 2, people with Fitzpatrick skin types I and II can receive a minimal erythemal dose of UV radiation in 1.5 h [125]. On rare occasions, the minimal erythemal dose can be exceeded when the UV index is 1. A more nuanced sun protection message may be required that takes account of skin type and time outdoors.

## Terrestrial ecosystems and biodiversity

Interactions between stratospheric ozone depletion and climate change continue to modify the exposure of plants and animals to solar UV radiation (both UV-B and UV-A radiation). These changes have the potential to affect agricultural sustainability and the health and services of terrestrial ecosystems. In this section, we evaluate the ecological strategies that underpin the responses of terrestrial ecosystems to stratospheric ozone depletion, and assess new research that considers the role of UV radiation in influencing the range of suitable habitats for important species that contribute to biodiversity. In addition, we address how UV-B radiation, together with changes in other environmental factors associated with climate change (e.g., temperature, moisture availability), affects plant growth, pathogen and pest defence, and food crop quality. Here we assess the potential effects of these and other changes resulting from interactions between stratospheric ozone depletion and climate change on terrestrial ecosystems, including ecological effects of extreme events such as wild fires, record-setting temperatures, and stratospheric ozone depletion in the Arctic and Antarctic.

### Interactive effects of UV radiation and climate change on biodiversity

Climate change can cause declines in biodiversity by reducing the availability of suitable habitats for plant and animal species. Shifts in the ranges of distribution can disrupt ecosystem functioning and lead to further species migration. Ultimately, if dispersal fails to keep pace with changing habitats, biodiversity loss will occur. Species distribution models are applied to determine how climate change will affect future habitat suitability of valuable species through changes in key abiotic drivers. These models account for landscape-level processes; they do not account for fine-scale effects on microhabitats, including topographic, canopy-level and biotic factors, which are also important in determining where species can live [126, 127]. These species distribution models can be used to inform efforts to conserve species (SDG 2.5), as well as management plans for improving plant production in agriculture and forestry [128].

Several studies have shown that the inclusion of UV-B radiation in models that forecast future distribution ranges of ecologically and agriculturally important species can improve their predictive power [75, 129–134]. These models are based on RCP scenarios (Sect. 2.1) driving future climate change, and suggest that the range of some native species from open, dry habitats will expand to higher elevations [75, 130–134], while the ranges of willows and other related species from wetter habitats will shrink [129].

All these studies have examined species from largely arid and semi-arid shrub-steppe habitats in China and central Asia and have taken the unusual step of including UV-B radiation among their environmental variables (data taken from the global climatology; Beckmann et al. [135]). Using Maximum Entropy (MaxEnt) models to estimate habitat suitability, incident UV-B radiation, precipitation, and temperature, were significant determinants of species occurrence. Such models are based on correlative relationships between climate and species occurrence so cannot identify the mechanisms underlying these predictions. Nonetheless, the findings from these models suggest that this approach could be useful in assessing risks to biodiversity, as well as providing information on potential species distributions and suitable habitats for conservation and planting crops under different scenarios of climate and solar UV-B radiation (contributing towards SDG 2.3).

After selection of the significant climatic variables for these particular studies of species distributions, UV-B radiation was retained in the models. Nevertheless, most modelling studies still fail to test whether UV-B radiation and its interaction with other abiotic stressors are potential constraints on species distributions. As more detailed and accessible UV-B databases become available (Sect. 2.9), it will be possible to routinely include UV-B radiation among climatic variables that are used to predict species occurrence, range shifts and changes in biodiversity over large geographic scales.

### Adaptation of Antarctic flora to UV radiation and extreme climate events

The native flora of Antarctica has evolved protective mechanisms to survive in the severe Antarctic conditions and these adaptations can confer cross-tolerance to cold temperatures, drought, and high solar irradiances, including transient high UV-B radiation due to stratospheric ozone depletion. However, disruptions of atmospheric circulation patterns in the Southern Hemisphere resulting from stratospheric ozone depletion and climate change are also causing large changes in the Antarctic climate (Sect. 4.3), which may exceed the tolerances of certain species. If these climatic changes persist, they would likely threaten the native biodiversity of Antarctica.

The harsh environmental conditions of Antarctica limit the extent of suitable habitats for terrestrial photosynthetic organisms. Of these, cryptogams (lichens, bryophytes and algae) are best adapted to survive under these conditions and they dominate the Antarctic flora. In comparison, the two native vascular plant species are restricted to the Antarctica peninsula [136]. Despite their taxonomic differences, there are parallels in the adaptive responses of Antarctic bryophytes and vascular plants to UV-B radiation. Adaptive strategies to survive exposure to UV-B radiation in the moss, *Pohlia nutans*, include antioxidant enzymes, flavonoid synthesis and photolyases [137–139], whereas in *Ceratodon purpureus* various flavonoids confer photoprotection [140] along with yet to be identified red cell wall pigments [141]. The equivalent protection response of the vascular plant, *Colobanthus quitensis*, to UV radiation is enhanced by forming mutually beneficial relationships between plant roots and soil fungi (i.e., mycorrhizae). These associations rely on a stable ecosystem and support the plant’s floral development and growth, also reducing oxidative stress and membrane damage, protecting photosynthesis and maintaining photoprotection through UV-screening flavonoids [142–144]. In the native Antarctic grass, *Deschampsia antarctica*, a key enzyme in the synthesis of flavonoids (chalcone synthase) matches that present in temperate grasses, including the cereal grains rice and barley [145], indicating a common regulatory role in UV-photoprotection across these species. An understanding of how these organisms tolerate UV-B radiation and the harsh conditions of Antarctica will improve our ability to assess the vulnerability of polar biodiversity to ongoing changes in stratospheric ozone and climate.

### Impacts of stratospheric ozone depletion on Antarctic climate affecting terrestrial ecosystems

The impacts of climate change across the Southern Hemisphere linked to stratospheric ozone depletion have been discussed in previous papers [146, 36] detailing effects on both aquatic [147] and terrestrial [148, 149] ecosystems. Ozone depletion has partially masked the impact of climate change in Antarctica, primarily during summer, by promoting cooler conditions than would otherwise have occurred. As the effects of climate change on Antarctic environments are expected to increasingly overwhelm those of stratospheric ozone depletion later in the century, this masking effect will diminish (Sect. 2.4).

During late 2019 and early 2020, a series of unprecedented climate extremes occurred in the Southern Hemisphere, which included strong Antarctic stratospheric warming [40, 41], an unusually small ozone hole [150, 151], severe bushfires in Australia [45], and heatwave conditions in Antarctica [43]. These conditions were associated with a strong negative state of the Southern Annular Mode (SAM), which has been linked with anomalous tropical convection [41]. Model projections indicate that persistence and variability of the SAM are enhanced by stratospheric ozone depletion, leading to longer lasting coupling between the stratosphere and troposphere [152]. This finding would imply that while the 2019 Antarctic ozone hole was relatively small, the climate extremes that followed during the 2019/20 summer were still potentially exacerbated by ozone depletion, although this remains to be confirmed.

**Fig. 6:**
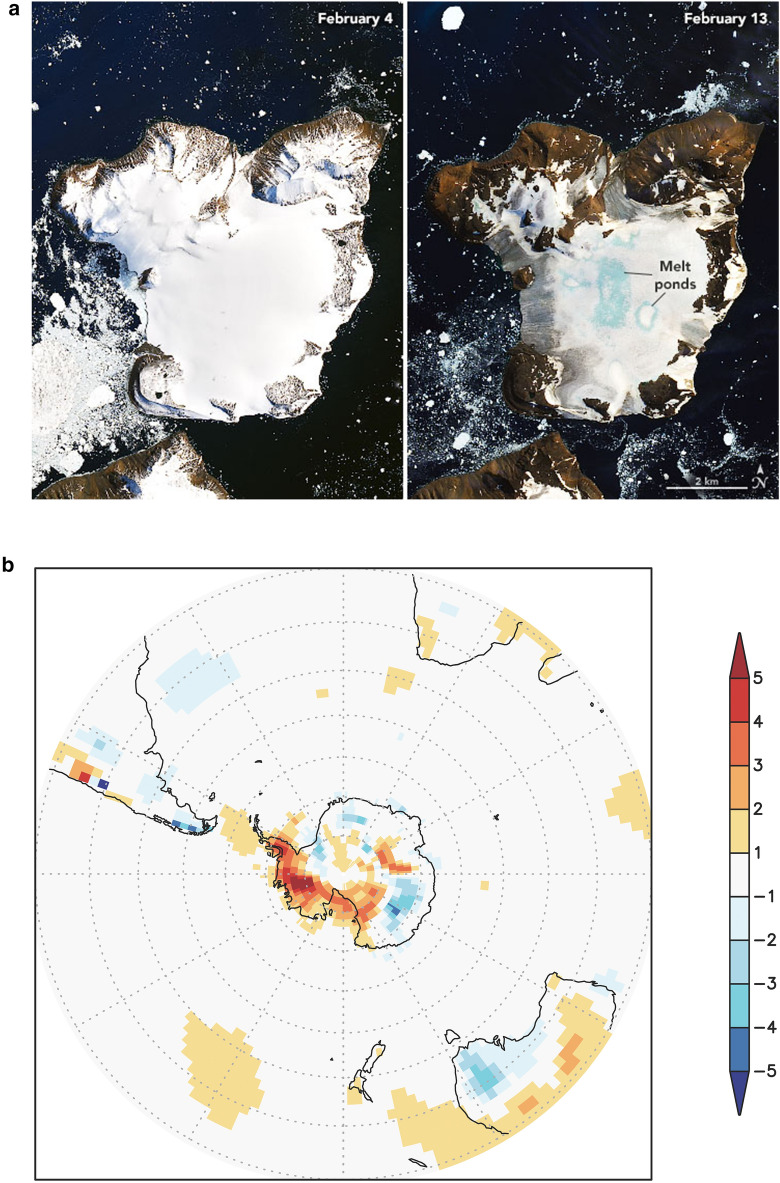
In recent decades, Antarctica has been shielded from some of the effects of global warming by shifts in the climate of the Southern Hemisphere that are related to stratospheric ozone depletion. The Antarctic summer of 2019/20 provides a recent example where the moderating effect of stratospheric ozone depletion on regional climate was likely diminished. An anomalously high total column ozone in the austral spring of 2019 (linked primarily to meteorological factors and not to significantly reduced stratospheric ODS concentrations) resulted from a weak and strongly disturbed polar vortex. Throughout the 2019/20 summer, high temperatures were recorded across Antarctica, which led to melting of ice and exposure of new ice-free areas. **a** Images of Eagle Island showing extensive melt associated with surface warming on 4th and 13th February 2020 (https://earthobservatory.nasa.gov/images/146322/antarctica-melts-under-its-hottest-days-on-record. **b** Monthly mean anomaly (the difference from climatological monthly mean) of 2 m air temperature from NCEP/NCAR Reanalysis 1 data [154] for February 2020. The climatology spans 1979 to 2019 (see S. A. Robinson et al. [43] for other summer months)

The 2019/20 Antarctic summer heatwave lasted 4 months and led to accelerated snow melt (Fig. 6), localised flooding, and greening of previously drought-stressed moss beds [43]. While it is too early to know the full extent of biological changes resulting from the Antarctic summer of heatwaves, we would expect flooding to alter soil invertebrate and cyanobacterial communities in the coming decade as reported following flooding of the Dry Valleys in 2001–2002 [155]. Accelerated snow melt can also lead to depletion of water reserves late in the season, causing ecosystem water deficits in late summer [136]. Antarctic organisms are able to survive in the normally cold air by absorption of solar radiation and passive heating to create microhabitats where plants are often 10–20 ˚C warmer than the ambient temperature [153]. We, therefore, would expect that temperatures like the 20.75 °C reported at Marambio Base on Seymour Island last summer would lead to heat stress in Antarctic organisms [43]. In general, we anticipate that these recent extreme conditions pose significant threats to the survival of some terrestrial and coastal marine plants and animals from Antarctica as well as other high southern latitudes, since their adaptive capacity may be reduced under rapidly changing conditions in these already harsh environments. However, scientific reports on the ecosystem effects of these heatwaves, floods and/or droughts on native Antarctic species will likely take several years to appear.

### Response of crop plants to changing UV radiation and climate conditions

Changes in climate and stratospheric ozone are altering the exposures of plants to UV-B radiation and this is occurring in concert with rising atmospheric carbon dioxide concentrations, extreme air temperatures and more variable precipitation patterns [156]. Because of these environmental changes, some crop plants in tropical and temperate mountain regions are likely to be exposed to increasing UV-B radiation as their most suitable habitat is displaced to higher elevations [149]. Multiple environmental factors interact to affect plant growth and agricultural crop quality, but effects are variable depending on species and growing conditions. In particular, ambient UV radiation combined with other environmental stressors, such as changes in temperature, can drive flavonoid production. These compounds have multiple functions in plants including as UV radiation screening pigments, antioxidants and signalling molecules, and changes in plant flavonoids can carry forward to affect a number of ecosystem processes [157, 158]. Flavonoids occur in agricultural and wild plant species and their concentration in leaves and fruit varies with seasonal weather patterns and herbivory [159–161].

Studies of organisms that live at very high elevations are relevant for understanding how species acclimate and adapt to high UV irradiances under extreme environmental conditions. For example, Maca (*Lepidium meyenii*) is a crop from the high Andes in Peru that can quickly recover from stress induced by acute high solar UV radiation [162, 163]. Similarly, Chinese tallow trees (*Triadica sebifera*), growing in southern China, accumulate more UV-absorbing flavonoids in their leaves with increasing elevation (100 to 1000 m above sea level) [161]. Populations of plants and animals can also genetically adapt to tolerate high UV irradiances. These adaptations are evident in increases in melanin pigment of bird skin with decreasing latitudes [164], and latitudinal changes in flavonoids of crop plants such as broad bean (*Vicia faba*). In this latter example, a field experiment using UV filters showed that the flavonoid profile of a cultivar native to high elevations in Ecuador and Columbia differed from that of a cultivar from Sweden at low elevation [165]. In these cultivars, flavonoids increased in response to the UV radiation they received during growth and there is some evidence that these effects persisted into the next generation [166]. These studies imply that, in environments where high UV-B irradiances are common (e.g., low latitudes and high elevations), it may be possible for farmers to select crop species and cultivars that are well suited to these local UV radiation conditions just as growers do for other climatic factors. These activities would contribute to meeting the SDG target 2.3 of improving the productivity and incomes of small-scale producers.

### Ecological strategies of plants to accommodate changing UV radiation conditions

Changes in vegetation, cloud cover, aerosols and snow cover, as a consequence of climate change, air pollution control measures, wildfires and shifting land-use practices, can rapidly increase or decrease UV-B radiation at Earth’s surface (Sects. 2.7–2.8) [2, 149, 167]. These changes in UV-B radiation are often accompanied by changes in other wavelengths of sunlight (i.e., spectral quality) that then interact with UV-B radiation to modify plant function and the composition and diversity of ecological communities.

Progress continues to be made in identifying the molecular mechanisms that underpin plant responses to UV-B radiation, and how these responses differentiate and interact with responses to the adjacent spectral regions of radiation, such as UV-A radiation and blue light. The eventual goal of this research is to establish the links from perception by the photoreceptor to the functional response of plants within an ecological context [168, 169]. Recent studies report how responses at the genomic, transcriptomic and metabolomic level are expressed in plant phenotypes. This research confirms that different photoreceptors trigger responses governed by UV radiation shorter than 350 nm and responses mediated by UV radiation longer than 350 nm and blue light. However, these two sets of responses interact with each other to coordinate acclimation to high light *vs* shade conditions [170–173]. These findings imply that perception and response to UV-B radiation not only protects against the deleterious effects of very high solar radiation but that many of these photoprotective responses can also be induced by UV-A radiation and blue light.

**Fig. 7:**
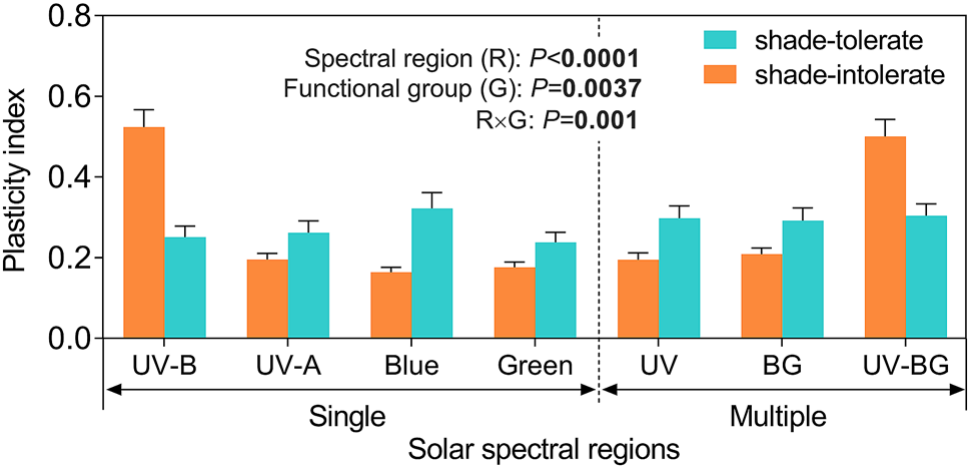
Phenotypic plasticity to spectral regions of solar radiation for shade-tolerant and shade-intolerant species. Light blue and orange bars indicate shade-tolerant (Mean ± 1SE, *n* = 9–11) and -intolerant (Mean ± 1SE, *n* = 3–12) plants growing in a filter experiment. Multiple spectral regions are UV radiation, blue-green light (BG), and UV and blue-green light (UV-BG). Plasticity index was calculated based on the response of 25 functional traits categorised into five groups: biochemistry, physiology, leaf morphology, the whole-plant morphology, and growth and allocation. Figure modified from Q–W. Wang et al. [175]

The UV-B radiation received within plant canopies depends on vegetation structure and follows predictable daily, seasonal, and annual cycles dictated by sun angle, canopy phenology, and the optical properties of leaves in the crown [160, 174]. Recent analyses using plant functional traits, show that shade-intolerant species (i.e., those adapted to grow in fully sunlit environments) acclimate more effectively to shifts in the spectral composition of solar radiation involving changes in UV-B radiation than shade-tolerant species (i.e., those adapted to grow in shaded environments) [175]. For example, shade-intolerant species effectively adjust their leaf epidermal flavonoids, which are important for UV protection, in response to seasonal changes in exposure to solar radiation across both deciduous and evergreen forest stands [174]. These compounds have been found to improve canopy-level photosynthesis (measured as light-use efficiency) of plants during transient periods of high irradiance, sunflecks, and in canopy gaps, by ameliorating photoinhibition and DNA damage [176–179]. By comparison, shade-tolerant species are better at adjusting to other regions of the solar spectrum (UV-A radiation, blue, and green light) but not to UV-B radiation, in traits linked to both photosynthetic efficiency (e.g., chlorophyll fluorescence) and stress tolerance (e.g., total phenolics, principally flavonoids and anthocyanins) [175] (Fig. 7).

These findings suggest that sudden changes in the spectral composition of solar radiation may compromise plant adjustment to a change in its immediate environment (i.e., its phenotypic plasticity). Such changes in solar radiation and its UV component are often abrupt and can, in time, modify plant community composition, since functional strategies for light use (i.e., shade-tolerance and shade-intolerance) vary among species [180, 181]. Changes in community composition, in turn, can reduce biodiversity. Dramatic alterations in forest understorey light environments (especially UV-B radiation) that result from changes in land use and climate could thus lead to the decline of a high diversity of shade-adapted species, which are critical for healthy forests and the services they provide (SDG target 15.1).

### Pollen, UV radiation, and paleoecology

Pollen is essential for reproduction in flowering plants and has attributes that make it a potentially useful tool for reconstructing past solar UV radiation climates and interpreting extinction events in the fossil record. The accumulation of phenolic compounds protects pollen from the deleterious effects of solar UV radiation and these compounds, which are deposited in sporopollenin (the outer structure of the pollen that is preserved in the fossil record), are stable over long time periods [182]. Thus, changes in the concentration of these UV-absorbing compounds in the fossilised pollen of specific plant species may serve as a proxy for global changes in surface UV-B irradiances over geological timescales [183]. The expected stabilisation of global UV-B irradiances following the recovery from contemporary stratospheric ozone depletion reduces the risk that exposure to UV-B radiation will exceed plants’ capacity to protect the DNA in pollen in the future [149]. By comparison, some studies have linked abnormalities in fossilised pollen to mass extinction events in the geologic record, although further evidence is needed to confirm that the observed changes in fossil pollen structure and chemistry are specific to UV-B radiation effects and not the result of other changes in the environment.

Abnormalities are evident in the structure of pollen grains from sediments spanning the end-Permian mass extinction (e.g., Hochuli et al. [184]. Several studies also report pollen abnormalities from sediments spanning other time periods in Earth’s history, including the Late Triassic [185], the Triassic-Jurassic transition [186], and the end-Devonian mass extinction [187]. Such abnormalities in pollen morphology can be caused when a burst of reactive oxygen species (ROS) within the pollen grain produces DNA dimers and introduces mutations that reduce pollen viability, or through direct damage to pollen during its development [188]. Increased occurrence of malformations has been found in pollen from *Pinus mugo* under treatments of high UV-B irradiance [189]. Thus, it is conceivable that the deformations seen in fossil pollen could be the result of an increase in UV-B irradiance due to severe stratospheric ozone depletion in the geological past. However, care is needed in attributing these abnormalities to UV-B radiation, since similar damage can also be caused by metal toxicities, nutrient limitations, heat stress, drought stress, and pathogens, also polyploidy and hybridisation [190, 191]. Likewise, the timescales and geographic ranges of deformation of pollen in the fossil record should match the expected scales of ozone depletion and associated climatic changes [192].

Nevertheless, by combining the information on abnormalities with biochemical studies of pollen, it is possible that stronger inferences can be made about the timing and consequences of major global changes in UV-B irradiance associated with perturbations in Earth’s climate over geological timescales. This knowledge would provide a valuable perspective on the ecological consequences of modern-day climate change and the potential effects on Earth’s biota resulting from large increases in solar UV-B radiation that would have occurred in the absence of the Montreal Protocol [64].

### Technological advances, UV radiation, and agricultural sustainability

The innovative development of technologies in UV-B radiation research, coupled with the application of knowledge of plant response to UV-B radiation from research into the environmental effects of stratospheric ozone depletion, is contributing to more efficient and sustainable agriculture ranging from pre- to postharvest treatments and from field to greenhouse crops. The increase in this kind of innovative research and development has been one of the important indirect benefits of the Montreal Protocol and contributes to meeting the SDG target 2.4 of ensuring sustainable food production. As a consequence of these activities, we have a better understanding of the appropriate amounts of UV-B radiation required to promote regulatory responses in crops without inducing damage. This knowledge is being applied to produce food crops in a manner that is economically viable and environmentally sustainable (SDG 17.14). For practical reasons, the precision application of UV-B radiation is easier in controlled environment facilities [193–196], although supplemental UV-B radiation has also been applied under field conditions (e.g., using UV-emitting lamps mounted on a tractor in vineyards [197]) and where UV-B radiation is manipulated for the control of pests through the use of novel UV-transmitting cladding materials [198].

The role of UV radiation has been studied in the production of about 50 different food crop species, including cereals, legumes, vegetables, fruits, pseudocereals, herbs, and spices, as well as turfgrasses, ornamental plants, and many medicinal plants [199]. In most studies where UV-B radiation has been manipulated, it has been applied prior to harvest (i.e., preharvest), or following harvest (i.e., postharvest). Species studied in this context include beetroot [200], broccoli [201], ginseng [202], prickly pear [203], blueberry [204], and even mushrooms [205]. In certain varieties of peach, these treatments have helped maintain the weight, firmness, and vitamin C content of the fruit, which increases its quality, value, and shelf-life [206–208].

The use of UV-B radiation in plant production can also affect both the physiology and behaviour of insect pests. For example, a daily night-time exposure to 3 h of UV-B radiation decreased the hatching of eggs of the important agricultural pest species, *Tetranychus urticae*, the two-spotted spider mite [209]. A natural predator on this pest species, the mite *Neoseiulus californicus*, was relatively unaffected by UV-B radiation, creating the possibility for integrating UV-B treatments in sustainable cropping. UV-B radiation is also emerging as an environmental cue that can upregulate plant defences against herbivorous arthropods. For example, short daily exposures of UV radiation increased the resistance of chrysanthemum (*Chrysanthemum* × *morifolium*) to the insect pest, thrips (*Frankliniella occidentalis*) [210].

UV-B radiation technology is continuously improving, especially with the development of cheaper, more energy-efficient narrow waveband light-emitting diodes (LEDs) [211, 212]. However, legal restrictions limit the use of UV-B radiation sources in controlled plant production in some countries [213]. In addition to LEDs, attaining the appropriate amounts of UV-B radiation often requires the use of optical filters [214] and simulation models to estimate appropriate time-integrated UV-B doses [215]. For these technologies to be widely adopted and approved, however, proper precautions must be taken to protect human health against adverse effects of UV-B radiation [216].

## Aquatic ecosystems

The exposure of aquatic ecosystems to solar UV-B radiation depends on the extent and rate of stratospheric ozone recovery and many additional factors that are progressively changing. The most important factors determining the transmission of UV radiation in aquatic ecosystems are the amounts of particulate and dissolved material in the water column. Inland and coastal waters are generally less transparent to UV radiation than oceans and large lakes, primarily due to terrestrial run-off carrying decayed plant material [217]. Climate change is predicted to affect penetration of UV radiation by altering the amount of runoff, and thus water clarity (transparency), as well as depth of mixing and the duration of ice-free conditions. Seasonal variations in exposure to UV radiation are modulated by the timing of runoff and the photobleaching of dissolved organic matter (DOM). Aquatic micro-organisms, macroalgae, plants, and animals (floating, swimming and attached) respond to these changes in UV irradiance, with the response also dependent on other effects of climate change including warming and ocean acidification (SDG 14.3). Substances released into the environment by humans, such as oil pollutants, are also modified by UV radiation, which in turn can affect aquatic organisms.

### The role of dissolved organic matter in controlling underwater exposure to UV-B radiation in lakes has been confirmed over broad geographic regions, including China

Underwater exposure to UV-B radiation is a function of the amount of UV-B radiation incident on the surface, as well as absorption and scattering by water and substances in the water. As the stratospheric ozone layer continues to recover, changes in the quality and quantity of UV-absorbing terrestrially derived dissolved organic matter (tDOM) are increasingly controlling exposure to UV-B radiation in inland and coastal waters [218, 219]. Prior studies focused on sub-tropical, temperate, and Arctic regions of North America. Now, trends in incident and underwater UV radiation in lakes have also been compared for China. For three lakes between 1988 and 2014, the penetration of UV-B radiation at 1 m depth relative to incident has decreased by 12 to 45% [220]. Incident UV-B radiation also decreased during this period, so that overall UV-B radiation at 1 m depth decreased by 14 to 46%. This decreased water transparency, mostly from higher concentrations of tDOM, apparently accounted for most of the decline in UV-B radiation, with decreased incident radiation, mainly due to increased aerosol pollution, playing a minor role [220]. This study supports the key role of tDOM in controlling underwater exposure to UV-B radiation compared to factors controlling incident radiation, including aerosols and ozone.

### The seasonal timing of incident UV-B radiation relative to seasonal changes in hydrology, photobleaching, and water temperature, regulate underwater UV-B radiation and the response of aquatic organisms

Seasonal cycles in incident solar UV-B radiation, hydrology, and photobleaching (reduction in the UV-absorbing capacity) of tDOM alter underwater exposure to UV-B radiation. Changes in the seasonal timing of both maximum and minimum water temperatures relative to the timing of peak exposure to UV-B radiation may have deleterious consequences for some aquatic organisms. For example, as spring progresses to summer and temperatures warm and increase evapotranspiration, the transport of UV-absorbing compounds in tDOM to inland and coastal waters decreases [221]. Biodegradation and photodegradation of tDOM additionally changes the chemical composition, decreasing the UV-protective capacity of tDOM [222]. In the shallow waters of coral reefs, high temperatures together with high UV-B irradiance threaten coral health [223]. The seasonality of photodegradation of DOM, which increases transparency of water to UV-B radiation, can shift the timing of peak exposure to the UV-B radiation by a month or more after the summer solstice. As surface temperatures continue to rise during this period, the most stressful periods of high temperatures and high UV-B radiation coincide [223]. In contrast, in higher latitude or high elevation lakes, water temperatures warm more slowly than daily increases in incident solar radiation, leading to a seasonal time lag that creates seasonal periods when exposure to damaging UV-B radiation is high, but water temperatures remain low [224]. These lower temperatures are likely to reduce the potential for organisms to effectively repair UV-B-induced DNA damage due to the temperature-dependence of the enzymes that drive photoenzymatic repair [225, 226]. Thus, seasonality of the hydrologic cycle and bio- and photodegradation of tDOM play a major role in regulating underwater exposure to UV-B radiation in aquatic ecosystems, while the seasonality of temperature in relation to the timing of peak incident solar UV-B radiation can determine how effectively aquatic organisms can respond to UV-B radiation. All of these factors are being affected by ongoing climate change.

### Climate change is causing regional increases or decreases in the depth of vertical water circulation in the surface layer, which determines the average exposure to UV-B radiation for water, particles, and planktonic organisms

Surface waters in the ocean and lakes continuously move, both horizontally and vertically, mixing substances and planktonic organisms. The upper mixed layer (UML) represents one of the most biologically active habitats within aquatic ecosystems. During vertical circulation, substances and organisms in the UML are exposed to variations in UV-B radiation, higher at the surface, decreasing with depth. The average exposure level depends on the depth of the circulation, the mixed layer depth (MLD), which is an important variable to consider when assessing the effects of UV-B radiation in pelagic systems. This depth differs depending on physical factors, including gradients in temperature and salinity, water clarity, and wind speed. The effects of climate change on these factors vary among different parts of the ocean [227]. Contrary to earlier projections that climate change should lead to uniformly shallower MLDs in the ocean (e.g., [228]), more recent data show that trends are not always towards shallower depths and that MLDs are, in some locations, deepening [229].

Inland there is the phenomenon of “atmospheric stilling”, where the strength of surface winds has declined over many continental areas from 1980 until 2010 [230], after which wind speeds have increased [231]. Stetler et al. [232] suggest that the decline in wind strength led to a shallower summer MLD in deep, remote Crater Lake, Oregon, United States. The causes and future of atmospheric stilling are unclear, as is whether the reversal in atmospheric stilling will continue in the future [231, 233]. Stilling increases average exposure to UV-B radiation in the UML.

While it is clear that climate change affects MLDs and, therefore, exposure to UV-B radiation in aquatic ecosystems, impacts differ among locations, and it is the balance among several factors that determine whether exposure increases or decreases in a particular system.

### The effects of climate change on ice cover are increasing underwater exposure to UV-B radiation

Ice cover shades water from UV-B radiation, and ice-covered areas are decreasing in lakes [234] and the polar oceans [235] due to climate change. The date of ice breakup in 152 lakes in the Northern Hemisphere during 1951–2014 shows a trend of 1.2 days earlier breakup per decade (range 5.0 days earlier to 0.3 days later) [236]. Using future scenarios of climate change (RCP 2.6 and RCP 6.0), the projected duration of ice cover on historically ice-covered lakes is 15 (RCP 2.6) or 29 (RCP 6.0) days shorter in the period 2080–2100 than for 1985–2005 [234]. Similarly, an ice cover classification model was used to project the future ice cover of the almost 1.5 million lakes within the global HydroLakes database [237]. The proportion of ice-covered lakes shifting from having ice cover every winter to only some winters would be 2.8% at a temperature increase of 2 °C and 7.2% at a temperature increase of 4.5 °C. Exposure to UV-B radiation will increase in historically ice-covered lakes affected by such a future loss of ice cover.

### Surface features such as meltwater ponds and snow cover are major determinants of how much UV-B radiation is transmitted through ice cover

The amount of light transmitted through ice cover varies greatly depending on snow cover and the presence of meltwater ponds on the ice [238]. Studies of transmittance through ice in the Arctic Ocean found that monthly average transparency in the 400–550 nm band varied between 1 and 17% [239]. Surface melt ponds and scattering layers (mainly snow) were the main determinants of transmittance. The thickness of the ice was less important, explaining only 16% of the variation in transmittance. Based on these results, pond presence would be expected to also strongly affect transmittance of UV radiation. Confirming this, the transmittance for all wavelengths (UV radiation and PAR) was strongly enhanced by the presence of ponds on the ice in Baffin Bay [240, 241]. When ponds developed, there was a tenfold increase in transmittance for PAR and UV-A radiation (325, 340 and 379 nm). Twice as much UV-B (305 nm) radiation was transmitted in ponded ice compared to that in white ice, reaching values between 11 and 14%, at 2 m depth. For short UV-A wavelengths (e.g., 325 nm) transmission was much higher, reaching 22–35% when ponds were present. One can assume that for the longest UV-B radiation wavelengths (e.g., 315 nm), transmission would be somewhere between those values. This effect on transmission should be considered in projecting the future trend of UV-B radiation exposure underneath the sea ice, as increased pond formation is correlated with the faster melting of seasonal sea ice increasingly seen for the Arctic [242].

### Harmful algal bloom species are resistant to UV radiation and become more toxic when UV radiation is present during growth

Nutrient pollution and harmful algal bloom (HAB) species threaten marine and freshwater environments globally [243]. The marine dinoflagellate, *Karenia mikimotoi*, causes HABs in coastal waters around the globe and accumulates a toxin that kills fish by damaging red blood cells (hemolytic toxicity). The growth rate of this species in culture was unaffected by solar UV-B + UV-A radiation or ocean acidification (or the two in combination) [244], but both conditions enhanced hemolytic toxicity. In freshwaters, the photosynthetic bacterium (cyanobacterium), *Microcystis aeruginosa,* forms HABs in surface waters where it is exposed to high UV radiation. These surface blooms prevent light and oxygen from entering deeper waters and some strains produce another powerful toxin, microcystin. Ren et al. [245] measured growth and photosynthetic potential (*F*_v_/*F*_m_) of a toxic strain of *M. aeruginosa* exposed to a combination of UV-B and UV-A narrowband lamps. The weighted exposure approximated summer midday surface irradiance. When supplied with ample phosphorus (P) as would occur in a eutrophic lake, photosynthesis and growth of *M. aeruginosa* were little affected by UV radiation compared to moderate or severe inhibition under low or depleted P conditions, respectively. This is consistent with the general pattern shown in earlier studies of higher resistance to UV (including UV-B) radiation when algae are nutrient sufficient [246]. When exposed to high UV-B+UV-A+PAR irradiance from a solar simulator, photosynthetic potential was more inhibited in toxic *vs* non-toxic strains of *M. aeruginosa* [247]. However, the toxic strain recovered faster after exposure to UV radiation. Thus, the net effect over a daily time scale was less on the toxic strain, which was further confirmed by its higher growth rate than the non-toxic strain over a 7-day period with daily exposure to UV radiation. Moreover, exposure to UV radiation enhanced the accumulation of microcystin in the toxic strain [247]. Thus, exposure to UV radiation enhances toxicity in both freshwater and marine HABs. These results could help inform when toxicity is more likely to occur in a HAB outbreak (SDG 3.9, 6.1, 6.6).

### UV-B radiation negatively affects zooplankton but protective mechanisms mitigate these impacts

**Fig. 8:**
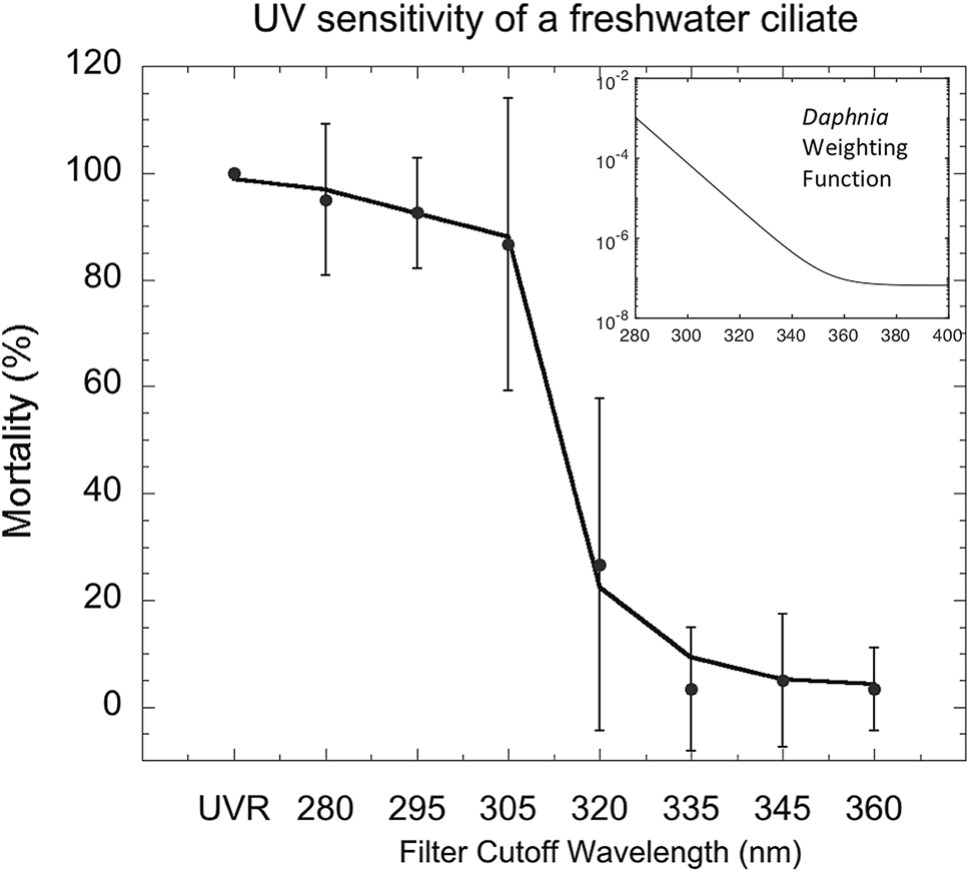
Effect of UV irradiation on mortality of the mixotrophic ciliate, *Pelagodileptus trachelioides*. Laboratory exposures to a UV-B + UV-A lamp, either unfiltered (UVR) or filtered to block wavelengths shorter than indicated. Data points: average mortality ± standard deviation (*n* = 12). Line: predicted mortality as a function of spectral irradiance weighted by the *Daphnia* survivorship biological weighting function (inset, [257]). Only treatments with UV-B radiation caused significant mortality. Redrawn from [253] using author-provided spectral irradiance data

Zooplankton are key links in aquatic food webs; they feed on primary producers and are themselves key prey for fish. Hence, a reduction in the abundance of zooplankton due to UV radiation can lead to reduced fish production and the development of phytoplankton blooms. Evidence continues to accumulate that excessive solar and laboratory exposure to UV-B radiation leads to DNA damage and, ultimately, mortality in zooplankton. This evidence now extends to less studied taxa such as ciliates (single celled organisms with hair-like structures, cilia, for movement), crab larvae, and under-represented systems such as marine and tropical systems (Fig. 8) [248–254]. Apart from mortality, UV radiation also affects zooplankton at a sub-lethal level leading to reduced fitness. Recent studies have demonstrated reduced feeding rates in small crustaceans such as copepods, reduced growth in *Daphnia* and morphological changes in structures used for swimming and feeding in ciliates when exposed to PAR, UV-A and UV-B radiation compared to PAR-only treatments [251, 253, 255, 256].

Zooplankton use defenses against UV radiation such as the accumulation of photo-protective compounds through diet, enzymatic defenses, cell repair systems and changes in behaviour to mitigate UV-induced damage [258–261]. However, changes in stratospheric ozone and climate change affect radiation exposure, challenging the limits of these natural defenses [147]. Recent examples show that pigmentation makes crab larvae more resistant to solar UV-A and UV-B radiation. However, this defense comes at the cost of increased predation risk, since the strategy to avoid predation includes both low pigmentation and induction of a defensive spine [249, 250, 262]. Furthermore, melanin pigments reduce DNA damage (cyclobutane pyrimidine dimers) in pigmented *Daphnia* and increase survival compared to more transparent *Daphnia* when exposed to combined UV-A and UV-B radiation treatments [254]. The differential photo-protective function of melanin is even present within clones that periodically have lower tolerance to UV radiation when the melanin carapace (outer shell) is exchanged during molting [254]. To summarise, zooplankton can adapt to some changes in UV radiation, but exposure to excess UV-B radiation, as would likely have occurred without the Montreal Protocol, leads to DNA damage, reduced fitness, changes in behaviour, and ultimately mortality.

### Exposure to UV-B radiation has multiple negative effects on all life history stages of fish, including commercially important species essential for food security in many regions of the world

**Fig. 9:**
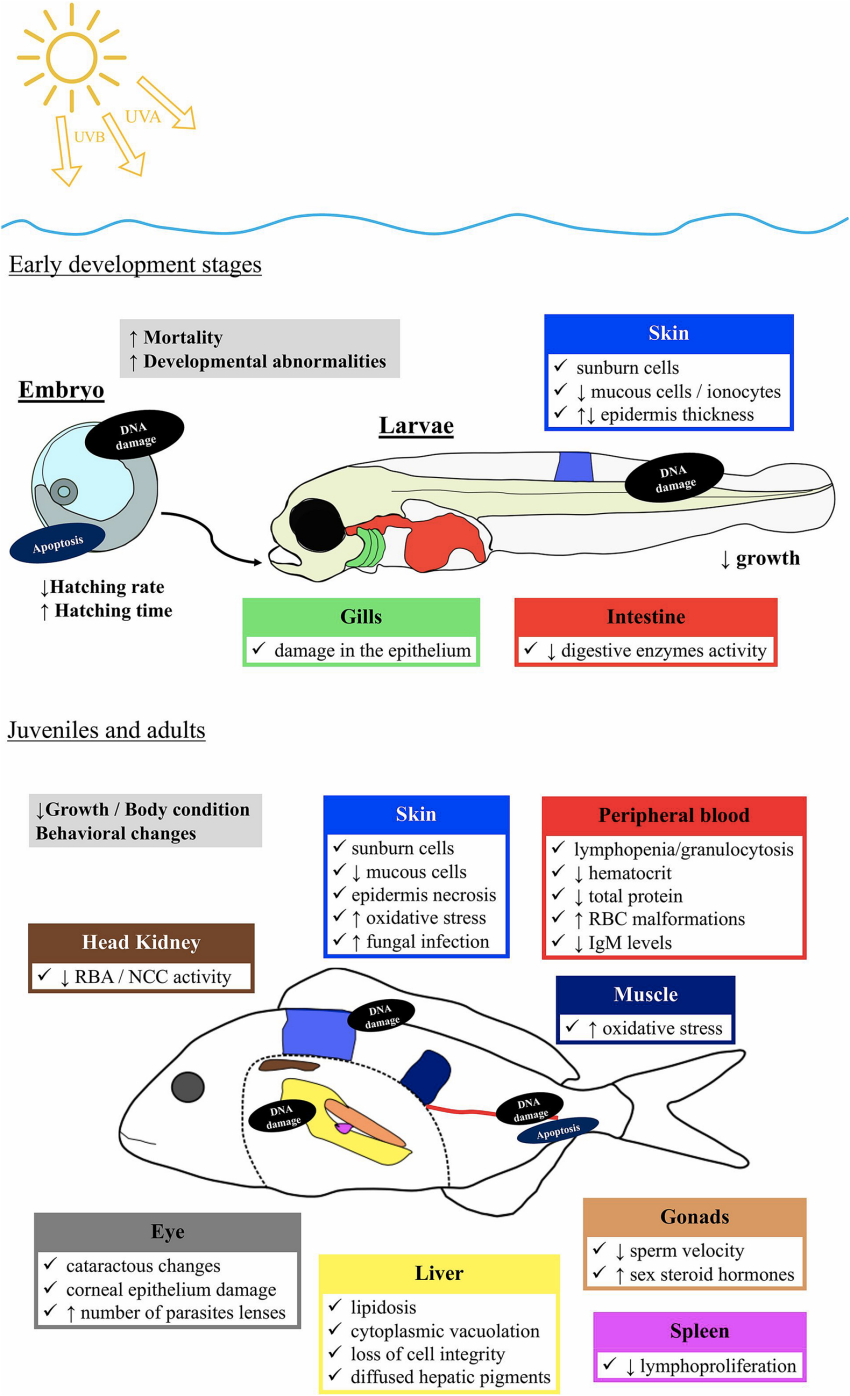
Effect of ultraviolet radiation on the life stages of fish. RBA, respiratory burst activity; NCC, non-specific cytotoxic cells. From Alves and Agusti 2020 [263]

There are a wide variety of negative effects of UV-B radiation on fish embryos, larvae, juveniles, and adults (Fig. 9). Negative effects range from developmental abnormalities, to decreases in growth and body condition, to lesions in skin, eyes, as well as in many other organs, and even death [263]. Often, the more transparent, younger life-history stages are vulnerable to UV-induced damage, in particular because they typically inhabit shallow, warmer surface waters to increase developmental rates. The skin and gills are the most highly exposed and most likely sites of damage by UV-B radiation, although immune suppression, visual impairment, and other damage is commonly reported in both natural populations and hatchery-raised fish [263]. The corresponding reductions in fish survival, growth, and reproduction have as yet unquantified implications for the yield of commercial and recreational fisheries that provide critical supplies of food to much of the world’s population (SDG 2.4).

### Warming and nutrient enrichment are antagonistic to the negative effects of UV-B radiation on aquatic ecosystems, while ocean acidification can be antagonistic, neutral or synergistic

Most of the studies on productivity in marine ecosystems have focused on single environmental parameters. In contrast, recent research has indicated that interactions between key stress factors modify the response to individual stressors (reviewed by [264]). Increasing temperatures due to climate change, acidification by enhanced CO_2_ concentrations, variation in upper mixed layer depth (MLD) (Sect. 5.3) and changes in nutrient availability modify the effects of exposure to solar UV-B radiation in primary producers. A meta-analysis based on 1139 assessments of terrestrial, freshwater and marine biota showed that, in general, the two stressors (UV-B radiation and temperature) acted antagonistically with elevated temperature (below lethality) enhancing resistance to increased UV-B radiation [265]. In the macroalga, *Porphyra yeozensis,* higher temperatures strongly decreased the inhibition by UV radiation and reduced the stress by high PAR [266]. Ocean acidification can have synergistic, neutral or antagonistic effects on the response of marine photosynthetic organisms to UV radiation [267]. The interaction between UV radiation and nutrient availability was studied in an in situ freshwater food web using high and low concentrations of dissolved organic carbon (C) and inorganic phosphorus (P) under UV irradiation. High organic C and P concentrations resulted in stronger resistance to UV radiation [268]. This field experiment showed that lower sensitivity to UV radiation alone or UV radiation combined with another stress factor gives an organism an advantage over other members in the ecosystem and thus changes the composition of the biota and the structure of the microbial food web [268].

### Synthetic UV sunscreen compounds may be toxic to adult corals and their larvae

While the long-term benefits of sunscreens in preventing keratinocyte cancers and melanoma in humans are well demonstrated, some compounds used in commonly available sunscreens are environmental contaminants of concern (SDG 12.4, 14.1) [269–272]. Jurisdictions have banned some sunscreen compounds, including oxybenzone (also known as benzophenone-3), octinoxate, and octocrylene, based, in part, on measured environmental concentrations in coastal zones and on laboratory experiments, indicative of their potential toxicity and contributions to coral bleaching [273]. This has motivated research on the use of natural sunscreens called mycosporine-like amino acids [274], which are synthesised, for example, by many species of macroalgae and cyanobacteria [275, 276].

Some active ingredients in UV sunscreens are toxic to corals, such as ethylhexylmethoxy-cinnamate (also known as octinoxate), octocrylene and benzophenones. Effects include mortality and bleaching (loss of symbiotic algae) in adult corals [277] and arrested development or mortality in their larvae [278]. These compounds also accumulated in coral tissues, but exposure concentrations (~ 0.5–3.5 parts per million) were substantially higher than found in most natural systems. By comparison, sunscreen compounds such as oxybenzone (a benzophenone) have been found in coral tissues and at concentrations of 0.1–136 parts per trillion (ng/L) in coastal waters near Oahu, Hawaii [279] and 114, 11, and 118 parts per trillion in Chesapeake Bay water, sediments, and oyster tissues, respectively [280]. These concentrations are substantially (up to ~ 1000x) below lethal thresholds for aquatic organisms such as some algae, zooplankton, and fish [281]. Across a large study of benzophenones, over 90% of sites had concentrations below predicted no-effect concentrations [282]. While these results suggest that lethality risk is low in most locations, continued evaluation of sub-lethal effects at lower doses is warranted given the detection of sunscreens in many organisms.

Climate change may amplify the toxicity of sunscreen compounds. For example, sunscreen toxicity of both organic and inorganic compounds to sea urchins, diatoms, and amphipods increased with increasing salinity [283], and salinity is increasing in regions where evaporation exceeds precipitation. Separately, exposure of organisms to the sunscreen oxybenzone (BP-3) under warmer temperatures (23 *vs* 18.5 °C) increased gene expression associated with detoxification, the endocrine system, and stress responses [284]. Together, these results indicate potential greater sunscreen toxicity in warmer, more saline conditions, which are predicted in many regions under future climate scenarios.

### Interacting effects of solar UV irradiation, ocean warming and acidification increase toxicity of oil pollutants in reef environments

A review of the impact of oil pollution in combination with multiple stressors to shallow reef environments revealed that three co-factors, namely, UV radiation, elevated temperature and low pH (acidification) collectively increase toxicity of oil pollutants (SDG 14.1) [285]. The impacts of oil pollution on coral reef taxa can be exacerbated by environmental conditions commonly encountered in tropical reef environments. Shallow coral reefs are routinely exposed to high levels of UV radiation, which can substantially increase the toxicity of some oil components through phototoxicity. Many oil components strongly absorb UV-B radiation [285]. Exposure to UV radiation represents the most likely and harmful environmental co-factor, leading to an average toxicity increase of 7.2-fold across all tests carried out. Elevated temperature and low pH increased the toxicity of oil exposed to UV radiation by 3.0- and 1.3-fold, respectively. These studies point to the value of including UV radiation, and in particular UV-B radiation, in future oil toxicity studies to identify realistic hazard thresholds.

### Decreased exposure of pathogens to UV radiation in aquatic ecosystems could lead to more epidemics among aquatic organisms

Various pathogens, including viruses, bacteria and parasites, are released into surface waters because of human activity but their infectivity decreases due to several processes including exposure to UV radiation (Sect. 9; SDG 3.3). Here we focus on naturally occurring pathogens in aquatic ecosystems. These aquatic pathogens are sensitive to UV-B radiation-induced DNA damage and to reactive oxygen species produced by both UV-B and UV-A radiation [286–291]. Comparing the sensitivity to natural sunlight (including visible and UV radiation) of the *Daphnia* parasite, *Pasteuria ramose* from different lakes, revealed that strains derived from lakes with high transparency to UV-B radiation had a higher tolerance to full solar radiation compared to strains coming from less transparent lakes [290]. It has been hypothesised that pathogens generally have lower tolerance to UV radiation compared to their aquatic hosts [291, 292] and many parasite–host relationships are known to structure communities and ecosystems [293]. Recent advances in our understanding of the effects of solar radiation on pathogen–host relationships have shown that zooplankton parasites, such as *Pasteuria ramose,* are most sensitive to UV-B radiation, more sensitive to UV-A radiation than visible light, and finally more sensitive to UV radiation compared to its host [292]. Hence, zooplankton can potentially have a parasite-free refuge at depths where most of the detrimental UV-B radiation has been attenuated and where they are only exposed to moderate UV radiation [292]. With the knowledge available regarding host–parasite–UV interactions one can speculate that decreases in exposure to UV radiation, such as those fueled by climate change (Sect. 5.1), have the potential to enhance epidemics among aquatic organisms [291, 292].

## Biogeochemical cycles in the environment

Changes in solar UV radiation and climate have direct and indirect effects on the way in which cycling of elements (e.g., carbon, nitrogen, and sulfur cycles) and the fate of contaminants are occurring in aquatic and terrestrial ecosystems. It is well established that a wide range of synthetic chemicals such as pesticides, pharmaceuticals and other controlled substances are intentionally or accidentally released into the environment by human activities. Solar radiation contributes to the degradation of many of these contaminants and plays a significant role in reducing their concentrations in the environment. Direct photodegradation of these contaminants is mainly driven by UV radiation. Both UV (UV-B and UV-A) and visible radiation are responsible for driving indirect photodegradation processes, with UV radiation being the more important of the two. Climate change, which influences temperature and precipitation levels, also leads to changes in the chemical composition of surface waters, namely dissolved organic matter and oxygen concentrations, which in turn will affect indirect photochemical processes.

In this section, we update the current understanding of photodegradation of natural organic matter in terrestrial and aquatic ecosystems. Photodegradation produces carbon dioxide (CO_2_) as well as biologically labile photoproducts that can be more easily degraded by decomposer organisms (bacteria and fungi) in a process known as photo-facilitation. Research on the cryosphere has continued to quantify the permafrost carbon feedback that releases the greenhouse gases, CO_2_, methane and nitrous oxide, from thawing permafrost in the Arctic. New data and models are assessed on the photochemical decomposition of controlled substances and other contaminants. Finally, we assess the sunlight photoinactivation of biological contaminants, including pathogens and their indicator organisms.

The science assessed indicates that through the regulation of ozone depleting substances, many of which are also greenhouse gases, the Montreal Protocol is estimated to have contributed significantly to slowing climate change by reducing CO_2_ and other greenhouse gas emissions from terrestrial and aquatic ecosystems [294]. This has likely lessened the climate effects on photodegradation of natural organic matter. In addition, the reduced warming from greenhouse gases may have slowed the melting of ice and permafrost in the Arctic, thereby reducing photodegradation of covered, underlying organic matter to CO_2_. The undesirable photochemical conversion of macroplastics to micro- and nano-plastics also may have been lessened by the actions of the Montreal Protocol. Specific examples are provided of relationships between biogeochemical cycles and the SDGs 3, 6, 12, 13, 14, and 15, with an emphasis on carbon and nitrogen cycling in terrestrial and aquatic systems, and the cryosphere; as well as on chemical and biological contaminants.

### Dryland ecosystems are primary locations for carbon cycling via litter photodegradation, but photodegradation can facilitate cycling also in wet ecosystems located in temperate and tropical forests

**Fig. 10:**
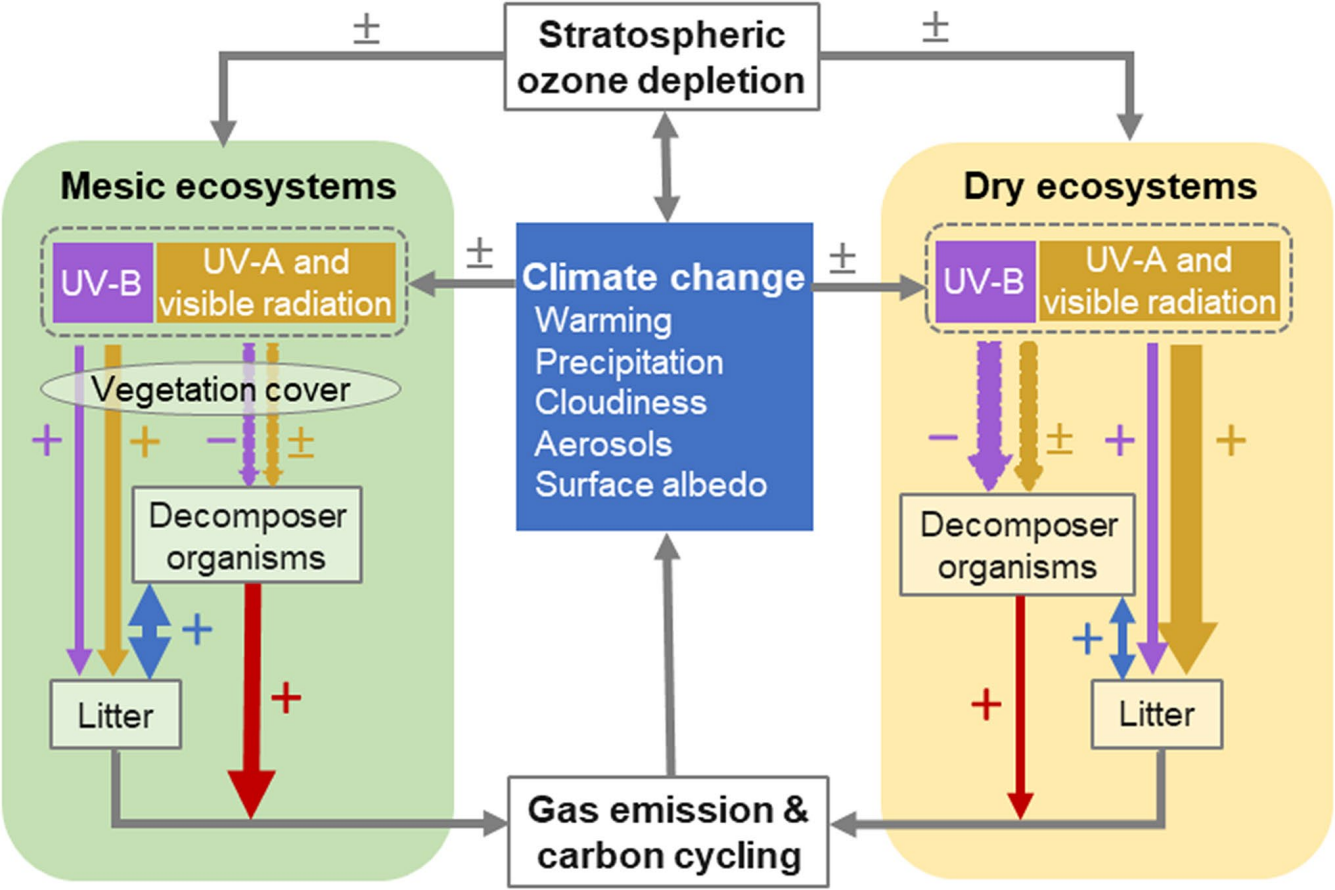
Potential effects of solar radiation and climate change on photodegradation of organic matter, greenhouse gas emissions, and carbon cycling in terrestrial ecosystems. Symbols: grey arrows indicate interactions between changes in stratospheric ozone, solar radiation, and climate changes on litter decomposition in mesic and dry ecosystems. Purple (solar UV-B radiation) and yellow (UV-A and visible radiation) arrows indicate direct effects on litter degradation (photomineralisation) and photoinhibition of decomposer organisms (dashed outline). Blue arrows indicate the process of photo-facilitation. Red arrows indicate the microbial contribution to litter decomposition. The symbols + and – indicate increased or decreased effect on a change or process, and thickness, its importance. Figure designed by Qing-Wei Wang

Recent assessments have emphasised the important role played by solar radiation in driving carbon cycling through litter decomposition to CO_2_ in dryland ecosystems [149, 167]. Since our last assessment [1], additional studies have provided more details about the significance of photodegradation in dry systems including an arid grassland [295], the Sonoran Desert [296], a semi-arid scrub system [297], and a semi-arid woodland ecosystem [298, 299] (contributing towards SDG targets 15.1, 15.3). Other studies since our last assessment have shown that photodegradation is also associated with plant litter (dead plant material) decomposition in wetter ecosystems located in temperate and boreal forests [300–302]. Furthermore, solar radiation drives photodecomposition of litter in tropical [303] and subtropical monsoon forests [304]. These studies provide added evidence that direct photodegradation, and indirect photochemical changes in quality of litter through photo-facilitation, which increases biological degradation, contribute to increases in emissions of CO_2_ to the atmosphere (Fig. 10).

Litter chemistry can also influence direct (abiotic) and indirect (biotic, i.e., photo-facilitation by microbes) decomposition processes [1]. For example, lignin fractions have been thought to be preferentially photodegraded because they strongly absorb solar radiation. However, hemicellulose and cellulose were found to be the plant constituents primarily affected by solar radiation in a mid-latitude semi-arid ecosystem [297, 305], rather than lignin as previously thought.

Photodegradation has generally been neglected in carbon budgets from productive forested ecosystems where litter is exposed to a heterogeneous radiation environment due to vegetation cover. However, recent field studies have found evidence that photodegradation contributes to carbon loss in forests of tropical regions [303] as well as those at temperate mid- [300] and even high latitudes [302]. Some studies have suggested that photodegradation can partly explain the missing carbon loss in these systems, leading to underestimation of terrestrial emissions of the carbon [298, 306]. A recent experimental study attributed much of this missing carbon to atmospheric greenhouse gas emission during photomineralisation [299]. However, changes in solar UV radiation resulting from interactions between stratospheric ozone depletion and climate change can potentially affect the emissions of greenhouse gases resulting from decomposition in an array of terrestrial ecosystems occupying a wide range of latitudes [2, 167].

Changes in vegetation cover can modulate photodecomposition by altering the irradiance that reaches litter surfaces [167]. On a global basis, Earth observation satellite data showed that total forest area declined by 3%, from 4128 million hectares in 1990, to 3999 million hectares in 2015 [307]. The tropics were the only region to show a trend, with forest loss increasing by 2101 square kilometres per year. The effects of these changes in vegetation are poorly defined, but recent studies indicate that land-use changes that increase the exposure of plant litter to solar radiation will accelerate carbon losses from these ecosystems because of photodegradation and photo-facilitation [222, 167, 297, 299, 300, 303, 308–311].

### Wavelength effects have been determined to improve models of carbon cycling in aquatic and terrestrial environments

Spectral weighting functions quantify the relative effectiveness of radiation, by wavelength, on photodegradation processes in aquatic [312] or terrestrial [296] environments. These functions are used to evaluate and compare the results from experiments examining the effect of radiation. Clark et al. (2019) have developed a useful model that uses weighting functions to describe spectral changes in coloured dissolved organic matter (CDOM) during photobleaching of waters from marsh and estuarine systems in the mid-Atlantic United States [313] (contributing towards SDG 15.1).

**Fig. 11:**
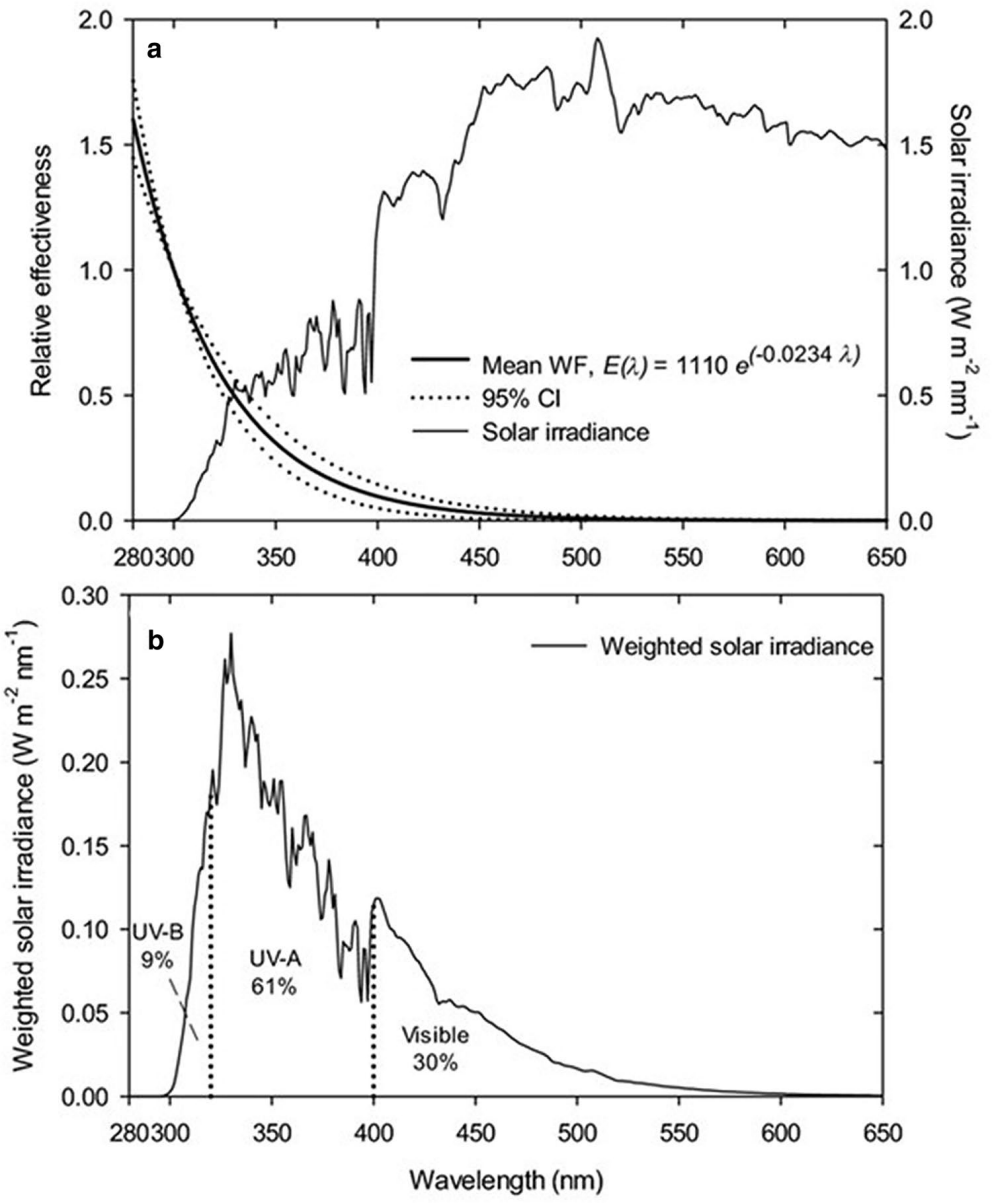
**a** Mean weighting function for photodegradation to CO_2_ from all litter types, with 95% CI, along with average solar noon spectral irradiance; **b** weighted solar noon irradiance, along with the total percentage effectiveness of the solar UV-B, UV-A and visible wavebands. Adapted from Day and Bliss [296]

In terrestrial ecosystems, spectral weighting functions developed for photodecomposition of litter from the Sonoran Desert [296] showed that UV-A radiation was the most effective region of the solar spectrum in the photodegradation of leaf litter (Fig. 11). The weighting functions for emission from three litter types were very similar with effectiveness declining with wavelength but extending well into the visible waveband. Multiplying the weighting function by the average solar irradiance at noon provided the weighted spectral solar irradiance. UV-B, UV-A, and visible wavebands were computed to be responsible for 9, 61 and 30%, respectively, of the observed photochemical emission. This result is generally consistent with earlier findings of the effectiveness of different solar wavebands on the loss of terrestrial litter. Also, the results show that the reductions in stratospheric ozone depletion by the Montreal Protocol and related decreases in UV-B radiation, cause a reduction in photoproduction of CO_2_ from the plant litter. Research has continued to quantify the permafrost carbon feedback that involves release of greenhouse gases from thawing permafrost in the Arctic [1]. Experiments on the wavelength dependence of the photodegradation of dissolved organic carbon (DOC) in permafrost indicated that also UV-A radiation was most efficient [314]. Laboratory manipulations indicated that variability in the photodegradation may be attributable to iron catalysed photoreactions of old DOC derived from soil lignin and tannin in the melted permafrost. Rates of CO_2_ photoproduction from permafrost DOC were twofold higher than for modern DOC [314]. Model predictions of future net loss of ecosystem carbon from thawing permafrost do not include the loss of CO_2_ to the atmosphere from DOC photodegradation. Therefore, current estimates of additional global warming from the permafrost carbon feedback are too low.

### Exposure to UV radiation has pronounced effects on the dissolved organic matter in surface waters ranging from the tropics to the Arctic

One of the largest impacts of climate change on aquatic photochemistry involves changes in concentrations of dissolved organic matter in surface waters. Climate change models predict enhanced precipitation in many places across the globe, which leads to increased loading of the coloured component of DOM (CDOM) into lakes and rivers, a phenomenon referred to as browning (Sect. 5.1) [147, 167]. Runoff can be a major source of organic matter in aquatic environments. Solar UV radiation drives the photodegradation of terrestrially derived DOM and particulate matter that is present in aquatic ecosystems [167, 222, 309, 315, 316]. As in terrestrial ecosystems, photodegradation of aquatic organic matter occurs by direct (abiotic) and indirect (biotic, i.e., photo-facilitation by microbes) decomposition processes; reactive oxygen species are key intermediates in indirect photodegradation [222].

One of the major sources of DOM involves release by melting of the permafrost in the Arctic [167, 314, 317, 318]. Current research on photodegradation by UV radiation of Arctic dissolved organic matter [318] shows that formation of CO_2_ is accompanied by buildup of a pool of partially photooxidised DOM that likely plays a role in photo-facilitated microbial degradation [167]. This partially photooxidised DOM forms via indirect photodegradation involving the intermediacy of singlet oxygen [318], a widespread reactive oxygen species that is produced by a UV-initiated photoreaction between the Arctic organic matter and oxygen [167]. This result further indicates that exposure to UV radiation initiates indirect photoreactions that photo-facilitate conversion of DOM in the melting cryosphere into CO_2_ (contributes to SDGs 6.3, 6.6, 14.1). (Sect. 7.6).

Once thawed, up to 15% of the ∼1,000 Pg of organic carbon in Arctic permafrost soils may be oxidised to CO_2_ by 2100, amplifying climate change [317]. Predictions of this amplification strength are imprecise and likely are understated because they ignore the photodegradation of permafrost carbon to CO_2_ in surface waters by solar radiation. Moreover, although the permafrost zone is expected to be a substantial carbon source to the atmosphere [1], yet large-scale models currently only simulate gradual changes in seasonally thawed soil. Abrupt thaw will probably occur in < 20% of the permafrost zone but could affect half of permafrost carbon through collapsing ground, rapid erosion and landslides. After taking account of abrupt thaw stabilisation, lake drainage and soil carbon uptake by vegetation regrowth, Turetsky et al. (2020) concluded that models considering only gradual permafrost thaw are substantially underestimating carbon emissions from thawing permafrost [319].

### Thawing in the Arctic caused by climate change is releasing organic nitrogen, resulting in increased emissions of nitrous oxide, one of the major substances that deplete stratospheric ozone

As permafrost in the Arctic thaws, large carbon and nitrogen stocks are exposed for decomposition [320]. Gaseous carbon release from Arctic soils due to permafrost thawing is substantial [1], and growing evidence suggests that Arctic soils may also be relevant sources of nitrous oxide (N_2_O) [317, 320–324]. Nitrous oxide is a source gas for stratospheric nitrogen oxides and an ozone depleting substance (contributes to SDGs 13.1, 15.1); it has an almost 300 times stronger global warming potential than CO_2_ on a 100-year time horizon [322]. Oxidation by ammonia-oxidising archaea (single-celled micro-organisms) is one important source of N_2_O in Arctic peatlands [321]. Singlet oxygen, an ROS that is produced by UV interactions with Arctic DOM (Sect. 6.3), photoinhibits archaea and thus may reduce production of N_2_O [325]. Peatlands are affected by thermokarst, a marshy land surface characterised by very irregular surfaces formed as permafrost thaws; these land features are the most probable Arctic N_2_O hot spots. They cover more than 10% of the Arctic, corresponding to an area of 1.9 million km^2^. N_2_O emissions from bare soils in permafrost peatlands have been estimated to account for as much as 0.1 Tg nitrogen year^−1^ [322, 323]. This is in the range of emissions from fossil fuel combustion, industrial processes, and biomass burning, and therefore the second largest anthropogenic N_2_O sources after agriculture [322]. Measurements of Arctic N_2_O fluxes and estimates of N_2_O releases at landscape and regional scales exhibit much spatial variability so current estimates of source strengths are highly uncertain [324].

### UV radiation is likely a key driver of the breakdown of contaminants in aquatic environments

Solar UV radiation contributes to the degradation of contaminants, playing a significant role in reducing their concentration in the environment [167, 326] (contributing towards SDGs 12.4, 14.1). Microplastics are an important type of contaminant that derive from UV-initiated photoreactions of macroplastics (see assessments of interactive effects of UV radiation with materials in previous UNEP assessments [327]). Occasionally, the interactions of UV radiation with contaminants result in formation of harmful photoproducts. For example, the antimicrobial agent, triclosan, is converted by UV irradiation to toxic and carcinogenic compounds including dioxins, chloroform, and chlorinated anilines [328, 329]; and “phototoxic” contaminants, such as polycyclic aromatic hydrocarbons, become toxic on absorption of UV radiation [285, 330].

Photoreactions of contaminants occur through two general mechanisms: in the first (direct photoreaction), solar radiation directly absorbed by the contaminant results in changes in its chemical structure that can affect its persistence and fate in the environment; in the second mechanism (indirect photoreaction), the contaminant is transformed by reaction with photochemically produced reactive intermediates that are produced through absorption of solar radiation by substances such as CDOM [167, 331]. These processes usually include reactive oxygen species [332] and excited triplets of CDOM [333]. The triplets accelerate, i.e., “sensitise” photodegradation of contaminants mainly by redox reactions in which the contaminant is oxidised.

Indirect photoreactions are involved in the fate of organic contaminants [334–338], nanomaterials [339], and certain pesticides [340–342]. Considering the large effects of CDOM on the inactivation rate constants of contaminants through UV protection and photosensitisation, we expect that future ozone-related changes will be much smaller than changes caused by browning of aquatic environments. Conversely, decreases in CDOM related to drought and reduced runoff could result in much larger increases in UV-related photodegradation of contaminants than observed or projected ozone changes.

The variable effects of browning on photodegradation rates of contaminants have been assessed using a modelling approach in which three key impacts of browning on photochemistry were evaluated [343]. First, the thermocline is shifted to shallower depths, making the well-mixed surface layer (the epilimnion) shallower. Next, the higher concentrations of CDOM lead to lower average light intensities. Finally, the higher CDOM concentrations lead to formation of higher concentrations of photochemically produced reactive intermediates. As these three effects do not all point in the same direction with respect to contaminant degradation, the authors used a modelling approach to resolve the net effect. The results indicate that direct photodegradation processes would be slowed by browning, while reactions with triplet state organic matter and singlet oxygen would be enhanced. Vertical hydrodynamic mixing also was shown to affect photomineralisation of dissolved organic carbon in arctic waters [344]. The overall effect of lake browning on contaminant degradation is therefore not expected to be uniform, but rather dependent on the specific molecule of interest and its dominant photochemical pathways.

We caution that the equations and data used in the simulations by Calderaro and Vione (2020) do not consider the effects of suspended particulate matter on absorbance and scattering in the water. Suspended particulate matter can account for a significant fraction of absorbance of UV radiation in the Great Lakes, estuaries, and other water bodies [345, 346].

Thus, solar UV radiation generally initiates the photodegradation of contaminants in aquatic environments by direct and indirect processes that variably depend on changes in stratospheric ozone. Implementation of the Montreal Protocol [294] has reduced direct photodegradation rates of UV-B-absorbing contaminants. On the other hand, indirect photodegradation is enhanced by browning and thus has been accelerated by climate-related factors such as runoff of CDOM driven by rainfall or melting of ice and permafrost.

### Indirect photoreactions enhance the photolysis of certain contaminants, especially persistent organic pollutants

Indirect photoreactions are a particularly dominant type of solar radiation-induced transformation for contaminants that undergo direct photolysis very slowly [167, 331, 333]. Persistent organic pollutants (POPs) often fall into this category. POPs are chemicals that are resistant to environmental degradation, accumulating through food chains and are harmful/toxic to humans and wildlife [347] (contributes to SDGs 6.3, 6.6, 14.1). The indirect pathway for the photolysis of POPs is particularly relevant, since some of these contaminants (e.g., perfluorinated octanoic acid (PFOA) and some pesticides) do not absorb significantly within the UV region of the solar spectrum and therefore undergo direct photoreaction very slowly. In natural waters, they may be potentially transformed/degraded via indirect photolysis mediated by natural sensitisers (e.g., DOM, nitrate or nitrite) that absorb solar radiation to form photochemically produced reactive intermediates [329, 334, 348, 349].

The effects of the naturally occurring substances such as CDOM on degradation of POPs are complex and can either accelerate or inhibit photolysis of these contaminants. Also, other contaminants enter the aquatic environment, such as engineered nanomaterials and their composites with polymers [350], which are a rapidly growing class of emerging contaminants that can be transformed by photoreactions [167]. Models have been developed to simulate the environmental fate of these nanomaterials in aquatic systems [351].

### Photoinactivation by solar UV radiation mitigates health-related problems associated with exposure to viruses and other biological contaminants in aquatic environments

Viruses are thought to be responsible for most gastrointestinal (GI) illnesses contracted in recreational waters contaminated by human faeces [352, 353]. Representative human sewage-borne viruses include enteroviruses, noroviruses, and adenoviruses. Bacteria and protozoans are other classes of pathogens that are present in aquatic environments (contributes to SDGs 3.3, 6.3).

Pathogen indicators are microorganisms often used to assess the presence of pathogens in recreational waters. The term “indicators” is widely used to refer to microorganisms that indicate the presence of faecal contamination in water (e.g., bacteria such as *E. coli*). Biological weighting functions are used to quantify wavelength effects on direct photoinactivation of microorganisms and to better understand the role of microbial characteristics and environmental changes in their sensitivity to UV radiation [289, 354, 355]. Models using biological weighting functions have been applied to assess water quality. For example, the effects of attenuation of solar UV radiation on photoinactivation of pathogen indicators in various swimming areas of the Great Lakes has been assessed using models that integrate the biological weighting functions and other data, including attenuation coefficients and transport data [356, 357].

## Air quality

UV radiation is a major contributor to the formation of urban and continental-scale air pollution. Many organic compounds emitted because of human activities and natural processes are transformed (photo-oxidised) by solar UV radiation into products that are, in many cases, more toxic than the original substance released. These products include ambient ozone (O_3_), carbon monoxide (CO), organics such as formaldehyde (CH_2_O), and aerosols—hazes of suspended particles, with those smaller than about 2.5 μm (PM2.5) being particularly problematic. Sulfate, nitrate, and secondary organic aerosols (SOA) are formed directly from this UV photochemistry. Air pollution has been identified as a critical issue in human health. According to the World Health Organization, ambient air pollution contributed to 7.6% of all deaths in 2016 [358]. The importance of improving air quality is recognised in the SDGs 3.9 and 11.6. Air quality is dependent on solar UV radiation in the troposphere and thus on stratospheric ozone. It is also influenced by atmospheric circulation, including transport of ozone from the stratosphere to the troposphere, which can be altered by changes in climate and as a result of ODS [37]. Understanding how air quality depends on changes in UV radiation, whether from depletion of ozone or other causes (e.g., aerosols), remains a fundamental challenge to the prediction of air quality.

**Fig. 12:**
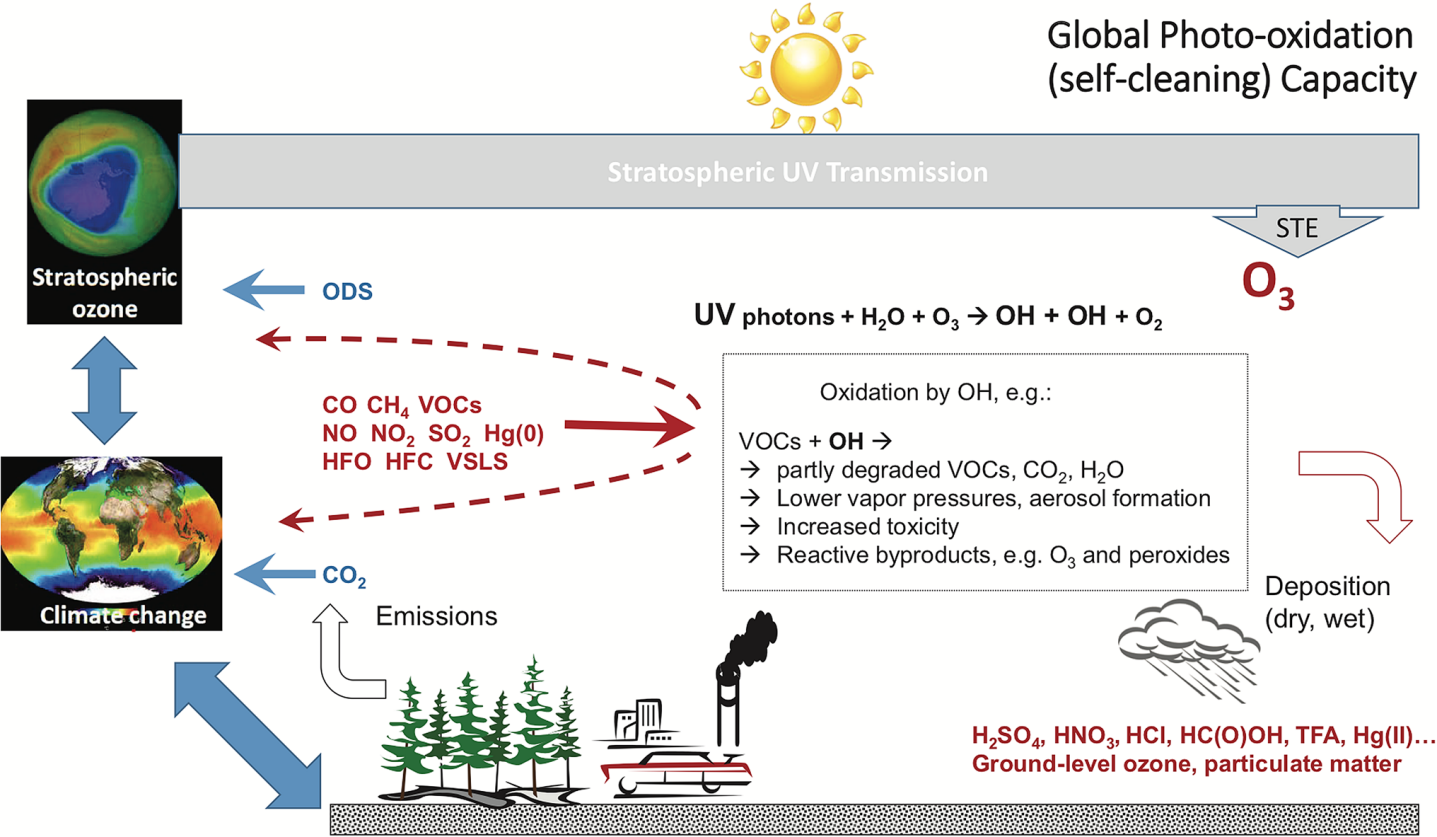
Central role of UV-driven oxidation processes in the chemistry of the troposphere. Besides controlling the amount of UV radiation reaching the troposphere, stratospheric ozone (O_3_) is also a major source of tropospheric ozone via Stratosphere–Troposphere Exchange (STE). The rate of removal of many tropospheric pollutants [including volatile organic compounds (VOCs)] is determined by reaction with OH

The hydroxyl radical (OH) is the major oxidant of tropospheric pollutants and is central to the regulation of ambient air quality. OH is primarily produced by the absorption of UV-B radiation by ozone (Fig. 12) and the concentration of OH determines the persistence of many pollutants in the atmosphere. OH is usually destroyed when it reacts with pollutants, so that the actual concentration of OH is a balance between UV-B-driven production and its reaction with pollutants. Climate change may enhance the emission of tropospheric pollutants, like CO, from natural sources and this would reduce the concentration of OH and increase the persistence of other chemicals, including the greenhouse gas (GHG), methane (CH_4_), and very short-lived halogen compounds (VSLS), such as bromoform (CHBr_3_) that could increase destruction of stratospheric ozone and change concentrations of tropospheric ozone [359] (Fig. 12). Changes in concentrations of reactive halogens and ozone can also have impacts on the movement of mercury through the environment [360].

Currently, large systematic changes in air pollution and surface UV radiation are occurring due to (a) long-term reductions in emissions arising because of legislated air quality control strategies (e.g., in China over the past few decades), and (b) shorter-term reductions in many countries, from the economic slowdown associated with the COVID-19 pandemic (Sect. 9). The points below update our current understanding of changes in air pollution and the interactive effects of stratospheric ozone, UV radiation, and the transport of air from the stratosphere to the troposphere.

### Climate change and stratospheric ozone recovery are expected to increase ozone in the troposphere globally, but there remains significant uncertainty as to the impact of a range of processes

**Fig. 13:**
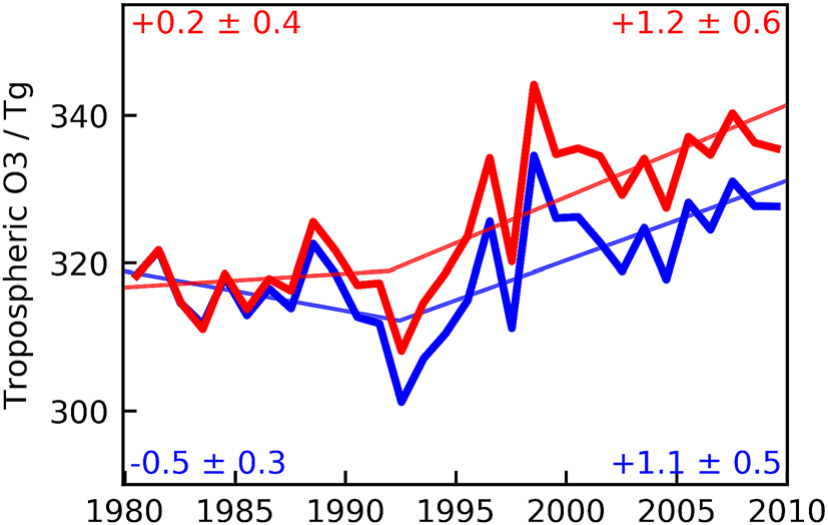
Calculated mass of ozone transported from the stratosphere to the troposphere (STE) 1980–2010 in Tg/year. The blue curve is based on an estimate of the actual state of the atmosphere. The red curve is what would have happened if halogenated ODS emissions remained at 1979 levels. The numbers quoted are the slopes of the trend lines. Figure kindly supplied by M. Shin [361]

The concentration of tropospheric ozone in any location is dependent on large-scale sources (e.g., ozone transported to the troposphere from the stratosphere or from other continents) and production from local emissions. Reductions in stratospheric ozone have decreased the amount of ozone transported to the troposphere in the last 30 years [361]. Figure 13 highlights the reduction in transported ozone because of enhanced emissions of ODS up until 1994 (the difference between the two curves). After 1994, changes in atmospheric circulation have increased the annual flux of ozone from the stratosphere to the troposphere. These changes in circulation are a result of the increase in concentration of GHGs, and the trends are likely to continue.

As the climate warms, the removal of ozone from the atmosphere at Earth’s surface may diminish. However, the dominant processes for removal of ozone (e.g., uptake by leaves and/or the soil, or reaction with volatile organic compounds [VOCs] such as terpenes released by plants into the atmosphere) appear to differ between locations [362, 363]. Dissolution into the ocean is a significant process for removing ozone from the atmosphere but has probably been overestimated by a factor of 2 [364]. Correcting this overestimate increases model estimates of concentrations of surface ozone by 10% or more for a third of the surface of Earth. Tropospheric ozone is an important marker for air pollution that is not just a national issue, since ozone from ground-based sources circulates around the globe [365].

### The effect of UV radiation on the atmospheric chemistry and lifetime of aerosols is larger than previously recognised

It is well established that UV radiation is central to the formation of many aerosols in the atmosphere, such as those containing sulfate, nitrate, and many organic compounds. These so-called secondary organic aerosols (SOA) are formed in the atmosphere through the oxidation of VOCs that form low volatility products (Fig. 12). New research indicates that UV radiation also plays a role in the destruction of some of these particles. Measurements in the laboratory show that many SOA are photodegraded by UV irradiation [366], making UV radiation an essential factor in both their production and removal from the atmosphere. According to model calculations [367], these complex UV-driven processes could represent the largest global sink for SOA, exceeding removal by rain-out (Fig. 12). However, a fraction of SOA appears not to be degraded by UV radiation [366, 368, 369], with the overall effect of the UV radiation depending on the chemical composition of the many different aerosols present in the atmosphere. No attempt has yet been made to quantify the sensitivity of the lifetimes of different SOA to changes in UV radiation as would occur with changes in stratospheric ozone.

### UV radiation induces chemical transformations on the surface of and within aerosol particles, producing chemical species potentially damaging to air quality and health

Heterogeneous photochemistry of aerosol particles has attracted increasing interest in the past decade due to its potential to modify the chemical composition, toxicity, and optical properties of aerosols [e.g., [370, 371]]. Recently, laboratory studies showed that SOA exposed to UV radiation can act as photo-sensitisers for the oxidation of gaseous sulfur dioxide (SO_2_) to condensable sulfate [372], thus potentially accelerating the formation of sulfate aerosols. This occurs when UV radiation is absorbed by organic particles and produces highly reactive intermediate molecules that rapidly oxidise SO_2_ gas associated with the particles. The importance of these reactions to the Earth’s atmosphere remains to be established. Similar UV-induced excited-state chemistry in organic aerosols was also found to lead to the formation of reactive oxygen species such as peroxides [373], which are postulated to cause significant damage to the respiratory tract upon inhalation [374–376]. Thus, UV radiation may modify the impact of organic aerosols on human health and would represent an additional pathway for changes in stratospheric ozone to influence human health.

### Efforts to reduce emissions of fine particles in polluted regions have resulted in undesirable increases in concentrations of ambient ozone in some regions

In China, long-term reductions in some emissions [e.g., nitrogen oxides (NO_*x*_) and SO_2_] have resulted in substantial reductions in aerosol haze, but this has been accompanied by an undesirable increase in ambient ozone [377–380]. Observations show worsening ozone pollution between 2013 and 2017 at urban locations in China [379, 381, 382] and in the North China Plain from 2013 to 2019 [383]. Possible reasons include: fewer ozone-destroying chemical reactions occurring on aerosol particles [383, 384]; lower NO_x_/VOC ratios leading to higher ozone production efficiencies [382, 383]; increase in emissions of VOCs due to changes in temperature [384]; and increases in UV radiation due to less haze [378, 380, 383–387]. The relative importance of these factors remains uncertain and depends on the formulation of models, including detailed changes in emissions, parameterisation of the complex chemistry, and spatial resolution of models. This highlights the importance of solar UV radiation in determining the composition of air pollution, although quantification remains elusive. The changes in ozone also demonstrate the difficulties of managing multi-component air pollution.

Similar changes in air quality have been observed around the globe as a result of the economic slowdown associated with the COVID-19 pandemic (Sect. 9.7). The sharp reductions in emissions will provide comprehensive data to evaluate and improve air quality models [388, 389], taking advantage of modern experimental techniques and platforms for collection of data.

### Droughts and other effects of climate change are expected to enhance emissions of carbon monoxide from wildfires, thus decreasing concentrations of the hydroxyl radical and increasing the lifetime of methane

As a result of reduced anthropogenic emissions of CO from the United States, Europe, and China, a decline in the global concentration of CO since 2000 has been reported [390]. However, climate change may enhance emissions of CO from natural sources. For example, a major biogenic source of CO to the atmosphere is the emission of volatile organic compounds, such as isoprene from plants, with CO being formed in chemical reactions in the atmosphere. These biogenic emissions account for about 18% of the global burden of atmospheric CO [391]. Wildfires are another natural source of CO to the atmosphere, particularly in boreal forests [392] that occur as a consequence of climate change-driven extreme hot and dry conditions in some parts of the Earth. For example, satellite-derived rates of CO emissions during the Horse River wildfire in 2016 were ~ 50–300 ktonne/day and exceeded the total annual anthropogenic emissions of CO in Alberta that year [392]. Overall, although total CO emissions have declined since 2000, climate change may enhance emissions of CO from terrestrial and aquatic ecosystems. The prediction of atmospheric trends in CO is important, since CO is not only a tropospheric pollutant, but also a sink of OH and thus affects the lifetime of the GHG, methane [1, 393–395]. It has been estimated that, during the 1997–1998 El Niño event, the global atmospheric concentration of CO increased by more than 40%, caused by wildfires, concomitant with a decrease in global mass-weighted tropospheric concentration of OH up to 9% and, as a consequence, a 4% increase in the lifetime of atmospheric CH_4_ [396].

### Enhanced thawing due to climate change of permafrost soils, sub-sea permafrost, and methane hydrates has the potential to release large quantities of methane with likely long-term impacts on hydroxyl radical concentrations and the global photo-oxidation capacity

Methane contributes directly to the chemical loss of stratospheric ozone, and also indirectly (like other GHGs) by warming the troposphere and cooling the stratosphere [397, 398]. Natural sources of CH_4_ are likely to increase in the future because of climate change. Among other natural sources, Arctic ecosystems are large, yet poorly understood sources of CH_4_ and are undergoing rapid change, including melting of sea-ice, thawing of sub-sea permafrost, methane-hydrates, and permafrost soils with the formation of thermokast lakes [399–404] (Sect. 6.4). As a consequence of thawing of permafrost, centuries-old carbon deposits become bioavailable and undergo microbial mineralisation [404]. Rates of emission of CH_4_ from Arctic and other natural environments depend on rates of methane production and consumption by methane-producing and methane-oxidising microorganisms, respectively, which are affected by temperature and other environmental factors [405, 406]. Methane plays a critical role in determining tropospheric concentrations of OH (more CH_4_, less OH) and hence the tropospheric cleaning capacity (Sect. 7.5).

### Emissions of halogen-containing very short-lived substances are increasing and they act as a source of reactive halogen species in the troposphere and stratosphere

Atmospheric concentrations of anthropogenic halogen-containing very short-lived substances (VSLSs) have been increasing [407] and biogenic VSLSs are predicted to increase due to climate change [407, 408]. For example, emissions of CHBr_3_ from the ocean are projected to increase by 31% during 2010–2100 under a scenario of high emissions of GHGs [407]. Anthropogenic and biogenic VSLSs are not controlled by the Montreal Protocol but they can reach the stratosphere where they form reactive halogen species that contribute to the depletion of ozone [407, 409–412]. The contribution of chlorinated VSLS to total stratospheric chlorine increased from ~ 2% in 2000 to ~ 3.4% in 2017, reflecting the growth in the release of VSLS and decreases in concentrations of long-lived halocarbons [409]. The contribution of very short-lived bromocarbons to the total stratospheric bromine loading is higher (~ 25%) [412]. In the troposphere, the oxidation of VSLSs by OH yields reactive halogen species that act as oxidants of tropospheric pollutants, e.g., mercury and ozone [359]. Reactive halogen species produced from natural VSLSs deplete approximately 13% of tropospheric ozone in the present-day climate [359]. Hence reactive halogen species decrease concentrations of ozone in both the troposphere and the stratosphere.

### Trifluoroacetic acid continues to be found in the environment, including in remote regions, although not at concentrations likely to have adverse toxicological consequences

Trifluoroacetic acid (TFA) is found in the environment as a salt, with a no-observed-effect-concentration (NOEC) for aquatic species, which is typically > 10,000 μg/L [413]. TFA is produced by the environmental degradation of several hydrofluorocarbons (HFCs) and hydrofluoroolefins (HFOs) (Fig. 12). Analysis of 1187 samples of rainwater collected in eight locations across Germany in 2018–2019 [414] showed median and a precipitation-weighted mean concentration of TFA of 0.210 μg/L and 0.335 μg/L, respectively. The maximum measured concentration was 57 μg/L. The authors reported a seasonal variation in flux of TFA with greater deposition in the summer than winter. The flux was significantly correlated with global intensity of solar radiation, indicating that the sunlight-produced OH contributed to the degradation of the precursors of TFA, most likely refrigerants released to the atmosphere. Another recent paper reported TFA [and other perfluoroalkyl substances with > 2 carbons (PFASs)^4^] in precipitation in the low μg/L range across 28 cities in mainland China [415]. The spatial and temporal variation in concentrations of TFA was large (0.0091–1.8 μg/L) but these results are consistent with earlier observations included in the EEAP’s quadrennial assessments [416–421].

Residues of TFA and other short-chain (C3–C4) PFASs have also been measured in Arctic snow cores [422]. See Online Resource (Supplementary Material 1), Table 1S, for information on toxicity of PFASs with > 2 carbons). Ice cores were collected from two high-altitude locations in Nunavut: the Devon ice cap and the Mt. Oxford icefield. These cores were dated from 1977 to 2015 and 1967 to 2017, respectively, and provided a good historical record of deposition of TFA in relation to the switch from hydrochlorofluorocarbons, which do not break down to form TFA, to HFC and HFOs, which do. Trends of concentrations with depth in the cores were similar in the two locations. The maximum concentrations of TFA in the ice cores were less than the average concentration reported in marine waters [423]. Thus, even the complete melting of the upper 15 m of the Arctic ice cap would not increase concentrations of TFA in marine waters. Instead, it would dilute the concentration of TFA in the oceans after complete mixing. Seasonal distribution of TFA and other PFASs in the ice-cores were not reported but might be expected to be greater in summer than winter as reported above for rainfall [414].

### Other sources of TFA, besides from refrigerants and propellants that fall under the purview of the Montreal Protocol, may be more important but less understood

Fugitive emissions of TFA have been reported from landfills, transfer stations, and incinerators in locations where manufacturing facilities produce fluorinated chemicals [1]. In a study by Wang and colleagues [424], air samples and water leaching from a location that received municipal solid waste near the city of Tianjin in China were analysed for PFAS. Air samples contained 20 longer chain PFAS, but dominant compounds were those with 8, 10 and 12 carbons in the chain. TFA, which is not volatile, was not detected in air. Leachates contained 19 PFAS with carbon chains from 2 to 10 in length with the dominant compound being TFA. Whether the compounds with shorter chain lengths were breakdown products of longer chain compounds is not known. Concentrations of TFA in leachates and effluents from waste disposal sites were large; no TFA was detected at one site but the median concentration of TFA in the other ten sites was 17 μg/L with a range across sites of 2–59 μg/L [424]. In another study, the distribution of TFA was measured in the surroundings of Jinan, the capital of Shandong Province, China, which is a region with three fluorochemical manufacturing facilities [425]. Concentrations in river water ranged from 0.5 to 1.1 μg/L, spring water had a mean concentration of 1.8 μg/L, lake water 2.7 μg/L, and tap and well water, 0.25 μg/L. These large concentrations are indicative of a local source and these industrial sources are not tracked in the same way as for those compounds controlled by the Montreal Protocol. Presently it is not possible to even estimate their contribution to the global amount. However, if concentrations of TFA in oceans begin to increase, the total emissions could be estimated and hence the contribution of these other sources could be better estimated in relation to the refrigerants and propellants.

### Current concentrations of TFA salts and related compounds in soil and surface waters do not present risks of adverse effects in aquatic and terrestrial plants and animals

Historical and current measurements of TFA in soil and surface-water indicate *de minimis* risks when compared to no-effect-concentrations (NOECs) in laboratory and field-based testing [413]. Neither of two new studies on uptake of salts of TFA by plants reported adverse effects on wheat, maize, and poplar [426, 427]. The measured concentration of TFA in the shoots of wheat (116 mg/kg, estimated to be equivalent to 116 × 10^3^ μg/L) [427], is ten-times greater than the NOEC for aquatic plants measured under field conditions [428]. This indicates that terrestrial plants are likely less sensitive to TFA than aquatic plants. The median concentrations of TFA reported in ice cores from the Devon and Mt. Oxford ice caps were 0.021 μg/L and 0.009 μg/L, respectively, and the maximum measured in both sites was 0.15 μg/L [422]. Compared to the NOEC for aquatic plants, this provides a margin of exposure of 570,000 for the greater median and 80,000 for the maximum value. Although plants would not be expected to grow in these cold locations, this indicates that these concentrations pose a *de minimis* risk to aquatic and other plants and animals as well.

It should be noted that Pickard et al. [422] erroneously claimed that TFA was toxic to plants and cited a review [413] in support of this statement. This was not the conclusion in the review, but to compound the error, the press release from the University of Edmonton [429] further propagated incorrect information when it quoted the primary author as stating that “some have been found highly toxic to plants”, presumably referring to TFA as they present no toxicity data on the other compounds they found in the cores. There is no scientific basis for this conclusion and risks from current and future releases of TFA from the use of fluorinated precursors regulated under the Montreal Protocol to aquatic and terrestrial plants are *de minimis* [1, 413, 421].

### Humans could be exposed to TFA via drinking water and food but there is no evidence to date of adverse effects on health

TFA salts are of low acute toxicity to mammals under conditions relevant to environmental exposure (reviewed in ref. [413]). However, TFA has been found in the blood of humans [430]. In this study, serum samples were collected from 252 participants from the staff and support workers at Nankai University in Tianjin, China, a location close to chemical plants manufacturing fluorinated chemicals. Twenty-one PFASs, including TFA were quantified in serum. Two markers of glycemia were also measured in these participants to examine potential linkages between exposures to PFASs and disease. TFA was detected in 97% of samples with a median concentration of 8.46 μg/L. Concentrations were significantly elevated (*P* < 0.05) in participants > 40 years old and TFA accounted for 17.2% (w/w) of the total PFASs. When data were adjusted for potential confounders, no association (*P* = 0.09) was observed for concentration of TFA in serum and fasting glucose levels or HbA1C (a marker for diabetes) in serum (*P* = 0.1). However, significant associations were observed for several other longer-chain PFASs. This study appears to have been the first to measure TFA in the blood of humans in relation to environmental exposures, so it is not possible to compare these results with those from other locations. The source of exposure to TFA was not identified but others have reported the presence of PFASs in the area but did not include TFA as an analyte [431].

It is possible that the TFA observed in human blood was not only due to direct exposure to TFA, but also the result of the metabolic breakdown of one or more of the longer-chain PFASs. As discussed above, measurements of TFA in leachates and effluents from waste disposal sites in Tainjin reported concentrations of TFA of 2–59 μg/L [424], suggesting possible sources of contamination from ground and surface water. The concentrations in serum are greater than one would expect for a polar compound that is readily excreted by mammals. TFA is also formed from the metabolism of the anaesthetics halothane and desfluorane in humans. The half-life for excretion of TFA from the metabolism of anaesthetics in humans was reported to be 16 h [432]. Others have shown similar rates of excretion in adults [433] and children [434] where half-lives of TFA ranged from 10–59 h. In the study on metabolism of desfluorane in humans [433], the mean maximum concentration of TFA in serum was 43 ± 19 μg/L, which is *ca* fivefold greater than the median concentration reported in the Duan et al. study [430]. Further studies in Tianjin and other locations would help illuminate the unexpected finding of large environmental exposures and/or apparent accumulation of TFA in humans.

## Material damage

A wide range of materials that are routinely exposed to solar UV radiation during use, undergo slow photodegradation resulting in a gradual loss of their useful properties. Climate change can alter the weathering conditions outdoors affecting the rates of degradation and therefore fragmentation of materials. These materials include wood, plastics, textile fibres, and organic coatings, where the damage can range from aesthetic changes such as discolouration to surface cracking that affects the mechanical integrity of the material. Often, the outdoor service lives of systems and components that incorporate these materials (e.g., photovoltaic panels, building materials, and outdoor furniture) are determined by their rate of degradation under solar UV radiation exposure. Solar UV-induced damage can generally be controlled by the judicious selection of UV-resistant materials for outdoor use, using high-efficiency light stabilisers in plastics and employing surface coatings on wood that absorb or scatter the UV radiation. Emerging technologies suggest the use of nanoscale UV-absorbing fillers to be particularly effective additives in this regard. An important application of these nanoscale fillers is in textile fibres to obtain fabrics with very high ultraviolet protection factors for use in protective apparel.

**Fig. 14:**
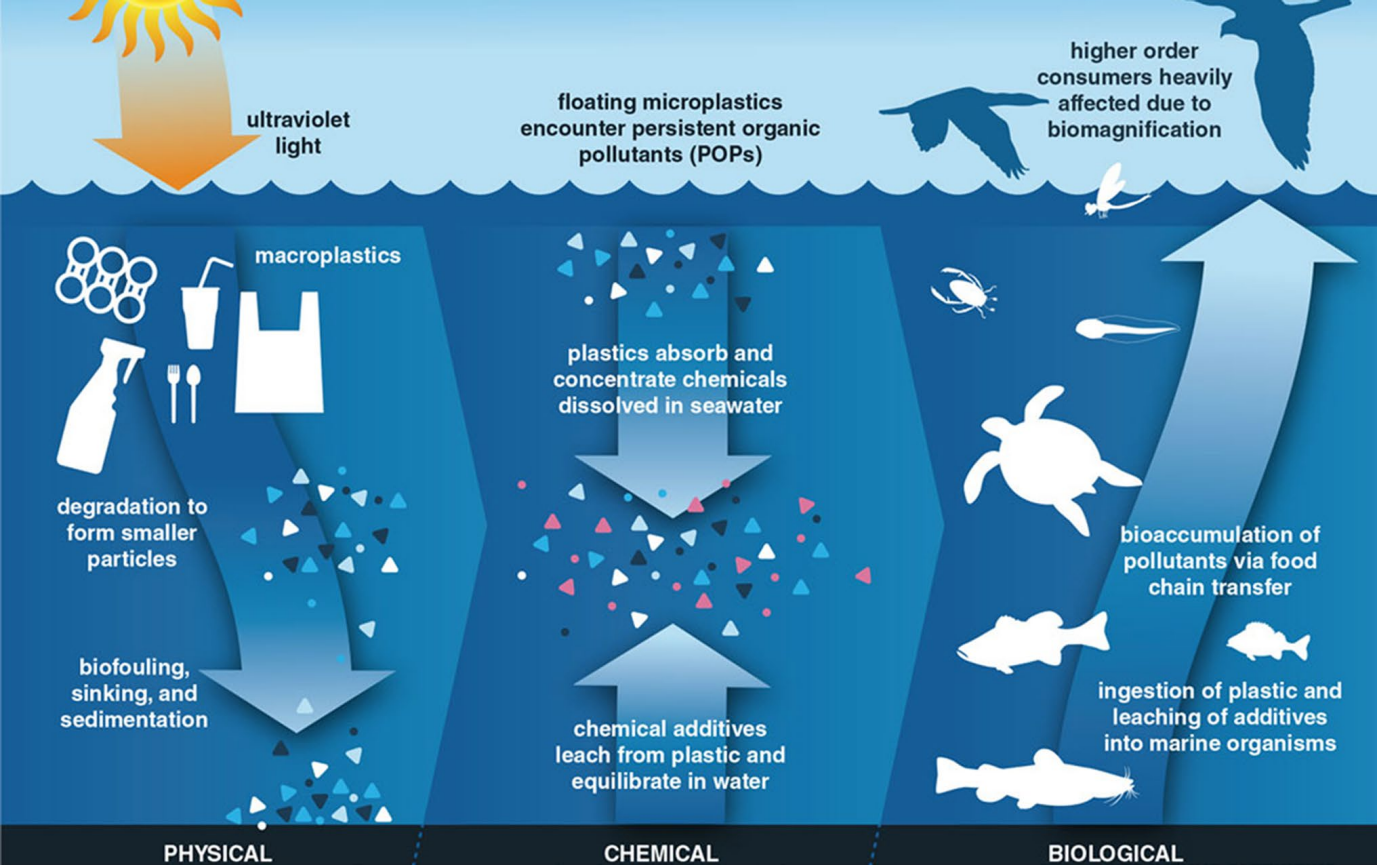
Physical, chemical and biological processes associated with plastics in the ocean environment. Courtesy of source [435]

Sustainability considerations (SDG 17.14) in the plastics industry encourage the use of new ‘green’ additives, including biomass-derived stabilisers, in both polymer technology and in wood coatings. How these novel’green’ additives will affect the UV stability of the plastics matrix, or the coatings they are compounded into, is still under investigation. A consequence of extensive degradation by solar UV radiation of plastic litter is the generation of microplastics, a fraction of which enters marine biomass via ingestion, affecting the health of the ecosystem (Fig. 14; SDGs 6.3, 12.4–5, 14.1). Studying the formation of microplastics and developing strategies to reduce their abundance in the environment, especially the marine ecosystem, is a high research priority and is ongoing.

This section assesses recent research in the above-mentioned areas especially focusing on emerging mitigation technologies and investigations that are relevant to the commitments of the Montreal Protocol and its Amendments. Special emphasis is being placed on degradation by solar UV radiation and stabilisation of materials.

### Wood extractives provide promising sustainable alternatives to conventional UV-stabilisers in coatings and wood-plastic composites

Concerns about sustainability have encouraged the use of ‘green’ UV stabilisers in both wood and plastics products, seeking to reduce the toxicity associated with conventional stabilisers that invariably contaminate the environment. The use of bark and wood extracts as well as lignins as UV stabilisers is a trend that has been recently reported [436–439]. New research confirms the general phenomenon and extends the finding to several additional wood species. Wood bark extracts are rich in polyphenols that include condensed tannins and flavonoids, which are particularly good absorbers of UV radiation and free radical scavengers. Scots pine (*Pinus sylvestris* L.) wood was successfully stabilised with bark extracts from Chinese fir (*Cunninghamia lanceolata*), used at 2 wt% level in polyurethane-acrylate wood coatings [440]. Upon accelerated exposure with UV radiation of up to 960 h, coatings with bark extractives using ethanol reduced the discolouration to about 65% of that for the control wood surfaces. A 1 wt % solution of the extractives of *Phoebe zhennan* wood with polar solvents completely absorbed solar UV-A and UV-B radiation in laboratory studies [441]. UV-shielding films of poly(lactic acid) with 24 wt% of wood extract screened out all of UV-B and 80% of UV-A radiation. Several bark extract stabilisers of alder and pine species at a concentration of 5% in alkyd-based coatings on Scotts pine were compared with commercial UV stabilisers including triazine UV stabilisers and hindered amine light stabilisers (HALS) [442]. At over 2000 h of laboratory weathering the best protection was afforded by the natural stabilisers.

These stabilisers can also be used with wood–plastic composites that undergo similar UV-induced discolouration and loss of mechanical integrity on exposure to solar UV radiation [443]. Bark extracts from western red cedar at 2 wt% mixed into the wood–plastic composite yielded about 20% less discolouration along with less severe surface cracking and chemical degradation as indicated by Fourier-transform infrared spectroscopy after 1200 h of accelerated weathering [444]. In both wood and wood–plastic composites, the trend towards switching to sustainable ‘green’ UV stabilisers has significant environmental value if also economically feasible.

### Nano-scale lignins from a sustainable source, derived from agricultural waste, serve as UV-shielding additives in textile fibre coatings and in plastic films

Lignins in wood species are structurally rich in chromophores that absorb incident solar UV radiation in the wavelength range 290–400 nm and can, therefore, effectively function as UV-absorbers in UV-protective polymer films [445, 446]. The detailed chemistry of the phenolic composition of lignins, however, is species specific and the research findings cannot be generalised for lignins derived from different biomass sources. Even aqueous suspensions of lignin and clay [445], or gelatin [447, 448] without any added synthetic polymer can be formed into excellent UV protective biofilms.

The efficacy of lignins derived from waste biomass as UV-protective agents in textile applications has been reported. Extracted from groundnut shell waste, the lignins applied to natural wool fibre surface at 5 wt% level increased the Ultraviolet Protection Factor (UPF) of the native fibre from ca 6 to 700–1650 [449]. Coconut shell-derived lignin, when used at the same wt. fraction in acrylic polymer films reduced their UV transmission from 45% to as low as 0.8%, but with associated loss in transparency from 90 to 26% [450]. Nanoscale lignins are especially superior UV-absorbers because of their high specific surface area. Cotton stalk waste, after partial microbial degradation, yielded ~ 45 wt.% of nano-lignin that was used as coatings on cotton and linen fibres to enhance their UV stability. The coatings increased the native UPF value (~ 30 for cotton and ~ 25 for linen) by a factor of *ca* 5 [451]. A high UPF was also obtained for poly(vinyl alcohol) (PVA) nanocomposite films crosslinked with a 50% loading of lignin [452].

With the growing demand for UV-protective fibre worldwide, lignins can serve as a sustainable textile coating material. While lignin is considered a cost-effective and green UV stabiliser, with its chemistry being species-dependent, large-scale use requires standardisation of nanolignin composition [453] and sourcing of biomass feedstock with consistent quality.

### Plasma treatment may reverse the damaging effects of UV radiation in wood

Using atmospheric-pressure plasma (APP) to modify wood surfaces is well known to increase the affinity of the surface to water (hydrophilicity) by increasing the oxygen to carbon ratio (O:C) in the treated wood as evidenced by X-ray photoelectron spectral studies [454]. This increase in the [O:C] ratio linearly correlates with a higher surface free energy of thermally modified wood species including alder, dark alder, ash, and aspen [455]. Because of increased polarity, APP treatment results in better adhesion properties of water-based coatings, for instance, on beech wood [456] and functional glaze or lacquer coating on larch, Douglas fir, and Scots pine wood [457].

Wood exposed to solar UV radiation typically undergoes surface discolouration and degradation that reduces its adhesive strength for coatings [458, 459]. A novel variant of the APP technology was shown to successfully reverse moderate effects of surface degradation due to exposure to UV radiation of *Fagus sylvatica* (beech wood) [460]. Beech wood weathered under exposure to a xenon source in the laboratory, when subsequently treated by the novel variant of APP, showed an increase of adhesion strength by up to 20%. Treated, pre-weathered sample surfaces showed faster spreading kinetics and a doubling of the spreading area for water, due to increased surface free energy from the plasma treatment. However, the success of the technique is dependent on the variety of wood and for instance, did not work with spruce wood. Also, with plasma treatment of wood the consequent hydrophilicity may encourage surface biofilm formation [461], which in some varieties of wood leads to premature biodegradation. Being an emerging technology the efficacy of modified APP has yet to be validated in a large number of common wood species and the trade-off in terms of any contribution for biodegradation has still to be assessed. This is a significant development if the approach can cost-effectively improve weathered wood and thereby reduce the impact of solar UV radiation on the lifetimes of wood products used in the building industry.

### Nanoparticles attached to the surface of textile fibres yield efficient UV-protective fabrics

A range of nanoparticles are applied either directly to the surface of textile fibres or used in fibre coatings to increase their UV-shielding properties. Most continuing current research on this approach is focused on nanoparticles of zinc oxide (ZnO) [462–464] and titanium dioxide (TiO_2_) [465–469]. All reported data confirm the efficacy of this approach in increasing UPF of fabrics. Often, the nanoparticles used are selected for other improvements in fibre quality, such as better antibacterial properties or electrical conductivity, thus creating a multi-functional fabric [469–472]. Other metal oxides, such as NiOx nanoparticles in gas-sensing cotton fibres [473] and SiOx nanoparticles in superhydrophobic cotton [474, 475], are used to obtain specific functionalities, although they also improve UV-shielding properties. Graphene oxide [476] and multi-walled carbon nanotubes [477] have also been investigated in this regard.

The optical bandgap of ZnO nanoparticles (26 nm) is particle-geometry dependent and rod- shaped-particles show the highest value compared to spherical or star-shaped nanoparticles. Nanorods of ZnO absorb incident solar UV-A as well as UV-B radiation [464]. For cotton and polyester, coating the fibre with the nanorods resulted in UPF values of 247. Increased attention needs to be paid to the shape factor to optimise resultant UV-protection, especially at lower nanoparticle concentrations. Rather than decorating the surface of fibres with particles of ZnO, the nanoparticles were uniquely grown in situ on cotton fibres after polymer brushes were grafted onto the cotton [478]. This yielded nanoparticles on both the outer and inner surface of the hollow cotton fibres. The nanoparticles provided very good protection from UV radiation, with the value of UPF for the corresponding fabric reaching > 350. While UPF values as low as 50 are regarded as providing excellent UV-shielding, high values are still desirable, since the UPF values of fabrics tend to drop with laundering cycles.

### Using thermoplastic polyolefin as the encapsulant in solar photovoltaic modules can increase their service life

A majority of failure in photovoltaic (PV) modules involve encapsulant degradation, yellowing of back sheets or de-bonding of elements due to adhesive failure [479]. In a cross-sectional study of a weathered adhered laminate sandwich construction of a PV module (glass/encapsulate/back sheet), using spatially resolved fluorescence imaging, damage from UV radiation to the adhesive layers was disproportionately high [480]. Copolymers of ethylene and vinyl acetate (EVA) are popularly used as encapsulants in solar PV modules. Accelerated weathering studies on several different EVA copolymers found those with 18–33% by weight in copolymer vinyl acetate to be the most stable to solar UV radiation [481]. The encapsulant degradation of EVA by solar UV radiation, as assessed by FTIR, was found to be inhomogeneous, suggesting that intrusion of oxygen and moisture from the edges into the interior of laminates governed their degradation process [482]. Advances in PV technology can benefit from more stable adhesives and encapsulants (SDG 7.A).

The use of thermoplastic polyolefin (TPO) as an alternative to EVA as the encapsulant has been evaluated [483, 484] and was found to have superior thermal stability, adhesive strength and resistance to discolouration. For instance, discolouration on exposure to UV radiation was *ca* nine times slower in TPO relative to EVA, and may also be easier to process. While the initial research on TPO is promising, developmental work needs to further validate this approach prior to commercialisation.

### In polymer photodegradation, damage varies linearly with the dose of radiation for only some of the modes of damage

Evaluations of laboratory-accelerated weathering have been routinely used to assess outdoor degradation rates of common plastics such as polyethylene (PE) [485], polypropylene (PP) [486, 487], polycarbonates (PC) [488] and polyesters (PET) [489, 490]. These weathering procedures rely on elevated temperature and higher light intensity relative to natural exposures. However, with several other variables such as humidity, air pollutants and abrasion by sand acting in concert to also influence degradation rates in the field, data from laboratory tests do not always correlate well with outdoor exposure data.

In such studies, photo-damage is assumed to be a linear function of the dose of radiation (or the product of intensity and duration of exposure). Where higher levels of UV radiation are used for shorter durations in laboratory-accelerated exposures, ensuring that this linearity holds is important. With polycarbonate (PC), poly butylene terephthalate (PBT) and styrene-acrylonitrile (SAN), the accelerated-exposure studies do show that the selected properties change linearly with the dose of radiation [488]. Other polymers such as high-density polyethylene (HDPE), however, show a dependence of UV radiation dose on the increase in surface crystallinity, but not on changes in bulk strength or stiffness [485]. The same lack of a linear dependence for some of the properties was reported for photodamage of poly (ethylene terephthalate) [PET] [489, 490] as well as for HDPE [491] in accelerated weathering. It is critical to establish the dependence of damage on dose for individual properties of interest.

### Improved models and test procedures afford better prediction of outdoor service lifetimes of materials

Estimating the service life of materials used outdoors under multiple environmental stressors, especially solar UV radiation and ambient temperature, is critical in developing strategies to improve their current service lifetimes despite changes in the UV radiation environment and global climate (SDG 9.4). This requires reliable laboratory-accelerated weathering procedures that estimate durability under specific exposure conditions as well as good lifetime models that yield good estimates of service lifetimes from weathering data [492]. However, acceleration of photo-damage in the laboratory has to ensure that wavelengths and intensities of radiation used, attain the same degradative mechanisms as solar exposure [489, 493].

Recent work has focused on two modelling approaches: (1) A model that consists of a series of terms, typically one for each environmental stressor, usually following the observed behaviour for that stressor (e.g., temperature with the Arrhenius model; see recent versions in e.g., [489, 490, 493, 494]. The challenge with this approach is in interpreting the interacting effects between different environmental stressors (e.g., temperature and moisture may have a synergistic effect). (2) A second approach is to use Bayesian statistics along with a database of rate constants obtained under specific multiple stress conditions (e.g., a controlled temperature, humidity, as a function of UV radiation dose) to construct the model. The advantage of the latter method is that it allows for the interacting effects between different environmental stressors to be taken into account. While this approach can predict the outdoor behaviour with a known degree of uncertainty, it lacks any physico-chemical insight into the behaviour of the material. This approach was successfully demonstrated with stabiliser-free (no additives to obtain rapid degradation) polyethylene (PE), the highest volume plastic used globally. The study in question was based on laboratory-accelerated weathering simulating field exposure conditions of Florida, United States [495, 496]. Both of these approaches are relevant and will be required to fully realise the potential of prediction modelling for service life of materials used outdoors.

## Linkages between COVID-19, solar UV radiation, and the Montreal Protocol

Coronavirus disease 2019 (COVID-19) is an infectious disease caused by severe acute respiratory syndrome coronavirus 2 (SARS-CoV-2). It was first identified in December 2019 in China and has resulted in a pandemic affecting the entire world, forcing governments to take measures to mitigate the spread of the virus. This section contributes to SDG 3 and assesses the linkages between COVID-19, solar UV radiation, and the Montreal Protocol. The focus is on the extent to which solar UV radiation can deactivate SARS-CoV-2 particles that are contaminating outdoor surfaces. This is the most relevant transmission path that is potentially affected by the Montreal Protocol: by preventing large increases in harmful UV-B radiation (Sect. 2.7), the Montreal Protocol has also modified the available germicidal UV-B radiation from the sun.

According to assessments by the World Health Organization [497], the European Centre for Disease Prevention and Control [498], and U.S. Centers for Disease Control and Prevention [499], COVID-19 is mainly transmitted from person to person through large respiratory droplets and aerosols (small droplets with diameters ≤ 5 μm [500]) generated by breathing, sneezing, and coughing. The relative role of large droplets *vs* aerosols is still unclear, but virus-containing aerosols can penetrate more deeply into the lungs [500]. Indirect transmission through fomites (defined as inanimate objects carrying pathogens) that have been contaminated by respiratory secretions is considered possible [501], although, so far, has not been conclusively documented [498].

Solar UV-B radiation is one of the factors affecting the lifetime of SARS-CoV-2 on surfaces contaminated by the virus as well as in respiratory droplets. Inactivation times (greater than 10 min as shown below) are considered too long to protect against transmission among people who are in close proximity outdoors [502]. The distinction between transmission via respiratory droplets and fomites is difficult to discern because persons who come into contact with potentially infectious surfaces often also have close contact with an infectious individual. Still, fomite transmission is considered a possible mode of transmission for SARS-CoV-2, given consistent findings about environmental contamination in the vicinity of infected cases [501] and the fact that other coronaviruses and respiratory viruses can transmit this way [497, 503]. As of this writing (September 2020), it is not possible to provide exact numbers on the prevalence of the different transmission pathways. For example, there are no reliable estimates on infections resulting from exposure to respiratory droplets *vs* fomite transmission.

A recent review ([504] and references therein) found very few examples of outdoor transmission of COVID-19 in everyday life situations among *ca.* 25,000 cases considered, and the authors concluded that the outdoor environment presents a very low risk of transmission of COVID-19. For example, in an analysis of COVID-19 outbreaks in China involving 1245 confirmed cases and 318 outbreaks only a single outbreak with two cases could be traced to outdoor transmission [505]. These studies support the currently prevailing view that most transmissions occur indoors where there is essentially no exposure to UV-B solar radiation.

Other topics discussed in this section include the relationship of the concentration of the vitamin D marker in serum, 25(OH)D, and/or sun exposure on the incidence or severity of COVID-19; the effect of air pollution on COVID-19 health outcomes; the effect of the economic slowdown resulting from the pandemic on air pollution and UV radiation at the surface; and the potential effects of changes in UV radiation resulting from actions taken to control the COVID-19 pandemic on terrestrial ecosystems and safe drinking water.

### SARS-CoV-2 particles can remain viable on surfaces for several days with their lifetime depending on surface material and ambient temperature

Several studies have shown that SARS-CoV-2 particles can remain viable on porous and non-porous surfaces for several days [506–510]. Infectious viruses have been recovered from plastic, glass, and stainless steel surfaces after 3 days [506], 7 days [507] and 28 days [508]. Viruses remain viable on banknotes between 4 [507] and up to 28 days when the ambient temperature is maintained at 20 °C [508]. On the outer layer of a surgical mask, infectious viruses can survive up to 6 days after contamination. Increasing the ambient temperature drastically reduces the survivability of virus particles on all surfaces to as little as 24 h at 40 °C [508]. As we show below, these long lifetimes of SARS-CoV-2 particles decrease greatly upon exposure to UV-C and UV-B radiation. Also some deactivation by UV-A radiation currently cannot be ruled out [511]. Research on lifetimes focuses on indoor surfaces and mainly in health-care facilities [512–514] where no solar UV-B radiation is present and rigorous biocidal disinfection routines are followed.

We note that most experiments on the survival of human coronaviruses on fomites (e.g., [506]) have used large numbers of viable virus particles (e.g., 10^4^–10^6^ per millilitre of collection medium), resulting in concentrations of viruses that may have been unrealistically high compared to those in droplets in real-life situations [515]. Hence, the viral load in fomites from infected individuals may be insufficient to cause a high rate of infection.

### UV-C radiation from artificial light sources is commonly used for disinfection and its germicidal property makes it much more effective than solar UV-B radiation

UV radiation has been used as a disinfectant in a wide number of situations since the late 1800s [516], including for purification of water, air, food, working surfaces and in laboratory experiments [517–521, 354]. More recently it has also been used as a disinfection tool against pathogens including SARS-CoV-2 [522]. UV-C radiation is a more effective disinfectant than UV-B radiation [523]. However, solar UV-C radiation is filtered out by Earth’s atmosphere, leaving the potential germicidal effect to the UV-B part in solar radiation reaching Earth’s surface.

### The germicidal effect of solar UV radiation depends on many factors, resulting in a large range of possible inactivation times between a few minutes and several hours

While the effect of sunlight on the survival of virus particles deposited on fomites is significant, we emphasise that this inactivation has likely a negligible effect on the progression of the COVID-19 pandemic: the available evidence suggests that fomite transmission from outdoor surfaces is one of the less important transmission pathways [504]. As COVID-19 emerged less than 10 months ago at the time of writing, research into the germicidal effect of solar radiation on SARS-CoV-2 viruses is still incomplete. We assess the current state of knowledge on this matter, which is rapidly evolving. The theoretical background is provided in the Online Resource (Supplementary Material 2).

To date, there are four studies that have estimated the inactivation time *t*_10_, which is defined as the time that reduces the number of viable SARS-CoV-2 viruses to 10% of its initial number upon exposure to solar UV radiation [502, 524–526]. Ratnesar-Shumate et al*.* [524] used a solar simulator to determine *t*_10_ for SARS-CoV-2 virus particles that were first suspended in either simulated saliva or a culture medium and then dried on stainless steel surfaces. The solar simulator produced spectral irradiance resembling noon-time solar spectra at 40° N latitude (e.g., Philadelphia, Ankara, Beijing) for three seasons: 21 June (solar zenith angle, SZA of 16.5°), 21 February (SZA = 50.6°) and 21 December (SZA = 63.4°). Inactivation times *t*_10_ for virus particles embedded in saliva were 6.8, 8.0 and 12.8 min for the three spectra, respectively. For viruses enclosed in the culture medium, *t*_10_ was about twice as long: 14.3 and 17.6 min for the spectra simulated for 21 June and 21 February, respectively.

Using the same solar simulator, Schuit et al*.* [525] determined *t*_10_ for aerosolised virus particles. For viruses suspended in saliva, *t*_10_ was 7.5 and 19 min for exposure to simulated noon solar spectra for 21 June (SZA = 16.5°) and 7 March (SZA = 45.0°), respectively. Uncertainties of the methods used by Ratnesar-Shumate et al*.* [524] and Schuit et al*.* [525] are discussed in the Supplementary Material 2.

Sagripanti and Lytle [526] and Herman et al*.* [502] used a more indirect method to estimate *t*_10_ based on the inactivation dose at 254 nm (a wavelength in the UV-C produced by a mercury lamp) and an “action spectrum”. This spectrum quantifies the biological effectiveness of radiation as a function of wavelength and is used to compute inactivation by solar UV radiation from inactivation at 254 nm. Both studies used an action spectrum that was developed 15 years ago [523]. This spectrum is the average of spectra from many virus species, but notably not including coronaviruses. The indirect method allows calculating *t*_10_ as a function of latitude, season, time of the day, and total ozone column (TOC). The method and its uncertainty are explained in more detail in the Supplementary Material 2.

Sagripanti and Lytle [526] calculate a *t*_10_ for Philadelphia (39.9° N) of 22 min at noon on the summer solstice (21 June), 38 min at the fall equinox, 63 min at the spring equinox, and > 300 min at the winter solstice (21 December). Inactivation times *t*_10_ calculated by Herman et al*.* [502] for viruses adhered to fomites oriented horizontally under clear skies are less than 8, 20, and 60 min for SZA less than 20°, 40°, and 60°, respectively. These times are generally smaller than those determined by Sagripanti and Lytle [526], mostly due to the difference in the inactivation dose at 254 nm assumed by the two studies.

We emphasise that the two indirect studies use the action spectrum by Lytle and Sagripanti [523], which ends at 320 nm. Hence, both studies assume that wavelengths in the UV-A region do not inactivate SARS-CoV-2. This assumption is consistent with the work by Darnell et al*.* [527] who demonstrated that UV-A radiation does not damage SARS-CoV-1 (a virus similar to SARS-CoV-2), but contradicts more recent work [511], suggesting that UV-A *does* contribute to the inactivation of SARS-CoV-2. It has also been shown that the viruses can be damaged by peroxides and other reactive oxygen species, which are created by UV-A radiation [528]. Whether a similar mechanism also affects SARS-CoV-2 has not yet been determined. If SARS-CoV-2 were indeed sensitive to UV-A radiation, inactivation times would increase more slowly with SZA as summarised above. Hence, solar radiation would be efficient in deactivating SARS-CoV-2 also at latitudes higher than 60° where the studies discussed above indicate inactivation times in excess of two hours. Until the action spectrum for deactivation of SARS-CoV-2 is available, activation times at large SZAs or high latitudes remain uncertain.

In general, the number of viable virus particles decreases exponentially when exposed to germicidal radiation, which implies that the time *t*_1_ required to reduce the number of viable virus particles to 1% of the initial number is twice as long as *t*_10_ (see Supplementary Material 2). In practice, the time difference between *t*_10_ and *t*_1_ can be considerably longer [526, 529, 530] because viruses in a real-world setting are embedded in a matrix of body fluids (e.g., saliva and mucus) or foreign objects, which partially shield viruses from exposure. Clustered populations of viruses can also protect each other from exposure to radiation [531]. Hence, there are large uncertainties in extrapolating *t*_10_ to *t*_1_ and beyond (e.g., 0.1% and 0.0001% survival for disinfection and sterilisation levels, respectively). This is particularly the case for virus particles that are embedded in porous materials, such as face masks and clothing, or otherwise shielded from radiation.

In summary, the uncertainty of inactivation times for SARS-CoV-2 is large and depends on many factors including uncertainties of the experiments used for determining inactivation times and the matrix in which the virus is embedded. Based on the studies discussed above [502, 524–526], we conclude that 90% of SARS-CoV-2 virus will be inactivated by solar UV radiation within 4 to 20 min under optimal conditions for SZA ≤ 40°. These times will become considerably larger for SZA > 40°, during cloudy conditions, if surfaces are not directly irradiated by sunlight, or if virus particles are shielded from solar exposure by other means (absorption by matrix material, deposition on a porous material, shade). Hence, while solar radiation helps to disinfect surfaces or exhaled aerosol contaminated with SARS-CoV-2 particles, one cannot rely on the sun’s germicidal effect in general and, in particular, early and late in the day, during winter, or at high latitudes during all seasons.

### The Montreal Protocol has prevented increases in solar UV radiation that inactivates SARS-CoV-2 viruses, but there is no evidence that this additional inactivation would have had a tangible effect on the progression of the COVID-19 pandemic

While the Montreal Protocol has prevented run-away increases in solar UV radiation [64, 532], it has thereby also affected the inactivation rate of pathogens exposed to UV radiation. According to McKenzie et al*.* [64], the Montreal Protocol has averted increases of erythemal (sunburning) irradiances by approximately 20% between the early 1990s and 2018 at mid-latitudes. This conclusion may imply that inactivation times of SARS-CoV-2 viruses would be about 20% shorter today if the MP had not been implemented, i.e., in the “World Avoided” scenario [8]. However, the effect from changes in stratospheric ozone avoided by the Montreal Protocol is about a factor of 1.75 larger for damage to DNA [533] than it is for sunburn. (The radiation amplification factor (RAF), which is defined as the relative fractional change in effective UV irradiance with fractional change in TOC, is about 2.1 for DNA damage and 1.2 for erythema [534]; the ratio of 2.1 and 1.2 is 1.75). In turn, this factor may imply that the Montreal Protocol has reduced the germicidal effectiveness of solar UV radiation by as much as 35% (1.75 times 20%). The actual percentage is still unknown, as the RAF for inactivation of SARS-CoV-2 (which has a RNA rather than a DNA genome) has not yet been established.

Since relevant information is still lacking for making an exact assessment, we estimate that due to the Montreal Protocol inactivation times for SARS-CoV-2 are 20% to 35% today compared to the early 1990s. However, it is unlikely that this effect has any tangible consequences on COVID-19 infections, as transmissions through fomites have not been conclusively documented [498] and outdoor infections are the exception [504, 505]. We conclude, based on the information currently available, that the effect of actions prompted by the Montreal Protocol on the progression of COVID-19 via its effect in modifying the germicidal properties of solar UV radiation is either small or negligible. The far-reaching, positive outcomes of the successful implementation of the Montreal Protocol for life on Earth (Sects. 2–7) outweigh any potential advantage for disinfection by higher amounts of solar UV radiation.

### Ultraviolet radiation, vitamin D and risk and severity of COVID-19

The effects of UV radiation and/or vitamin D on the immune system (Sect. 3.10) suggest that they may influence the risk or severity of COVID-19. Severe cases of COVID-19 are thought to be caused by an over-reactive, pro-inflammatory immune response to SARS-CoV-2 that leads to multi-organ failure [535]. In most people, the cells of the immune system coordinate their response to eliminate the virus and limit damage to the host. White blood cells (specifically B cells) will also be activated to produce neutralising SARS-CoV-2 antibodies that would ideally provide long-lived protection from COVID-19. Although it is too early to know if this normal immune response is occurring in those infected with SARS-CoV-2, a recent Icelandic study showed that over 90% of people who tested positive to the virus had antibodies, which were still present for at least 120 days after diagnosis, with no decrease in levels [536].

For unknown reasons, in ca 2% of infected people the immune response is aberrant and uncontrolled; the resultant “cytokine storm” produces significant tissue damage. The recent success of immune-suppressive anti-inflammatories (e.g., dexamethasone) in treating severe cases of COVID-19 [537] highlights the importance of immune suppression, although whether exposure to immune-modulatory UV radiation or enhancing vitamin D status would have a similar beneficial effect remains unclear.

Some, although not all, studies conducted on populations rather than individuals support a role of UV radiation or vitamin D on the progression of COVID-19. A study of 88 countries found that increasing proximity to the equator was correlated with lower death rates from COVID-19 [538]. A second international study including 359 countries or regions within countries found a significant negative correlation between the average maximum UV index during the period of the study and the incidence of COVID-19 [539]. Two studies within the United States suggest reduced incidence or transmission in areas with high ambient UV radiation [540, 541]. Conversely, a study in China found that no climate factors, including UV radiation, were associated with the cumulative incidence or rate of infection of COVID-19 [542], and a study in Spain found a positive correlation between daily sunshine hours and COVID-19 incidence [543]. Further, an Indonesian study found a positive association between daily hours of sunshine and recovery from COVID-19, but no association with incidence or death [544].

These population-level studies need to be viewed with caution, as there are many other possible explanations for inter-country and inter-region variability that cannot be adequately controlled. Observational studies provide only limited support for a role of vitamin D. The United Kingdom National Institute for Health and Care Excellence (NICE) reviewed the available evidence in June 2020, at which time five studies had been published. While four studies suggested an association between concentrations of 25(OH)D concentration in serum and risk of COVID-19, all five studies were found to be at high risk of bias [545]. Several studies have been published since this review, but these mostly have similar limitations, notably a lack of simultaneous testing for 25(OH)D and SARS-CoV-2 infection. Several studies have identified a possible association between low vitamin D status and increased risk of COVID-19 [546–548]. A study using anonymised electronic health records from the University of Chicago health system found that vitamin D deficiency in the year prior to testing was associated with SARS-CoV-2 positivity [546]. An analysis of data from the UK Biobank cohort found no association between SARS-CoV-2 positivity and concentration of 25(OH)D in serum, but 25(OH)D was measured much earlier than the time that the infection with SARS-CoV occurred. A large study using pathology records from the United States observed that 25(OH)D concentration measured in the year prior to SARS-CoV-2 testing was inversely associated with virus positivity, but there was no control for potential confounders such as body mass index or co-morbidities [546–548]. A study using genetically determined, rather than measured 25(OH)D, found no link with SARS-CoV-2 positivity, or severe COVID-19 disease, suggesting that associations with vitamin D may not be causal [549].

Some studies have shown associations with markers of severity of COVID-19 infection, such as hospitalisation, admission to intensive or critical care units, need for ventilation, or death [547, 550, 551]. Adjustment for confounders, such as frailty, was variable and clinical trials are needed to confirm the role of vitamin D in the severity of COVID-19. A pilot trial (without placebo control) of administration of 25(OH)D in 76 Spanish patients hospitalised with COVID-19 found a markedly reduced risk of intensive care unit admission in the intervention group (1/50 patients (2%)) compared with the control group (13/26 (50%)) [552] but this suggestive finding needs verification in larger double-blind placebo-controlled trials.

Considering the search for a vaccine to prevent COVID-19, it is important to determine whether there is evidence that exposure to UV radiation, or vitamin D status, may influence the response to vaccination. Exposure to UV-B radiation suppresses both the cell-mediated and humoral (antibody-driven) immune response, both of which are the indirect targets of new vaccines against SARS-CoV-2. However, this area is complex; the nature of the immune response being provoked and the amount and timing of exposure to UV radiation may influence any impacts on vaccine effectiveness [553]. Studies of vaccination against other viruses may be informative, but there are few randomised controlled trials. In a study of hepatitis B vaccination in British army recruits, low vitamin D status at the time of vaccination was associated with poorer vaccine response [554]. However, neither vitamin D supplementation nor exposure to solar-simulated UV radiation influenced immune response [554]. This lack of effect is in line with the conclusions from an earlier meta-analysis of the link between vitamin D and response to influenza vaccine, in which no overall association was identified [555]. Given the current pandemic, and indeed the influence of infectious diseases and population health and mortality in general, more information is needed.

### Recent studies suggest that poor air quality worsens the severity of COVID-19 health outcomes

Meteorological factors associated with the spread and severity of COVID-19 have been investigated [556–558] and it appears that poor air quality is associated with worsened health outcomes [559–563]. Air quality can also be related to changes in UV radiation resulting from the pandemic (Sect. 7.4). The correlation between air quality and health outcomes is consistent with the notion of respiratory damage from both COVID-19 and air pollution, but the methods used in these studies have weaknesses [559, 564], which prevent a quantitative assessment of the link between air pollution and mortality from COVID-19.

### The economic slowdown resulting from the 2020 pandemic reduced pollution by particulate matter but increased ambient ozone in many regions

Suspended particulate matter (PM2.5) generally showed a decrease during the COVID-19 pandemic, but concentrations of ambient ozone in polluted regions have remained constant or even increased, due to increased photochemical reactivity. Reductions in emissions due to the COVID-19 pandemic show similar effects on air quality to those observed due to pollution control measures in the last decade. For example, data on air quality were analysed from *ca.* 1600 locations in China. Comparing data from January and February 2020 with the same period in 2019 showed a general decrease in the concentrations of CO, SO_2_, NO_2_, and PM2.5, while ambient ozone, which is produced photochemically, has increased [565]. The mechanisms behind this increase in ozone are probably like those invoked for the long-term observations and are, in part, related to changes in UV radiation. Similar results for China were found by others [566–572].

Reductions in pollutant emissions have been reported for many countries, e.g., Brazil [573, 574], Canada [575], Ecuador [576], India [577, 578] and the USA. [579]. Rodriguez-Urrego and Rodrigues-Urrego [580] compared concentrations of PM2.5 before and during quarantine for 50 capital cities of the world. Reductions were most evident in cities that implemented aggressive measures to prevent spread of the virus. Satellite observations showed decreases in NO_2_ [581] in many countries, albeit less in those with less strict COVID-19 policies. Venter et al*.* [582] analysed satellite observations and data from > 10,000 ground stations, reporting large decreases in NO_2_ and PM2.5 but with marginal increases in ground-level ozone. These and other [583–585] observations will provide a valuable dataset to evaluate and improve air quality models [388, 389]. Surveys show that most people have perceived a substantial improvement in air pollution during COVID-19 shutdown periods in many countries [586].

### Reductions in air pollution resulting from measures in response to COVID-19 have increased UV and visible radiation for terrestrial ecosystems

Decreases in air pollution during the COVID-19 pandemic are likely to have exposed terrestrial ecosystems to temporarily increased solar UV and visible radiation, which could have benefited crop plants by increasing their resilience to other environmental stresses and crop diseases through stimulated antioxidant production or protective morphological features [587, 588]. It is also conceivable that this increase in UV-B radiation could have caused negative effects by inducing cellular damage and reducing reproductive performance [169]. The overall net effect on crops and terrestrial ecosystems of the increased solar UV and visible radiation due to the temporary improvement in air pollution prompted by the pandemic is not yet known. On the other hand, exposure to more intense UV-B radiation, as would have occurred in the absence of the Montreal Protocol, would have had negative effects on crops by exceeding their UV-B-protective responses, and this effect would have been further magnified by decreases in air pollution as a consequence of COVID-19 restrictions.

### Exposure to solar UV-B radiation inactivates viruses in surface waters, drinking water supplies, and sewage treatment plants, suggesting that the threat of COVID-19 transmission through these avenues is low but specific studies are still lacking

Similar to terrestrial ecosystems, where air pollution has declined because of restrictions in response to the 2020 pandemic, inland and coastal aquatic ecosystems have been exposed to increased solar UV radiation. This increase in UV radiation has benefits in reducing aquatic pathogens, including viruses, given their sensitivity to inactivation by both UV-B radiation and reactive oxygen species produced by UV-A radiation [286–291]. While there is evidence that other coronaviruses can survive in water for days to weeks [589], there are currently few reports on the presence of SARS-CoV-2 viruses in sewage, surface waters, groundwater, or drinking water supplies [590, 591]. The risk of contamination through these means is therefore considered low [592]. This may be a consequence of the SARS-CoV-2 virus being an enveloped virus with a delicate outer membrane that makes it more susceptible to environmental degradation by solar UV-B radiation and other factors such as chlorination or ozonation of drinking water. There is currently also no evidence of transmission of COVID-19 through human sewage. Conventional sewage treatment plants are designed to inactivate viruses, including SARS-CoV-2, particularly when sewage has long residence times in outdoor treatment tanks with prolonged (several weeks) exposure to solar UV-B radiation [592]. For further information on how variations in exposure to UV radiation may affect pathogens in the aquatic environment, see Sect. 5.12.

## Electronic supplementary material

Below is the link to the electronic supplementary material.

Supplementary file1 (PDF 451 kb)

Supplementary file2 (PDF 277 kb)

## Data Availability

All data generated or analysed are included in this published article [and its supplementary information files].

## Footnotes

1 Representative Concentration Pathways are greenhouse gas concentration (not emission) trajectories adopted by the Intergovernmental Panel on Climate Change (IPCC) for its fifth Assessment Report. The pathways are used for climate modelling and research. They describe four climate futures, which differ in the amount of greenhouse gases that are emitted in years to come. The four RCPs, RCP 2.6, RCP 4.5, RCP 6, and RCP 8.5, are named after a possible range of radiative forcing values in the year 2100 relative to pre-industrial values (+ 2.6, + 4.5, + 6.0, and + 8.5 W m^−2^, respectively).

2 The SAM is the leading mode of Southern Hemisphere extratropical climate variability, describing a see-saw of atmospheric mass between the mid- and high latitudes, with corresponding impacts on the strength of the circumpolar westerly winds. A positive SAM index corresponds to a poleward shift of the maximum wind speed, which results in weaker-than-normal westerly winds in the southern mid-latitudes.

3 Shared socio-economic pathway (SSP) scenarios are greenhouse gas concentration (not emission) trajectories adopted by the Intergovernmental Panel on Climate Change (IPCC) for its sixth Assessment Report. The pathways are used for climate modelling and research, and anticipate different socio-economic developments and political environments. They describe five climate futures, which differ in the amount of greenhouse gases that are emitted in years to come. The five SSPs SSP1-1.9, SSP1-2.6, SSP2-4.5, SSP3-7.0, and SSP5-8.5, are named after a possible range of radiative forcing values in the year 2100 relative to pre-industrial values (+ 1.9, + 2.6, + 4.5, + 7.0, and + 8.5 W m^−2^, respectively), and have some equivalence to the RCPs used in the fifth Assessment Report.

4 PFASs, most of which have different properties from TFA, are widely used commercially in coatings, paints, non-stick utensils, electronics, and medical devices, and (historically) in fire-fighting foams, They are stable to breakdown in the environment and are found in many environmental compartments and in animals, including humans.
