# Supplementary material for: Environmental effects of stratospheric ozone depletion, UV radiation, and interactions with climate change: UNEP Environmental Effects Assessment Panel, Update 2020
Source: Photochem Photobiol Sci. 2021 Jan 20;20(1):1–67. doi: 10.1007/s43630-020-00001-x (PMC7816068; doi:10.1007/s43630-020-00001-x)
Supplement: Supplementary file 1 — Supplementary file1 (PDF 451 kb) [file 43630_2020_1_MOESM1_ESM.pdf]

Supplementary Material 1: Online Resource for Section 7.8 of

Environmental effects of stratospheric ozone depletion, UV radiation, and interactions with climate change: UNEP Environmental Effects Assessment Panel, update 2020

R.E. Neale, P.W. Barnes, T.M. Robson, P.J. Neale, C.E. Williamson, R.G. Zepp, S.R. Wilson, S. Madronich, A.L. Andrady, A.M. Heikkilä, G.H. Bernhard, A.F. Bais, P.J. Aucamp, A.T. Banaszak, J.F. Bornman, L.S. Bruckman, S.N. Byrne, B. Foereid, D.-P. Häder, L.M. Hollestein, W.-C. Hou, S. Hylander, M.A.K. Jansen, A.R. Klekociuk, J.B. Liley, J. Longstreth, R.M. Lucas, J. Martinez-Abaigar, K. McNeill, C.M. Olsen, K.K. Pandey, L.E. Rhodes, S.A. Robinson, K.C. Rose, T. Schikowski, K.R. Solomon*, B. Sulzberger, J.E. Ukpebor, Q.-W. Wang, S.-Å. Wängberg, C.C. White, S. Yazar, A.R. Young, P.J. Young, L. Zhu, M. Zhu

*Corresponding author: K.R. Solomon, Centre for Toxicology, School of Environmental Sciences, University of Guelph, Guelph, Canada
Email: ksolomon@uoguelph.ca

Published in Photochemical & Photobiological Sciences

Air quality

This supplement provides additional toxicity data to Section 7.8 for other short-chain perfluorinated acids that might be formed from breakdown of products that fall under the purview of the Montreal Protocol.

Fluorocarbon (HCFC-225ca), a chemical regulated under the Montreal Protocol, breaks down to form the three-carbon perfluorinated acid PFPrA, which was detected in Arctic ice-cores along with TFA and PFBA [1]. There are some data on toxicity for toxicity of PFPrA (C-3) and PFBA (C-4) to mammals. The oral LDLo (a dose causing minimal toxicity) of PFPrA in the rat was reported as 750 mg kg^{-1} body weight [2], indicative of low *de minimis* hazard. No oral toxicity data for mammals were available for PFBA [3], but unlike the longer chain PFASs, PFBA appears not to be bioaccumulative in marine mammals [4], so it might present a lesser risk to mammals than longer chain analogues.

Pickard et al. [1] did point out the paucity of ecotoxicity data on PFPrA and PFBA, but there are some data for aquatic algae (*Raphidocelis subcapitata* and *Chlorella vulgaris*), an aquatic plant (*Lemna gibba*), crustaceans (*Daphnia magna* and *D. pulicaria* [5] and for the rotifer *Brachionus calyciflorus* [6]. Data for several short- and longer-chain PFASs are summarised in Table 1S.

Table 1S. Toxicity endpoints in fresh-water aquatic plants and animals for PFASs of varying chain length > 2 carbons

PFAS (number of carbons)	Concentration that inhibits growth (IC) by 10% or 50% in µg L^{-1}			Median lethal concentration (LC50) and concentration causing 10% mortality (LC10) in µg L^{-1}		
Species	*Raphidocelis subcapitata*[a]	*Chlorella. vulgaris*[a]	*Lemna. Gibba*[a]	*Daphnia magna*[a]	*D. pulicaria*[a]	*Brachionus calyciflorus*[b]
PFPrA (C3)	IC10 = 1.76 x 10^4	IC 10 = 1.44 x 10^4	IC10 = 6.73 x 10^6	LC50 > 1.00 x 10^6	LC50 > 1.00 x 10^6	LC50 = 8.0 x 10^4
PFBA (C4)	IC50 = 6.21 x 10^5	IC50 = 7.28 x 10^5	IC50 > 1.01 x 10^6	LC50 > 1.01 x 10^6	LC50 > 1.01 x 10^6	LC50 = 1.1 x 10^5

Table 1S. Toxicity endpoints in fresh-water aquatic plants and animals for PFASs of varying chain length > 2 carbons

PFPeA (C5)	IC50 > 1.00 x 10^6	IC50 > 1.00 x 10^6	IC50 > 1.00 x 10^6	LC50 > 1.00 x 10^9	LC50 > 1.00 x 10^6	LC50 = 1.3 x 10^5
PFHxA (C6)	NA	NA	NA	NA	NA	LC50 = 1.4 x 10^8
PFHpA (C7)	IC50 > 1.02 x 10^6	IC50 > 1.02 x 10^6	IC50 > 1.02 x 10^6	LC50 > 1.02 x 10^6	LC50 > 1.02 x 10^6	NA
PFOA (C8)	IC10 = 5.38 x 10^4	IC10 = 5.80 x 10^3	IC10 = 2.15 x 10^4	LC10 = 1.48 x 10^5	LC10 = 9.77 x 10^4	NA
PFNA (C9)	NA	NA	IC10 = 5.89 x 10^7	LC10 = 7.47 x 10^7	NA	NA
PFDA (C10)	IC10 = 6.84 x 10^4	IC10 = 1.18 x 10^4	IC10 = 2.31 x 10^4	LC10 = 5.50 x 10^4	LC10 = 9.77 x 10^4	NA

[a]Data from ref. [5], [b]Data from ref. [6]

All the toxicity values in Table 1S are based on nominal concentrations in the exposure solutions. Several were greater than the maximum concentration tested (1 x 10^6 µg L^{-1}). Even for sensitive endpoints, such as concentrations causing a 10% reduction in growth (IC10) or concentrations causing 10% mortality in animals (LC10), there were wide margins of exposure (3–6 orders of magnitude) between these sensitive toxicity values and the greatest measured concentrations of PFPrA and PFBA in the ice cores from the Arctic. This is indicative of *de minimis* risks from environmental exposures of aquatic animals to these compounds.

References

1. Pickard, H. M., Criscitiello, A. S., Persaud, D., Spencer, C., Muir, D. C., Lehnherr, I., et al. (2020). Ice core record of persistent short-chain fluorinated alkyl acids: Evidence of the impact from global environmental regulations. *Geophysical Research Letters, 47*, e2020GL087535S, https://doi.org/10.1029/2020GL087535.
2. PubChem (2020). Pentafluoropropionic-acid. https://pubchem.ncbi.nlm.nih.gov/compound/Pentafluoropropionic-acid. Accessed July 2020.
3. PubChem (2020). Heptafluorobutyric-acid. https://pubchem.ncbi.nlm.nih.gov/compound/Heptafluorobutyric-acid. Accessed July 2020.
4. Spaan, K. M., van Noordenburg, C., Plassmann, M. M., Schultes, L., Shaw, S., Berger, M., et al. (2020). Fluorine mass balance and suspect screening in marine mammals from the Northern Hemisphere. *Environmental Science & Technology, 54*, 4046-4058, https://doi.org/10.1021/acs.est.9b06773.
5. Boudreau, T. M. (2002). *Toxicity of Perfluorinated Organic Acids to Selected Freshwater Organisms under Laboratory and Field Conditions*. M.Sc., University of Guelph, Guelph.
6. Wang, Y., Niu, J., Zhang, L., & Shi, J. (2014). Toxicity assessment of perfluorinated carboxylic acids (PFCAs) towards the rotifer *Brachionus calyciflorus*. [Research Support, Non-U.S. Gov't]. *The Science of the Total Environment, 491-492*, 266-270, https://doi.org/10.1016/j.scitotenv.2014.02.028.
