# Supplementary material for: Environmental effects of stratospheric ozone depletion, UV radiation, and interactions with climate change: UNEP Environmental Effects Assessment Panel, Update 2020
Source: Photochem Photobiol Sci. 2021 Jan 20;20(1):1–67. doi: 10.1007/s43630-020-00001-x (PMC7816068; doi:10.1007/s43630-020-00001-x)
Supplement: Supplementary file 2 — Supplementary file2 (PDF 277 kb) [file 43630_2020_1_MOESM2_ESM.pdf]

Supplementary Material 2: Online Resource for Section 9.3 of

Environmental effects of stratospheric ozone depletion, UV radiation, and interactions with climate change: UNEP Environmental Effects Assessment Panel, update 2020

R.E. Neale, P.W. Barnes, T.M. Robson, P.J. Neale, C.E. Williamson, R.G. Zepp, S.R. Wilson, S. Madronich, A.L. Andrady, A.M. Heikkilä, G.H. Bernhard*, A.F. Bais, P.J. Aucamp, A.T. Banaszak, J.F. Bornman, L.S. Bruckman, S.N. Byrne, B. Foereid, D.-P. Häder, L.M. Hollestein, W.-C. Hou, S. Hylander, M.A.K. Jansen, A.R. Klekociuk, J.B. Liley, J. Longstreth, R.M. Lucas, J. Martinez-Abaigar, K. McNeill, C.M. Olsen, K.K. Pandey, L.E. Rhodes, S.A. Robinson, K.C. Rose, T. Schikowski, K.R. Solomon, B. Sulzberger, J.E. Ukpebor, Q.-W. Wang, S.-Å. Wängberg, C.C. White, S. Yazar, A.R. Young, P.J. Young, L. Zhu, M. Zhu

*Corresponding author: G.H. Bernhard, Biospherical Instruments Inc, California, USA
Email: bernhard@biospherical.com

Published in Photochemical & Photobiological Sciences

Linkages between COVID-19, solar UV radiation, and the Montreal Protocol

This supplement discusses the technical aspects of Sect. 9.3, provides the mathematical foundation for calculating inactivation times, and presents additional information on the uncertainties affecting these calculations.

S.1 Inactivation as a function of time

In general, the number of viable virus particles N decreases exponentially when exposed to germicidal radiation (either from artificial light sources or the Sun) for the time t [1,2]:

$$N = N_0 e^{-\alpha t}, \quad (1)$$

where N_0 is the number of viable particles at the start of the exposure and α is a decay constant. If we define t_{10} and t_1 as the times that reduce N to 10% and 1% of its initial number, respectively, Eq. (1) implies that t_1 is twice as long as t_{10} ($t_1 = f \times t_{10}$ with factor $f = 2$). Several experiments with various types of viruses suggest that f is larger than 2 ([3-5]). Sagripanti and Lytle [5] and Heßling *et al.* [6] attribute this effect to the fact that viruses in a real-world setting are embedded in a matrix of body fluids (e.g., saliva and mucus) or foreign objects, which partially shield viruses from exposure. Clustered populations of viruses can also protect each other from exposure to radiation [7]. Shielded particles have a smaller decay constant, and the true change of N with t is therefore better expressed as

$$N = \sum_i N_{0,i} e^{-\alpha_i t}, \quad (2)$$

where $N_{0,i}$ is the initial number of viable viruses within a specific matrix and α_i is the associated decay constant. For short irradiation times, the decay of N is dominated by the viruses that are shielded the

least from radiation and have the largest decay constant. Experiments with Ebola, Lassa, and influenza-A viruses indicate that the decay constant for several percent of the virus population may be 4 times as large than that of the least-shielded population ([5] and references therein).

S.2 Relationship between inactivation time, effective radiation dose and effective irradiance

Eq. (1) can be re-written as:

$$N = N_0 \mathrm{e}^{-kD} = N_0 \mathrm{e}^{-kE_e t}, \tag{3}$$

where D is the effective radiation dose that causes the inactivation, E_e is the effective irradiance, and k is a decay constant. Inactivation times t_{10} can be calculated with Eq. (3) with two methods, referred to as the direct and indirect method. The exponent of the last term of Eq. (3) is linear in E_e and t, which implies that doubling the effective irradiance will cut the inactivation time in half.

S.3 Uncertainty of the direct method to calculate inactivation times using a solar simulator

The direct method used by Ratnesar-Shumate *et al.* [8] and Schuit *et al.* [9] to calculate inactivation times using a solar simulator is described in Sect. 9.3. It is affected by uncertainties resulting from the preparation of the virus sample, the counting of viable virus particles, plus differences between spectra of the solar simulator and actual solar spectra. For example, Figure 2A of Ratnesar-Shumate *et al.* [8] suggests a significant difference between the simulated and the actual solar spectrum near 305 nm, which is close to the wavelength where the product of the solar and the action spectrum ($E(\lambda) \times A(\lambda)$) peaks. Since the dose $D_{10}(254)$ used by Herman *et al.* [1] is based on the results by Ratnesar-Shumate *et al.* [8], the inactivation times calculated by Herman *et al.* [1] with the indirect method (Sect. 9.3) are also affected by this uncertainty. A replication of these experiments by an independent group and using the sun as the light source instead of a solar simulator would help to better evaluate these uncertainties.

S.4 Calculation of inactivation times with the indirect method

For the indirect method, virus particles are exposed to an artificial light source (typically the monochromatic emission of a mercury lamp at 254 nm) and the dose $D_{10}(\lambda_r)$ that results in 10% survival is determined. This result is then extrapolated to survival under sunlight using an action spectrum $A(\lambda)$, which quantifies the biological effectiveness of radiation as a function of wavelength. Lastly, modelled spectra of solar irradiance $E(\lambda)$ for various solar zenith angles (SZAs) and total ozone columns (TOCs) are used to assess virus inactivation as a function of these parameters. The inactivation times t_{10} in minutes is calculated from these quantities [1]:

$$t_{10}[\mathrm{min}] = \frac{D_{10}(\lambda_r)}{60\mathrm{s} \times E_e} = \frac{D_{10}(\lambda_r)}{60\mathrm{s} \times \int E(\lambda) A(\lambda) \mathrm{d}\lambda}. \tag{4}$$

The action spectrum $A(\lambda)$ in Eq. (4) is normalised to one at the reference wavelength λ_r (typically 254 nm) for which $D_{10}(\lambda_r)$ was determined. For action spectra of interest here [10], the product of the solar spectrum and the action spectrum ($E(\lambda) \times A(\lambda)$) is significantly different from zero between 295 and 320 nm, and peaks between 300 and 310 nm, depending on the SZA.

The indirect method has larger uncertainties than the direct method using a solar simulator (section S.3) because neither the inactivation dose at 254 nm, $D_{10}(254)$, nor the action spectrum have been

established for SARS-CoV-2 viruses. Sagripanti and Lytle [5] have set $D_{10}(254)$ to 6.9 J m^{-2}, based on studies of other single-strand RNA viruses, including two viruses that are very similar to SARS-CoV-2: the murine hepatitis virus (MHV; $D_{10}(254)$) and the equine torovirus (EToV; $D_{10}(254)$). In contrast, Herman *et al.* [1] calculate $D_{10}(254)$ = 3.2 J m^{-2} from the solar simulator measurements by Ratnesar-Shumate *et al.* [8]. Values of $D_{10}(254)$ published for other coronaviruses range between 6 to 117,540 J m^{-2} [6]. This very large range is thought to be caused by absorption of the organic materials in which viruses were suspended during the experimental determination of $D_{10}(254)$. Even when removing studies that appear to be outliers, larger differences remain and these directly affect the accuracy of inactivation times calculated from $D_{10}(254)$ using the indirect method.

References

1. Herman, J., Biegel, B., & Huang, L. (2020). Inactivation times from 290 to 315 nm UVB in sunlight for SARS coronaviruses CoV and CoV-2 using OMI satellite data for the sunlit Earth. *Air Quality, Atmosphere & Health*, https://doi.org/10.1007/s11869-020-00927-2.
2. Blatchley, E. R. I., & Coohill, T. P. (2021). Ultraviolet Disinfection. In G. McDonnell, & J. Hansen (Eds.), *Disinfection, Sterilization, and Preservation, 6th ed.* (pp. 171-191). Philadelphia, USA: Wolters Kluwer.
3. Weiss, M., & Horzinek, M. C. (1986). Resistance of Berne virus to physical and chemical treatment. *Veterinary Microbiology, 11*(1-2), 41-49, https://doi.org/10.1016/0378-1135(86)90005-2.
4. Kariwa, H., Fujii, N., & Takashima, I. (2006). Inactivation of SARS coronavirus by means of povidone-iodine, physical conditions and chemical reagents. *Dermatology, 212*, 119-123, https://doi.org/10.1159/000089211.
5. Sagripanti, J. L., & Lytle, C. D. (2020). Estimated inactivation of coronaviruses by solar radiation with special reference to COVID-19. *Photochemistry and Photobiology, 96*(4), 731-737, https://doi.org/10.1111/php.13293.
6. Heßling, M., Hönes, K., Vatter, P., & Lingenfelder, C. (2020). Ultraviolet irradiation doses for coronavirus inactivation–review and analysis of coronavirus photoinactivation studies. *GMS hygiene and infection control, 15*, https://doi.org/10.3205/dgkh000343.
7. Kowalski, W. J., Bahnfleth, W. P., Raguse, M., & Moeller, R. (2020). The cluster model of ultraviolet disinfection explains tailing kinetics. *Journal of Applied Microbiology, 128*(4), 1003-1014, https://doi.org/10.1111/jam.14527.
8. Ratnesar-Shumate, S., Williams, G., Green, B., Krause, M., Holland, B., Wood, S., et al. (2020). Simulated sunlight rapidly inactivates SARS-CoV-2 on surfaces. *Journal of Infectious Diseases, 222*(2), 214-222, https://doi.org/10.1093/infdis/jiaa274.
9. Schuit, M., Ratnesar-Shumate, S., Yolitz, J., Williams, G., Weaver, W., Green, B., et al. (2020). Airborne SARS-CoV-2 is rapidly inactivated by simulated sunlight. *Journal of Infectious Diseases, 222*(4), 564-571, https://doi.org/10.1093/infdis/jiaa334.
10. Lytle, C. D., & Sagripanti, J. L. (2005). Predicted inactivation of viruses of relevance to biodefense by solar radiation. *Journal of Virology, 79*(22), 14244-14252, https://doi.org/10.1128/JVI.79.22.14244-14252.2005.
